# Intracellular Formation of Synthetic Peptide Nanostructures Causes Mitochondrial Disruption and Cell Death in Tumor Spheroids

**DOI:** 10.1002/advs.202412606

**Published:** 2025-05-26

**Authors:** Sarah Chagri, Konrad Maxeiner, Maria J. S. A. Silva, Lisa Förch, Julian Link, Patrick Roth, Raphael Meyer, Jana Fetzer, Anke Kaltbeitzel, Ingo Lieberwirth, Katharina Landfester, Manfred Wagner, David Y.W. Ng, Tanja Weil

**Affiliations:** ^1^ Max Planck Institute for Polymer Research 55128 Mainz Germany

**Keywords:** bioresponsive nanomaterials, mitochondrial disruption, peptide nanostructures, supramolecular assemblies, synthetic intracellular nanostructures, tumor spheroids

## Abstract

Supramolecular assemblies found in nature demonstrate the concept of creating functionality through structure formation. In recent years, these complex natural architectures have inspired the development of materials for the formation of synthetic nanostructures within living cells. These intracellular assemblies have the potential to modulate cellular processes, yet their specific effects on cellular metabolism and 3D cell networks, such as tumor spheroids, still remain underexplored. Herein, the study correlates the glutathione‐induced formation of synthetic nanostructures inside MDA‐MB‐231 triple‐negative breast cancer cells to the metabolic disruption and mitochondrial degradation observed in 2D cell culture, as well as to cell death and size decrease in a 3D tumor spheroid model. In 2D cell culture, material‐cell interactions are examined through live‐cell imaging and by quantifying changes in mitochondrial respiration. By studying the interplay between glutathione‐responsive cytosolic peptide assembly and the implications on the integrity of the mitochondrial network, as well as on 3D cell networks, the work advances the understanding of how synthetic intracellular nanofibers impact vital functions of living cells.

## Graphical abstract

Synthetic peptide nanostructures, formed within cancer cells in response to glutathione, lead to mitochondrial dysfunction and cell death. The nanostructures significantly damage mitochondrial networks, impair cellular respiration, and induce cell death in both 2D cultures and 3D tumor spheroids. These results advance the understanding of the biological impact of bioresponsive synthetic structure formation within living cells.

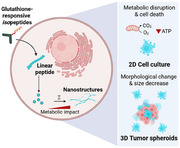

## 1 Introduction

Intracellular nanostructures, such as the cytoskeleton, are naturally occurring protein assemblies that regulate fundamental processes involved in cell metabolism, division, and motility.^[^ ^1^ ^]^ These intricate and dynamic protein assemblies span the cytosol, serving as a blueprint for the design of synthetic nanomaterials for intracellular structure formation. The creation of synthetic architectures within mammalian cells represents a milestone in synthetic biology and nanomedicine, as it offers a bottom‐up understanding of how natural intracellular assemblies can be replicated based on molecular design principles. This knowledge offers new avenues for understanding the impact of such assemblies on cellular integrity, function, and viability.^[^ ^2^ ^]^

Given the prevalence of the resistance to various chemotherapeutics in healthcare, the development of alternative strategies for effective treatment represents a vital objective in biomedical sciences.^[^ ^3^ ^]^ Supramolecular approaches that involve forming synthetic nanostructures inside cells represent a unique strategy for mechanically inducing cell death by disrupting vital cellular processes.^[^ ^4^ ^]^ The accumulation of larger aggregates inside cancer cells enhances the retention of synthetic materials, thereby improving pharmacokinetic properties and circumventing common drug resistance mechanisms, such as clearance driven by the overexpression of efflux pumps. However, preventing the premature assembly of the nanomaterial outside cells is crucial in the material design process. Tailoring synthetic nanomaterials to display bioresponsive properties ensures that the pro‐assembling compounds transform into active monomers only in the presence of specific endogenous cues, enhancing spatiotemporal control over assembly formation.^[^ ^2^ ^]^ Physiological stimuli, such as pH gradients^[^ ^5^ ^]^ intracellular enzymes^[^ ^6^ ^]^ and the high concentration of the reductive glutathione in the cytosol^[^ ^7^ ^]^ can be exploited to selectively induce structure formation. In cancerous cells, the intracellular glutathione concentration is often increased,^[^ ^8^, ^9^ ^]^ which contributes to multidrug resistance due to the glutathionylation and subsequent elimination of small molecule drugs.^[^ ^10^, ^11^ ^]^ This biological phenomenon can be used to design pro‐assembling precursors by introducing glutathione‐responsive trigger groups with cleavable disulfide bridges,^[^ ^6^, ^12^, ^13^ ^]^ enabling the controlled formation of synthetic nanostructures inside living cells upon reduction.

In recent years, our group has developed a series of peptide‐based pro‐assembling precursors that undergo a multistep conversion process in response to two specific environmental cues: the acidifying environment inside endosomes upon cellular uptake and the presence of reactive oxygen species (ROS) in the cytosol upon endosomal escape.^[^ ^4^, ^14^, ^15^ ^]^ Building upon this foundation, we have expanded the portfolio toward a glutathione‐responsive system that activates immune cells through the formation of intracellular peptide nanostructures.^[^ ^16^ ^]^ The glutathione‐induced peptide assembly within activated cytotoxic T cells stimulates T cell effector function, thereby increasing their cytotoxic activity toward cancer cells.^[^ ^16^ ^]^ Despite this progress, detailed studies on the concentration‐ and time‐dependent biological impacts of synthetic intracellular assemblies—particularly regarding their effects on cancer cell metabolism—have only recently emerged. It is therefore essential to investigate the uptake, distribution, and intracellular conversion of assembly precursors over time to elucidate how the timeline of in situ structure formation correlates with the impact on cellular function.

To date, the analysis of in situ intracellular structure formation has been mostly focused on cells cultured in conventional 2D monolayers.^[^ ^15^, ^16^ ^]^ However, findings derived from 2D cell cultures often fail to accurately replicate in vivo conditions, primarily due to their oversimplified cell‐cell and cell‐extracellular matrix interactions. In contrast, 3D cell networks more closely emulate the complex interactions observed in vivo. Notably, multicellular tumor spheroids exhibit key characteristics of solid tumors, including gradients in oxygen and nutrients from the outer cell layers to the hypoxic core, as well as high cell density.^[^ ^17^ ^]^

**Figure 1:**
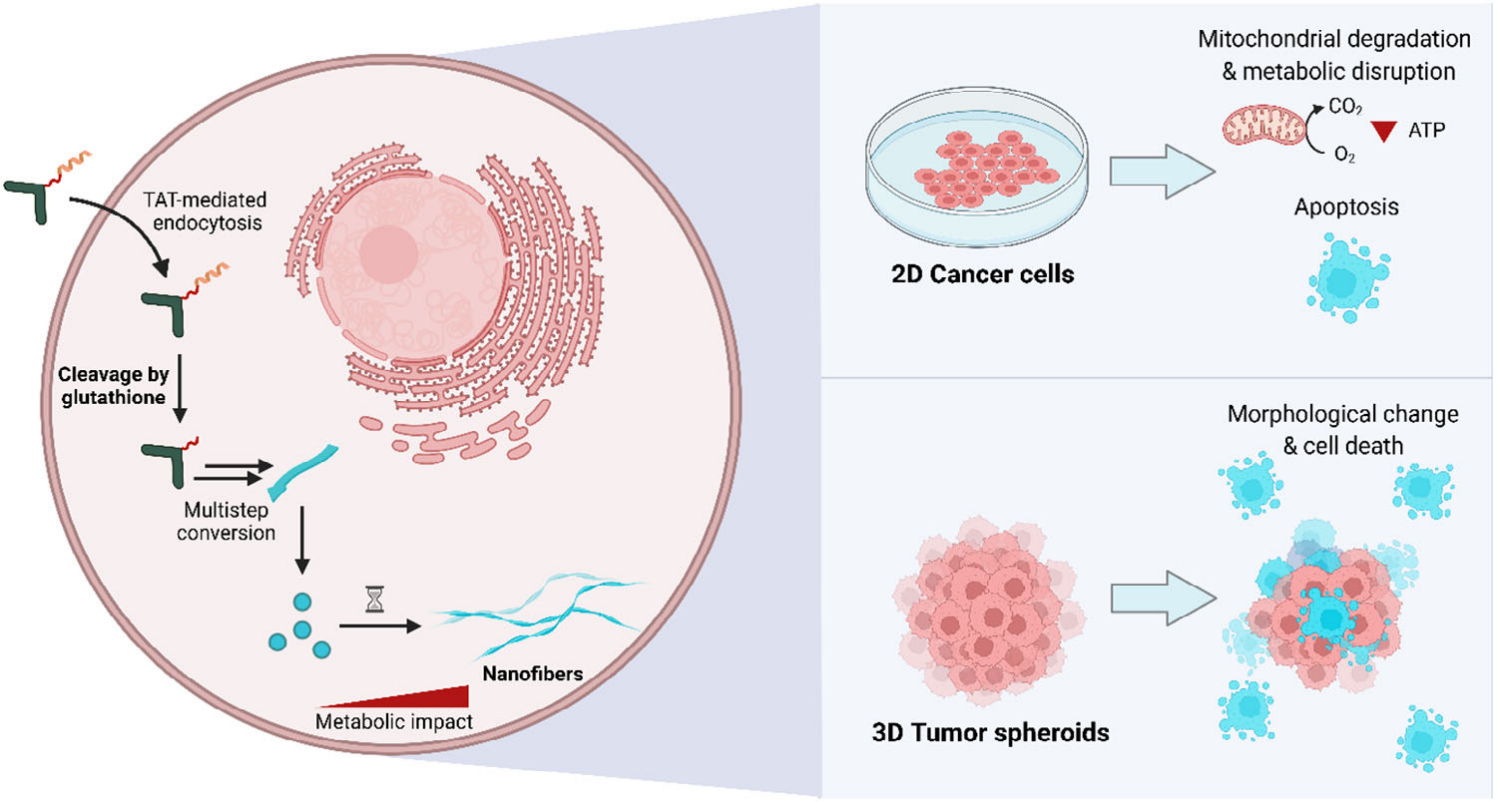
Schematic representation of glutathione‐induced intracellular peptide assembly inside 2D‐cultured cancer cells and 3D tumor spheroids. Glutathione‐responsive *iso*peptides enter the cells via transactivator of transcription (TAT)‐mediated endocytosis and are subjected to glutathione‐induced cleavage in the cytosol, followed by a multistep conversion into linear self‐assembling peptides. This intracellular transformation leads to the formation of nanostructures that disrupt the mitochondrial network causing cancer cell death and size decrease of tumor spheroids.

Herein, we present the in situ formation of metabolism‐disrupting nanostructures inside 2D‐cultured cancer cells and 3D tumor spheroids using glutathione‐responsive pro‐assembling *iso*peptides. These peptides undergo a multistep reaction cascade into self‐assembling linear peptides upon cellular uptake (**Figure** **1**). This study introduces live‐cell and fluorescent lifetime imaging to observe real‐time changes in the mitochondrial network and integrity during the formation of fluorescent peptide nanostructures for the first time. With similar time resolution, the impact on mitochondrial respiration is quantified by measuring the cellular oxygen consumption rate (OCR) via an extracellular flux analyzer. These measurements allow us to discern the effect of intracellular structure formation on the oxidative phosphorylation and energy homeostasis of multidrug‐resistant MDA‐MB‐231 triple‐negative breast cancer cells. Additionally, we investigate the toxicity of the glutathione‐responsive *iso*peptides in 3D MDA‐MB‐231 tumor spheroids, monitoring their size reduction to assess the biological impact of the in situ formed nanostructures.

## 2 Results and Discussion

### 2.1 Design and transformation of Glutathione‐Responsive *iso*peptides

**Figure 2:**
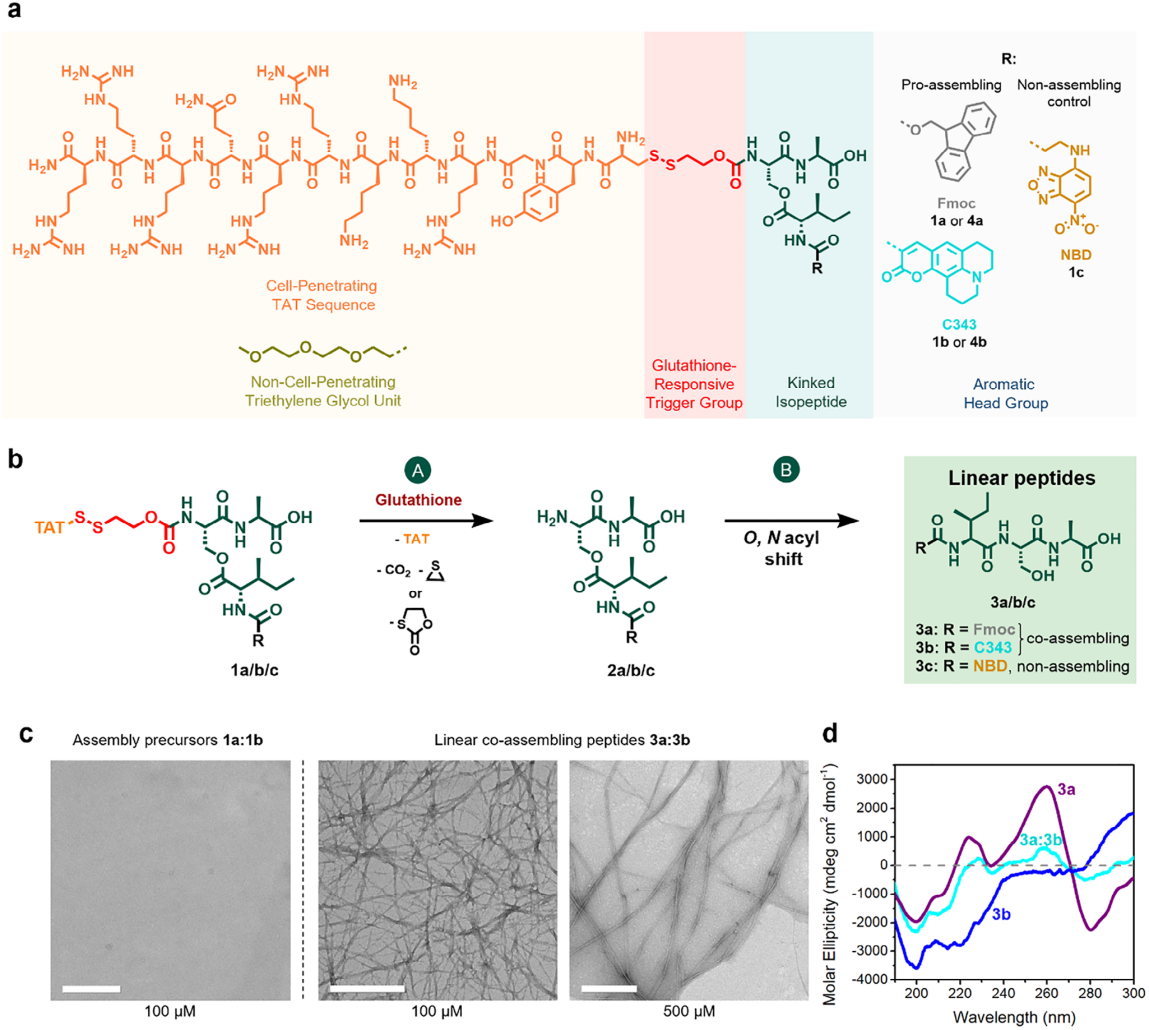
Chemical structures and multistep conversion of glutathione‐responsive *iso*peptides into fibrillating peptide nanostructures. a) Chemical structure of glutathione‐responsive pro‐assembling *iso*peptides **1a** (with a Fmoc (9‐fluorenylmethoxycarbonyl) group) and **1b** (with a C343 (Coumarin 343) moiety), as well as chemical structures of non‐assembling control *iso*peptides **1c** (with an NBD (nitrobenzodiazole) unit), and triethylene glycol‐modified non‐cell‐penetrating *iso*peptides **4a** and **4b**. b) Reaction scheme of the multistep conversion of glutathione‐responsive *iso*peptides **1a/b/c** via the deprotected *iso*peptides **2a/b/c** into the co‐assembling linear peptide monomers **3a** and **3b** or the non‐assembling linear peptide **3c**. c) Transmission electron micrographs (TEM) of nanofibers formed by co‐assembling peptides **3a** and **3b** (5:1 ratio at 500 and 100 µm) and TEM images of non‐assembling assembly precursors **1a** and **1b** (5:1 ratio at 100 µm) in DPBS (pH 7.4) and DMSO (99:1). Scale bar 500 nm. d) Circular dichroism (CD) spectra of linear peptide **3a** (purple curve), linear peptide **3b** (blue curve), and linear co‐assembling peptides **3a** and **3b** (5:1 ratio) (cyan curve) at 500 µm in phosphate buffer (10 mm, pH 7.4).

The glutathione‐responsive pro‐assembling *iso*peptides **1a** and **1b** consist of three main structural components: 1) the cell‐penetrating peptide sequence TAT (transactivator of transcription) derived from human immunodeficiency virus (HIV); 2) a glutathione‐responsive trigger group capable of reductive degradation; and 3) the pro‐assembling *iso*peptide with an aromatic *N‐*terminal group that is necessary for self‐assembly (Fmoc) and fluorescence (Coumarin 343), respectively (**Figure** **2**a).^[^ ^16^ ^]^ To assess the effects of glutathione‐induced transformation without self‐assembly, we synthesized the control *iso*peptide **1c** (Figure 2a). This control includes a nitrobenzodiazole (NBD) fluorophore at the *N*‐terminus and is designed to enter cells and undergo glutathione‐induced transformation but does not produce self‐assembling peptide monomers (Figure S21, Supporting Information). Additionally, non‐cell‐penetrating control *iso*peptides **4a** (with Fmoc) and **4b** (with Coumarin 343) were synthesized with a triethylene glycol unit replacing the cell‐penetrating TAT sequence (Figure 2a).

To investigate the structure formation of the linear co‐assembling peptides **3a** and **3b**, which are the final products of the glutathione‐induced transformation of **1a** and **1b**, we used dry‐state transmission electron microscopy (TEM) (Figure 2c; Figure S21, Supporting Information). The TEM images revealed that the co‐assembly of Fmoc‐ISA **3a** and Coumarin 343‐ISA **3b** formed elongated fibrillar peptide nanostructures at concentrations of 100 and 500 µm in a 5:1 ratio of **3a:3b** in DPBS (pH 7.4) with 1% DMSO. In contrast, the kinked pro‐assembling *iso*peptides **1a** and **1b** did not form such supramolecular structures under similar conditions, underscoring their role as assembly precursors that require a stimulus to transform into the self‐assembling monomers.

The circular dichroism (CD) spectra of the self‐assembling monomer Fmoc‐ISA **3a** exhibited nanoscale chirality^[^ ^18^ ^]^ featuring a peak at 260 nm indicative of π → π* transitions in the aromatic head groups (see Figure 2d). Additionally, a secondary peak at 224 nm was observed, corresponding to the n → π* transition of the carbonyl groups in the peptide backbone, which can be attributed to hydrogen bonding.^[^ ^19^, ^20^ ^]^ In contrast, the spectrum of the Coumarin 343‐functionalized linear peptide **3b** lacks the 260 nm signal entirely, confirming the absence of Fmoc‐Fmoc interactions. The signal indicative of backbone hydrogen bonding at 224 nm is present in the CD spectrum of **3b** but less pronounced compared to **3a**, suggesting weaker peptide backbone interactions. Notably, the CD spectrum of the co‐assembled **3a:3b** mixture showed a slightly lower intensity at 260 nm compared to the spectrum of the self‐assembling peptide **3a**. This reduction can be explained by the fact that the aromatic interactions between the Fmoc‐ and Coumarin 343‐functionalized peptides **3a** and **3b** are weaker than the interactions solely between the Fmoc groups in peptide **3a**.

To complement these structural analyses and to assess the critical aggregation concentration (CAC) of the co‐assembling peptides **3a** and **3b** (5:1 ratio), we conducted a Proteostat aggregation assay. This assay detects assemblies through an increase in fluorescence upon binding the Proteostat dye. The analysis indicated a CAC of 9.2 µM for the linear peptides **3a** and **3b** in a 5:1 ratio in DPBS (pH 7.4) with 1% DMSO (Figure S23, Supporting Information).

### 2.2 In Situ Formation of Peptide Nanostructures Inside Cancer Cells

**Figure 3:**
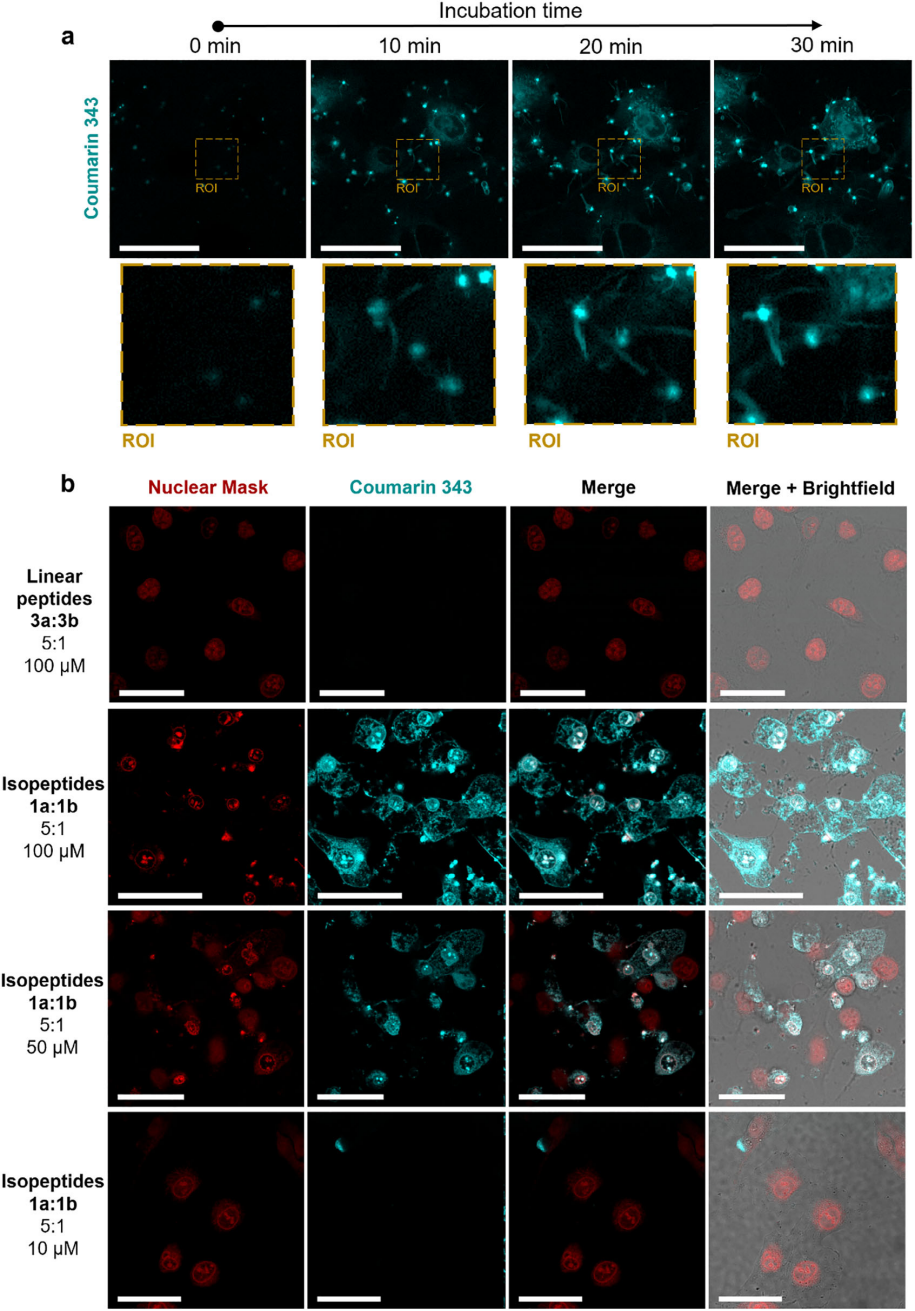
Cellular uptake and intracellular peptide assembly in cancer cells. a) Time‐dependent live‐cell imaging of MDA‐MB‐231 cells treated with 50 µm of the glutathione‐responsive *iso*peptides **1a** and **1b** (5:1) (cyan). The rectangle indicates a region of interest (ROI), in which structure transformation from globular peptide structures to fibrous nanostructures can be observed. Scale bars 50 µm. b) Confocal laser scanning microscopy (CLSM) images of MDA‐MB‐231 breast cancer cells after 4 h of incubation with glutathione‐responsive *iso*peptides **1a** and **1b** (5:1) at 100, 50, and 10 µm (cyan) (second to fourth row). As a control, cells were incubated with the linear peptides **3a** and **3b** (top row) at 100 µm. The nucleus was stained with HCS Nuclear Mask Deep Red (red). Scale bars 50 µm.

Next, we investigated the cellular uptake of the glutathione‐responsive *iso*peptides, their subcellular distribution, and the in situ formation of intracellular nanofibers. For this purpose, we incubated MDA‐MB‐231 breast cancer cells with a 5:1 mixture of Fmoc‐substituted *iso*peptide **1a** and Coumarin 343‐substituted *iso*peptide **1b** and examined the emergence of fluorescent intracellular nanostructures via confocal laser scanning microscopy (CLSM) (**Figure** **3**).

Time‐lapsed live‐cell imaging revealed that the TAT‐modified *iso*peptides **1a** and **1b** (5:1 ratio, 50 µm total peptide concentration) rapidly entered the cells and formed fluorescent peptide aggregates already within the first 15 min of incubation (Figure 3a; Video S1, Supporting Information). First, upon cell entry, we observed Coumarin 343‐associated fluorescence in localized round areas distributed throughout the cytosol (Figure 3a). After 10 min of incubation with **1a** and **1b**, we noticed fibrillar and directed peptide nanostructures growing in an aster‐like pattern from the round areas of increased fluorescence extending into the surrounding cellular space (Figure 3a, ROI). The cells exhibiting these intracellular structures showed a decrease in overall size and change in shape after 15 min (Figure 3a).

Further analysis of MDA‐MB‐231 breast cancer cells treated with the assembly precursors **1a:1b** at 100 and 50 µm for 4 h revealed the presence of highly fluorescent structures throughout the cytosol (Figure 3b, second and third row). Co‐staining of the nucleus showed a strong Coumarin 343‐fluorescence signal in the perinuclear and nuclear regions (Figure 3b, second and third row). This observation of co‐localization could be explained by the inherent nucleus‐targeting properties of TAT^[^ ^24^ ^]^ which may result in residual non‐cleaved assembly precursors accumulating in the proximity of the nucleus. Additionally, we observed that in the cells treated with **1a:1b** at 100 µm, the nucleus stain was most intense in small, condensed areas of the nuclear region (Figure 3b, second row). These observations are indicative of nuclear fragmentation, which is associated with decreased cell viability (Figure S35, Supporting Information). For the lower *iso*peptide concentration of 10 µm, the peptides remained contained within small areas for 4 h (Figure 3b, bottom row), and no significant disruption of cellular integrity was evident.

The formation of intracellular peptide architectures was further investigated using correlative light and electron microscopy (CLEM) (Figure S28, Supporting Information). CLEM analysis of MDA‐MB‐231 cells treated with 100 µm of **1a:1b** for 4 h revealed fluorescent peptide assemblies within the cytosol, especially in the nuclear and perinuclear regions (Figure S28, Supporting Information). In close proximity to fluorescent peptide nanostructures, mitochondria with disrupted membranes were observed, indicating that cytosolic assembly has negative impacts on the integrity of these organelles (Figure S28, Supporting Information). The subcellular distribution observed via CLEM was consistent with CLSM analysis (Figure 3; Video S1, Supporting Information), confirming the ability of *iso*peptides **1a:1b** to form nanostructures within living cells.

As a control, cells treated directly with the linear co‐assembling peptides **3a:3b** (5:1 ratio), the final products of the glutathione‐induced reaction cascade of **1a:1b**, did not show any uptake or impact on cellular morphology, underlining the importance of the TAT‐mediated cell entry and the in situ intracellular transformation (Figure 3b, top row). For the non‐assembly‐inducing control *iso*peptide **1c**, internalization was observed but without impact on cellular integrity (Figure S25, Supporting Information). This further supports that the in situ formation of supramolecular peptide nanostructures is necessary to elicit a biological response.

### 2.3 Peptide Nanostructures Disrupt the Cytoskeleton of Cancer Cells

**Figure 4:**
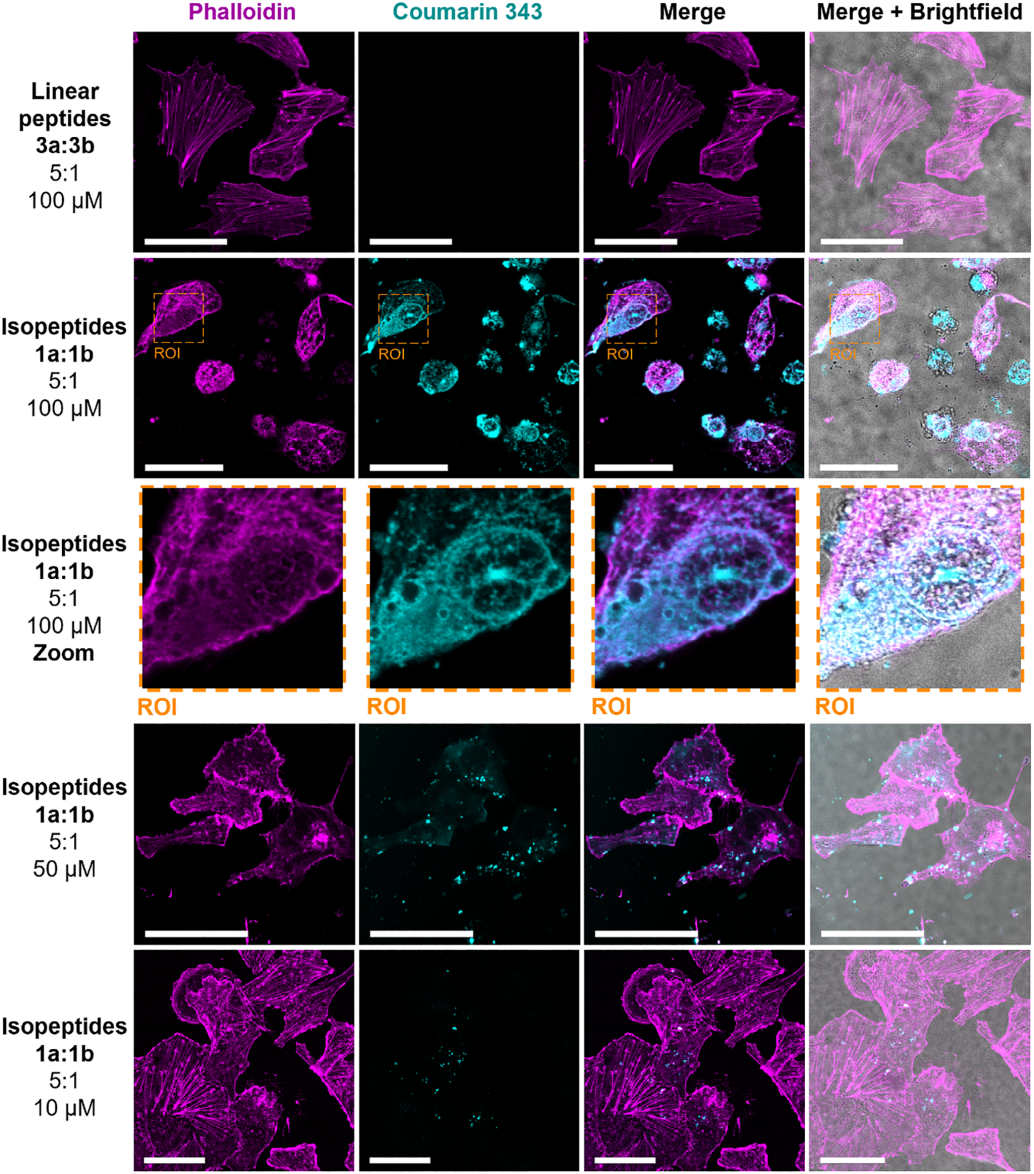
Disruption of actin filaments by in situ formed nanostructures. Confocal laser scanning microscopy (CLSM) images of MDA‐MB‐231 cells after 4 h of incubation with glutathione‐responsive *iso*peptides **1a** and **1b** (5:1) (cyan) at different concentrations or the linear peptides **3a:3b** and additional staining with Alexa Fluor 555 Phalloidin (pink). Scale bars 50 µm.

The cytoskeleton, particularly the actin filament network, plays a vital role in maintaining cell structure, movement, and organization.^[^ ^1^ ^]^ As we observed rapid morphological changes in cells in response to the formation of the peptide nanostructures, we seek to understand the relationship between the formation of synthetic peptide nanostructures and the loss of cytoskeletal integrity. Therefore, MDA‐MB‐231 cells were treated with varying concentrations of the *iso*peptides **1a:1b** for 4 h, and their actin filaments were subsequently stained with Alexa Fluor 555 Phalloidin (**Figure** **4**). At high concentrations of 100 or 50 µm of **1a:1b**, significant fragmentation of the actin filaments was observed (Figure 4, second to fourth row; Figure S25, Supporting Information). Notably, in areas of very high Coumarin 343 fluorescence, there was a marked absence of phalloidin‐stained actin filaments, suggesting that peptide nanostructure formation disrupts the cytoskeleton (Figure 4, second and third row, ROI). In cells incubated with 10 µm of the *iso*peptide mixture **1a:1b** (Figure 4, bottom row), no apparent changes to the integrity and density of the actin filaments were observed compared to the control cells treated with the linear peptides **3a:3b** (Figure 4, top row). To assess the time‐dependent effects on cytoskeletal integrity, we performed phalloidin staining on cells treated with the assembly‐inducing *iso*peptides **1a:1b** (at 100 and 10 µm) and the linear peptides **3a:3b** (at 100 µm) as a control. Imaging at 1, 2, 4, and 24 h treated with *iso*peptides **1a:1b** at 100 µm revealed that structural disruption of the cytoskeleton occurs rapidly, with pronounced effects already evident within 1 h of incubation (Figures S31–S33, Supporting Information).

To quantify the degree of actin cytoskeletal disruption, we conducted a western blot analysis of MDA‐MB‐231 cell lysates treated with either *iso*peptides **1a:1b** or linear control peptides **3a:3b** at 50 µm, comparing the G‐actin/F‐actin ratio after a 2 h incubation. G‐actin (globular actin) represents the unpolymerized, soluble form of actin, while F‐actin (filamentous actin) is the polymerized, structural form that contributes to the stability of the cytoskeleton.^[^ ^21^ ^]^ Cells treated with **1a:1b** (50 µm) exhibited a G‐actin/F‐actin ratio of 80.9% to 19.1%, whereas cells treated with **3a:3b** (50 µm) or the untreated control showed ratios of 62.9% to 37.1% and 62.2% to 37.8%, respectively (Figure S34, Supporting Information). This indicates that treatment with *iso*peptides causing intracellular structure formation leads to a measurable increase in the proportion of G‐actin relative to F‐actin, further suggesting cytoskeletal disruption.

### 2.4 Intracellular Structure Formation Impacts Metabolism, Disrupts Mitochondrial Integrity, and Causes Apoptosis

**Figure 5:**
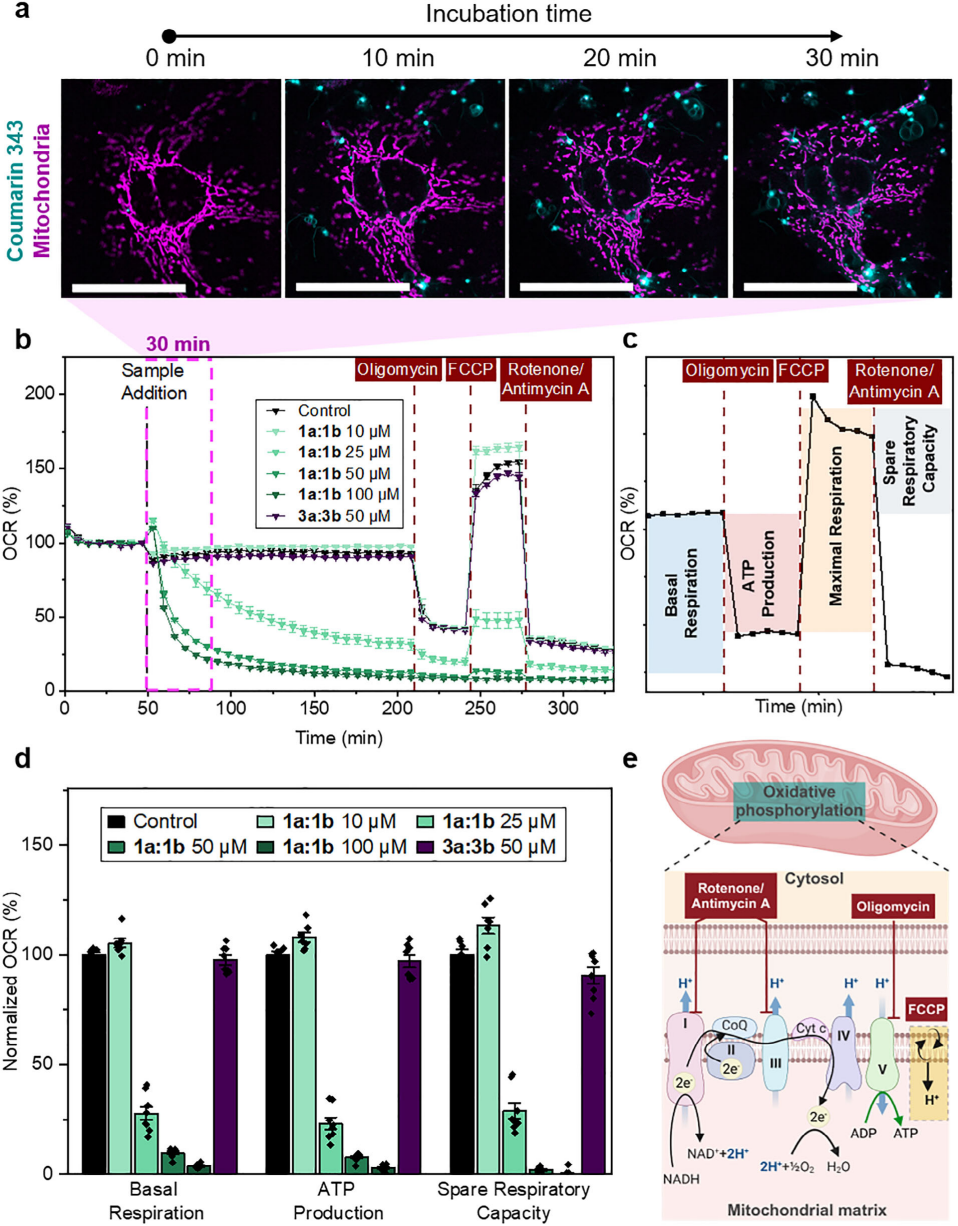
Effect of glutathione‐responsive *iso*peptides on mitochondrial integrity and respiration. a) CLSM images of live‐cell imaging of MDA‐MB‐231 cells treated with 50 µm of **1a:1b** (cyan). Cells were previously transfected with CellLight Mitochondria‐RFP (pink). Scale bars 50 µm. b) Effect of *iso*peptide mixture **1a:1b** and of control compounds on the oxygen consumption rate (OCR) of MDA‐MB‐231 cells during the Mito Stress Test Assay. The last measurement before treatment injection of the compound is set as 100%. *n* = 7 c) Representative OCR trace from the Mito Stress Test. Specific electron transport chain (ETC) modulators are added at designated time points to assess mitochondrial function: 1) oligomycin inhibits ATP synthase, 2) FCCP (carbonyl cyanide 4‐(trifluoromethoxy)phenylhydrazone) disrupts the mitochondrial membrane potential, 3) rotenone inhibits complex I and antimycin A inhibits complex III of the ETC.^[^ ^27^ ^]^ d) Effect of *iso*peptide mixture **1a:1b** and of control compounds on basal respiration, ATP production, and spare respiratory capacity. Values are normalized toward the untreated control group. Data are presented as mean ± s.e.m., n ≥ 7. Statistical significance was calculated by ANOVA with a Tukey post hoc test. ^*^ *p* < 0.05, ^**^ *p* < 0.01, ^***^ *p* < 0.001. e) Schematic representation of oxidative phosphorylation and the electron transport chain (ETC) in mitochondria, highlighting the effects of specific ETC modulators (oligomycin, FCCP, rotenone/antimycin A).

Analyzing the synthetic structure formation inside cells via CLSM and CLEM revealed morphological changes upon intracellular peptide assembly, which include blebbing, cell rounding and fragmentation of both nuclei and actin filaments (Figures 3 and 4; Video S1, Supporting Information). These findings indicate an effect on cell viability, which was determined in the next step using CellTiter‐Glo Assay after a 4 h incubation with the pro‐assembling precursors **1a:1b** at different concentrations. A concentration dependent effect on cell viability was observed and an IC_50_ of 36.4 ± 3.1 µm was calculated (Figure S35, Supporting Information). In combination with the CLEM images, which revealed a localization of peptide nanofibers in close proximity to mitochondria with disrupted membranes, these results indicate that the peptide nanofibers impact energy metabolism of the cells. To gain further insights into the mechanism of how synthetic peptide nanostructures effect cell integrity, mitochondria function was investigated in more detail, as these organelles are essential for cell metabolism and dynamics, and maintaining energy homeostasis. To assess the impact of peptide assembly on mitochondrial structure and function, we performed live‐cell imaging with cells that had been transfected with CellLight Mitochondria‐RFP (**Figure** **5**a; Figure S26 and Video S2, Supporting Information). We observed that within the first 30 min of incubation with 50 µm of the *iso*peptides **1a:1b** the mitochondria of the cancer cells underwent significant changes: starting from an interconnected network of tubular‐shaped organelles, their morphology transformed into shrunken, fragmented, and more punctiform mitochondria (Figure 5a; Video S2, Supporting Information). These observations point toward mitochondrial fragmentation, which could be a result of changes in the rates of mitochondrial fusion and fission events^[^ ^25^ ^]^ indicative of early stages of cell death.^26^

To further investigate the mitochondrial disruption, we quantitatively assessed various aspect of mitochondrial respiration as a function of the formation of the peptide nanostructures. Using a Seahorse XFe96 extracellular flux analyzer, we measured the oxygen consumption rate (OCR) of cells treated with various electron transport chain (ETC) modulators, providing further insights into cellular respiration (Figure 5c,e).^[^ ^22^ ^]^ When adding the pro‐assembling *iso*peptides **1a:1b** to the cells, we observed a concentration‐dependent decrease in OCR already within the first 30 min of incubation (Figure 5b). For the lowest concentration of 10 µm, no effect on the OCR was found indicating cellular tolerance to this amount of assembly precursors. This correlates with the cell viability data, where no negative effect was observed at this concentration (Figure S35, Supporting Information).

However, starting at 25 µm total *iso*peptide concentration, a rapid reduction in OCR was observed, which was even more pronounced at 50 and 100 µm (Figure 5b). This showed that the addition of the assembly precursors **1a:1b** at concentrations capable of causing intracellular structure formation led to a significant decrease in basal respiration within <30 min. These quantitative findings aligned with the visual data from live‐cell imaging of mitochondria‐stained cells (Figure 5a), confirming mitochondrial dysfunction as a consequence of intracellular peptide assembly. Besides examining the cellular response to **1a:1b** in terms of basal respiration, we also studied the impact on ATP production and the spare respiratory capacity, as both properties are indicative of mitochondrial health and metabolic function (Figure 5d). Here we also noticed a concentration‐dependent effect of the *iso*peptides **1a:1b** with cells being treated with ≥25 µm overall peptide concentration exhibiting a significantly reduced ATP production and spare respiratory capacity (Figure 5d). In contrast, no effect on mitochondrial function was observed for the linear peptides **3a:3b**, underscoring the importance of intracellular accumulation of assembly precursors and the propagation of resulting peptide assemblies to elicit a biological response.

To expand our analysis, we also investigated the effects of treatment with *iso*peptides **1a:1b** on A549 cells, a non‐small cell lung cancer (NSCLC) line, on cell viability and mitochondrial respiration. Treatment with **1a:1b** for 4 h resulted in an IC_50_ value of 81.7 ± 7.1 µm for A549 cells (Figure S36, Supporting Information), showing comparable but slightly lower toxicity than in MDA‐MB‐231 cells (IC_50_: 36.4 ± 3.1 µM, Figure S35, Supporting Information). Seahorse metabolic analysis of A549 cells revealed that incubation with ≥25 µm of **1a:1b** caused a rapid decrease in basal respiration and disruption of mitochondrial functions (Figure S43, Supporting Information), similar to effects observed in MDA‐MB‐231 cells (Figure 5b). These findings suggest that the toxicity from intracellular structure formation is evident in both cancer cell lines.

**Figure 6:**
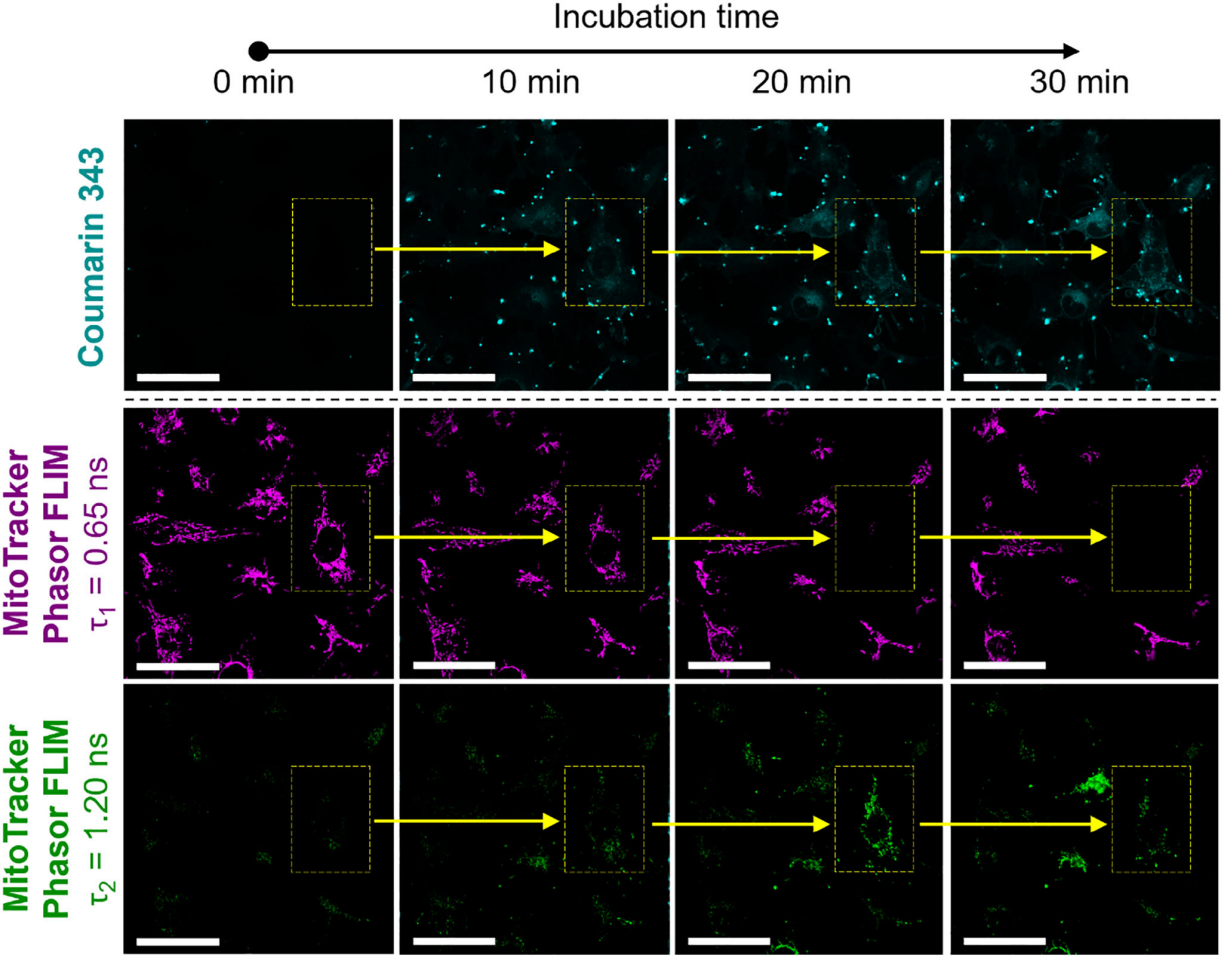
Changes in fluorescence lifetime of mitochondrial stain following intracellular structure formation. CLSM images show live‐cell imaging of MDA‐MB‐231 cells treated with 50 µm of **1a:1b** (peptide fluorescence shown in cyan). Cells were previously stained with MitoTracker DeepRed. Fluorescence lifetime measurements of the stain were conducted concurrently with fluorescence imaging, revealing two distinct fluorescence lifetime signals for MitoTracker DeepRed over time. The corresponding phasor plots are provided in the Supporting Information (Figure S27, Supporting Information). Scale bars 50 µm.

In addition to examining mitochondrial fragmentation via genetically encoded fluorescence labeling of the organelle, MitoTracker DeepRed was used as a co‐staining reagent (**Figure** **6**; Video S3, Supporting Information). Unexpectedly, we observed that the dye underwent a change in fluorescence lifetime (FL) within cells exposed to the pro‐assembling *iso*peptides **1a:1b** during live‐cell imaging. Initially, the FL signal of the stain was uniform with signal component decay at 0.65 ns and could be observed homogeneously in all examined cells. However, the addition of the *iso*peptides **1a:1b** at 50 µm caused a shift in the FL of MitoTracker DeepRed to 1.20 ns, particularly in those cells exhibiting high Coumarin 343 fluorescence of the peptide nanostructures (Figure 6; Video S4, Supporting Information). The observed shift in the photochemical properties of the dye is likely caused by changes in its chemical environment, which appear to correlate with the degradation of the mitochondrial network as a result of the intracellular formation of peptide nanostructures.

To investigate the activation of apoptosis‐related enzymes in relation to intracellular structure formation, we conducted a quantitative analysis of caspase‐3/7 activation in cells treated with *iso*peptides **1a:1b** as well as live cell imaging using a caspase‐3/7‐sensitive fluorogenic dye. Caspase‐3 and ‐7 are key executioner caspases in apoptosis, responsible for cleaving structural and regulatory proteins to facilitate controlled cellular disassembly.^[^ ^23^ ^]^ Therefore, their activation serves as a marker of apoptosis, linking upstream signaling events to the morphological and biochemical changes characteristic of programmed cell death.^[^ ^24^ ^]^

**Figure 7:**
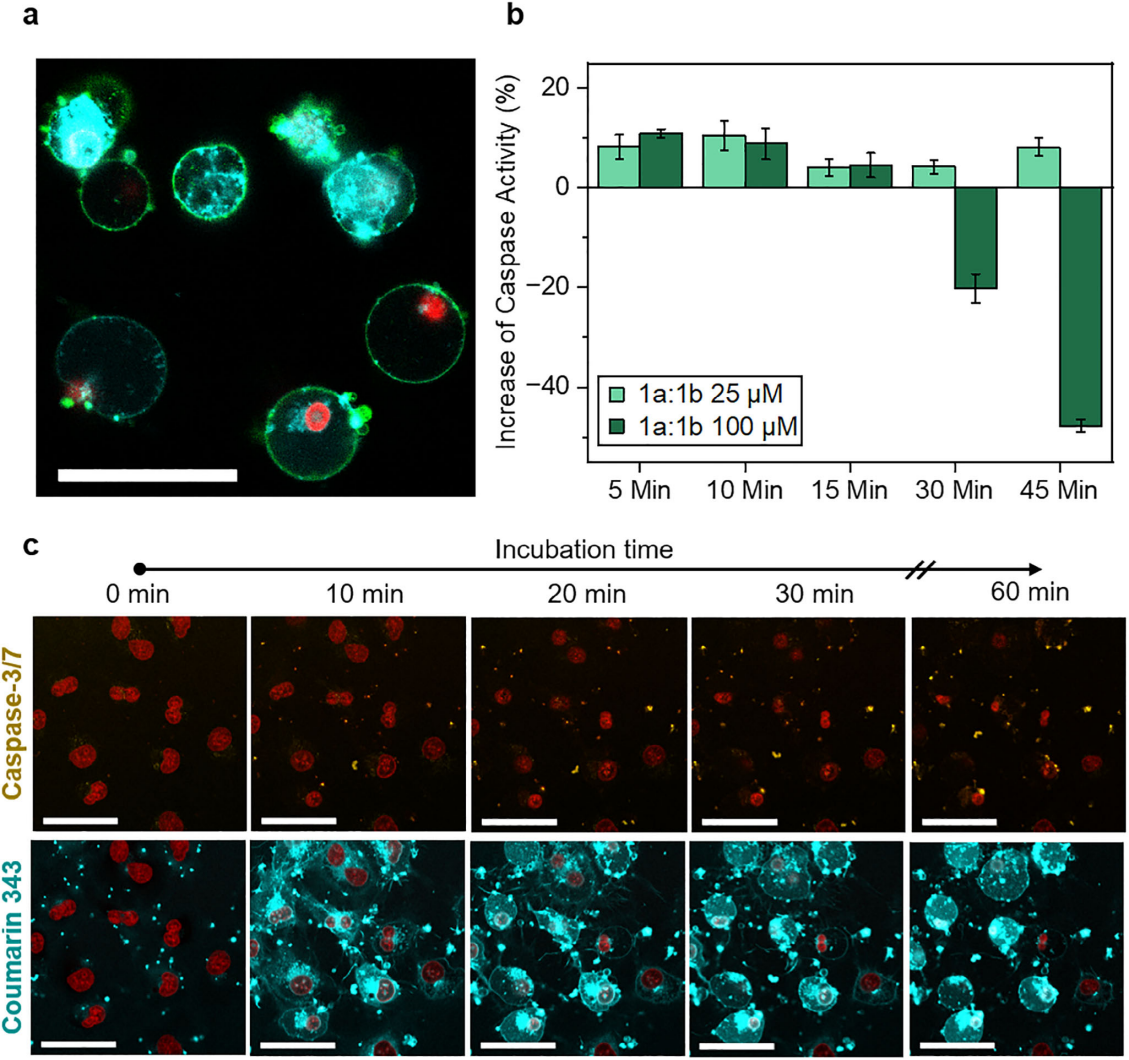
Apoptosis induced by intracellular peptide structure formation. a) Confocal laser scanning microscopy (CLSM) image of MDA‐MB‐231 cell after 1 h of incubation with *iso*peptides **1a** and **1b** (5:1) (cyan) at 50 µm and additional staining with Annexin V (green) and propidium iodide (red). Scale bars 50 µm. b) Caspase‐Glo Assay results showing caspase activation in MDA‐MB‐231 cells after incubation with *iso*peptides **1a:1b** at 25 or 100 µm at different timepoints. Increased caspase‐3/7 activity indicates increased apoptosis compared to untreated control cells. Data represent mean ± s.e.m. (n ≥3). c) Live cell imaging of MDA‐MB‐231 cells treated with *iso*peptides **1a**:**1b** (5:1) (cyan) at 50 µm. Cells were pre‐stained with Caspase‐3/7 sensitive fluorogenic dye (yellow) and Nuclear Deep Red Mask (red). Scale bars 50 µm.

Live‐cell imaging of MDA‐MB‐231 cells treated with 50 µm *iso*peptides **1a:1b** revealed caspase‐3/7 activation coinciding with the formation of fluorescent intracellular peptide structures. This activation was detectable within 10 min, as evidenced by the appearance of fluorescent areas in the cytosol resulting from the cleavage of the fluorogenic caspase‐3/7‐sensitive dye (**Figure** **7**c, upper row). The caspase‐3/7‐induced fluorescence signal increased over time, up to 60 min of incubation, indicating accumulation of the fluorescent caspase cleavage product. The quantitative caspase‐3/7 assay further confirmed this rapid activation, with enzyme activity rising within the first 5 to 10 min of *iso*peptide treatment (Figure 7b). Notably, at a high concentration of 100 µm of **1a:1b**, caspase‐3/7 activity declined again after 30 min, likely due to excessive toxicity (Figure S35, Supporting Information) and subsequent fast cellular degradation. In contrast, treatment with linear peptides **3a:3b** showed no measurable effect on caspase‐3/7 activity (Figure S38, Supporting Information).

To further characterize apoptotic progression, we also performed Annexin V staining, which detects phosphatidylserine externalization, an early apoptosis marker resulting from impaired flippase activity.^[^ ^25^ ^]^ Cells treated with 50 µm of **1a:1b** for 1 h exhibited Annexin V staining at the plasma membrane, confirming apoptotic induction (Figure 7a).

Together, these complementary analyses of caspase‐3/7 activity and Annexin V staining provide strong evidence that apoptosis is a key mechanism underlying the cytotoxic effects of **1a:1b**.

### 2.5 Intracellular Peptide Nanostructures have a Toxic Effect on Tumor Spheroids and Cause Size Reduction

**Figure 8:**
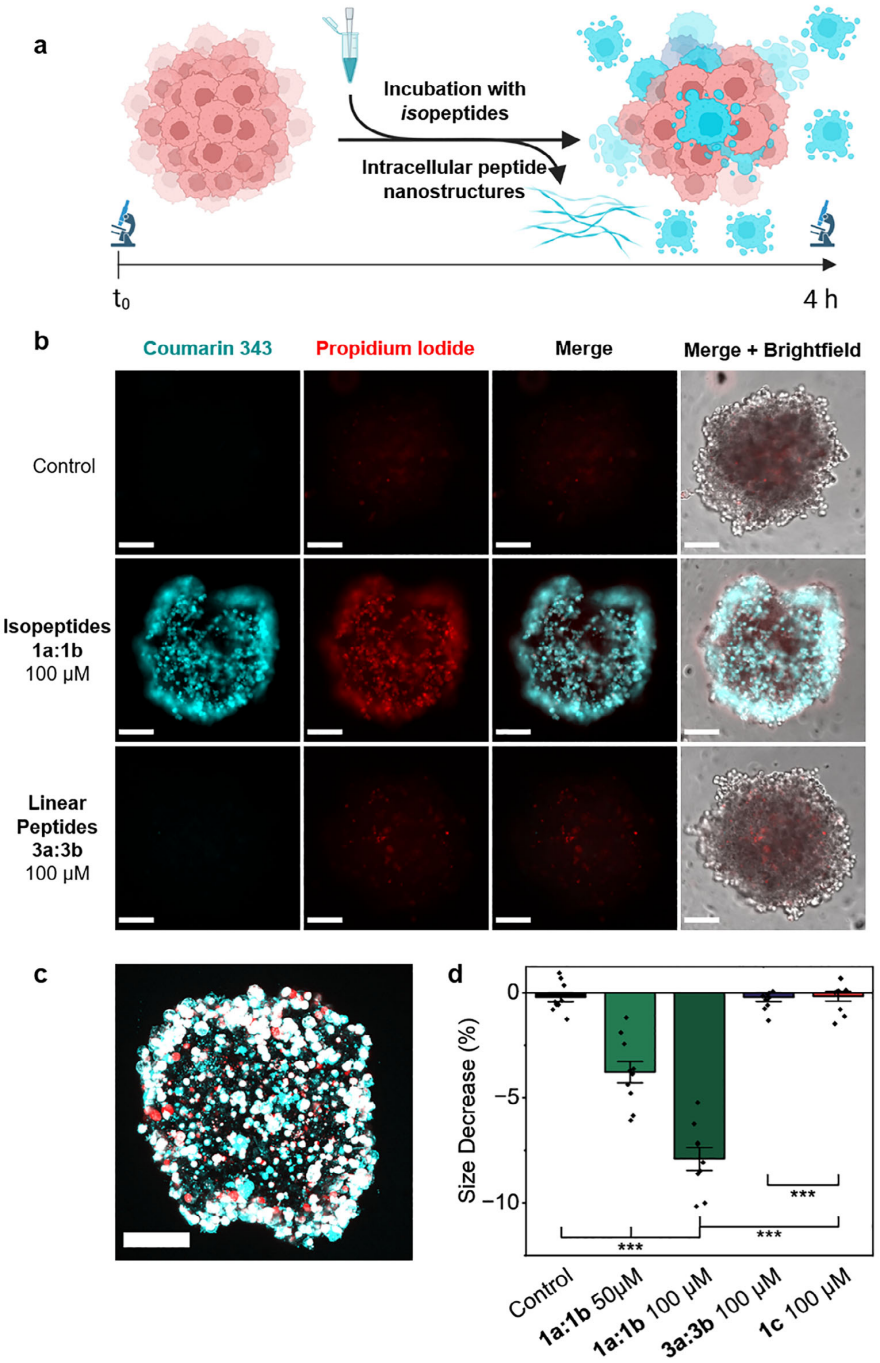
Intracellular peptide assembly disrupts tumor spheroid integrity. a) Schematic representation of tumor spheroid treatment with glutathione‐responsive *iso*peptides and subsequent toxic intracellular assembly formation. b) Fluorescence microscopy images of uptake and dead staining of tumor spheroids. Coumarin 343 fluorescence is shown in cyan, and propidium iodide‐stained dead cells are red. Scale bars 100 µm. c) Maximum projection of a z‐stack of a spheroid incubated with **1a:1b** at 100 µm for 4 h. d) Spheroid size was measured before and after incubation with glutathione‐responsive *iso*peptides using brightfield images in ImageJ. Diameter of each spheroid was measured at four different places (Figure S46, Supporting Information). Data are presented as mean ± s.e.m., *n* = 10. Statistical significance was calculated by ANOVA with a Tukey post hoc test. ^*^ *p* < 0.05, ^**^ *p* < 0.01, ^***^ *p* < 0.001.

After observing the impact of intracellular assemblies on cellular organelles such as the cytoskeleton and mitochondria in 2D cell culture and their ability to induce apoptosis, we next investigated their effects on 3D cell networks. To assess the impact of intracellular peptide assembly on cell networks, we treated tumor spheroids derived from MDA‐MB‐231 breast cancer cells with glutathione‐sensitive assembly precursors **1a:1b** and monitored their uptake after 4 h with fluorescence microscopy. After the incubation with the peptide precursors, spheroids were stained with propidium iodide (PI), which is unable to pass the membrane of healthy cells, to further investigate the impact on cell viability (**Figure** **8**a,b).

Consistent with the 2D cell experiments, incubation with the **1a:1b** precursors at 100 µm resulted in a strong Coumarin 343 fluorescence signal within the spheroids (Figure 8b, second row). Cellular uptake was observed uniformly on the surface of the spheroids (Figure 8b,c). Additionally, slicing and subsequent imaging of tumor spheroids also revealed distribution of fluorescent peptides into the core of the spheroid, highlighting efficient penetration and intracellular localization throughout the 3D tumor spheroid (Figure S53, Supporting Information). In contrast, spheroids treated with the linear peptides **3a:3b** did not show any internalization, showing the necessity for the TAT‐facilitated cellular uptake and intracellular activation cascade to obtain intracellular structures that directly affect cell integrity (Figure S45, Supporting Information). Spheroids treated with the non‐assembling *iso*peptide **1c** showed a weak fluorescence signal, indicating uptake without subsequent assembly formation (Figure S45, Supporting Information).

In comparison to the control spheroids, those incubated with **1a:1b** at 50 or 100 µm exhibited a significant decrease in size after 4 h of incubation (Figure 8d; Figures S44–S46, Supporting Information). While spheroids incubated with **1a:1b** at 100 and 50 µm decreased in size by 7.9 ± 0.5% and 3.8 ± 0.5%, respectively, spheroids incubated with the linear peptides **3a:3b** or non‐assembling NBD‐labeled peptides **1c** did not show any size changes during the incubation period (Figure 8d). This suggests that the uptake of the assembly precursors into cells leads to cell death, resulting in a decrease in tumor spheroid size, whereas no effects on the spheroid stability were observed for the treatment with the linear or non‐assembling control peptides **3a:3b** and **1c** (Figure 8d). To further investigate the effects of prolonged *iso*peptide treatment on 3D tumor spheroids, we extended incubation to 24 and 48 h, assessing morphological changes and growth inhibition. Spheroid size further decreased by 13.9% at 24 h and 16.9% at 48 h after treatment with 100 µm of **1a:1b** (Figure S47, Supporting Information). Fluorescence imaging revealed progressive disintegration of outer cell layers (Figure S48, Supporting Information).

Dead staining with propidium iodide (PI) revealed a stronger fluorescence signal for spheroids incubated with the glutathione‐responsive *iso*peptides **1a:1b** at 100 µm for 4 h compared to the spheroids incubated with **1a:1b** at 50 µm or the spheroids incubated with **3a:3b** at 100 µm (Figure 8b). The residual fluorescence signal for propidium iodide for untreated and control spheroids is due to the natural necrotic core of the spheroids.^[^ ^17^ ^]^ The co‐localization of Coumarin 343 fluorescence and the PI stain for spheroids incubated with the *iso*peptides **1a:1b** at 100 µm confirms that uptake and intracellular transformation of the *iso*peptides causes cell death, thereby disrupting the overall spheroid integrity (Figure 8b,d).

Aside from tumor spheroids grown from MDA‐MB‐231 cells, we additionally investigated 3D A549 tumor spheroids. Treatment with **1a:1b** at 100 µm for 4, 24, and 48 h also demonstrated uptake of fluorescently labeled *iso*peptides and resulted in cytotoxicity, as shown by propidium iodide staining (Figure S52, Supporting Information). Spheroid slicing further confirmed successful *iso*peptide uptake and distribution of fluorescent peptides into the core of A549 tumor spheroids (Figure S54, Supporting Information).

## 3 Conclusion

In conclusion, using glutathione‐responsive *iso*peptides as assembly precursors has enabled us to assemble intracellular peptide nanostructures that disrupt the energy homeostasis of cancer cells and cause rapid cell death. The efficient cellular uptake and the fast glutathione‐induced transformation of the kinked *iso*peptides into the linear self‐assembling monomers allows the controlled formation of synthetic architectures within the complex cellular environment, both in 2D‐cultured cells and 3D tumor spheroids. These nanostructures effectively disrupt essential subcellular structures and processes, such as mitochondrial function and the actin cytoskeleton, which results in apoptotic cell death and a significant reduction in spheroid size. By combining qualitative time‐dependent microscopy with quantitative metabolic analysis of oxygen consumption rates and mitochondrial stress, we established a clear link between structural alterations in the mitochondrial network and the functional decline in mitochondrial respiration. Furthermore, using tumor spheroids as a model for 3D cellular networks enabled us to study the disruptive effects of intracellular synthetic nanostructures in a more complex and biologically relevant context. By continuing to explore the interactions between artificial and natural structures, we can gain important insights into the fundamental principles that govern cellular responses to synthetic assemblies.

## Conflict of Interest

The authors declare no conflict of interest.

## Author Contributions

S.C. and K.M. contributed equally to this work. S.C., D.Y.W.N., and T.W. conceived the research concept. S.C. conducted the synthesis and characterization of peptides and associated molecules. J.L. and J.F. aided in the synthesis of peptides. S.C. and K.M. performed cell uptake experiments and cell viability assays. K.M. conducted the metabolic analysis and the tumor spheroid experiments. M. J. S. A. S. and L.F. aided in the tumor spheroid experiments. P.R. performed the TEM measurements. S.C. and R.M. conducted the CD measurements. A.K., I.L., and K.L. contributed CLEM measurements and analysis. S.C. and M.W. performed the NMR analysis. D.Y.W.N. and T.W. supervised the project and acquired funding. All authors have read and approved the final manuscript. All the authors have read and commented on the manuscript.

## Supporting information



Supporting Information

Supplemental Video 1

Supplemental Video 2

Supplemental Video 3

Supplemental Video 4

## Data Availability

The data that support the findings of this study are available in the supplementary material of this article.
