# Supplementary material for: Intracellular Formation of Synthetic Peptide Nanostructures Causes Mitochondrial Disruption and Cell Death in Tumor Spheroids
Source: Adv Sci (Weinh). 2025 May 26;12(25):2412606. doi: 10.1002/advs.202412606 (PMC12224938; doi:10.1002/advs.202412606)
Supplement: Supplementary file 1 — Supporting Information [file ADVS-12-2412606-s004.pdf]

ADVANCED SCIENCE
Open Access

Supporting Information

for *Adv. Sci.*, DOI 10.1002/advs.202412606

Intracellular Formation of Synthetic Peptide Nanostructures Causes Mitochondrial Disruption and Cell Death in Tumor Spheroids

Sarah Chagri, Konrad Maxeiner, Maria J. S. A. Silva, Lisa Förch, Julian Link, Patrick Roth, Raphael Meyer, Jana Fetzer, Anke Kaltbeitzel, Ingo Lieberwirth, Katharina Landfester, Manfred Wagner, David Y.W. Ng and Tanja Weil**

Supporting Information

Sarah Chagri‡, Konrad Maxeiner‡, Maria J. S. A. Silva, Lisa Förch, Julian Link, Patrick Roth, Raphael Meyer, Jana Fetzer, Anke Kaltbeitzel, Ingo Lieberwirth, Katharina Landfester, Manfred Wagner, David Y.W. Ng, and Tanja Weil**

S. Chagri‡, K. Maxeiner‡, M. J. S. A. Silva, L. Förch, J. Link, P. Roth, R. Meyer, J. Fetzer, A. Kaltbeitzel, I. Lieberwirth, K. Landfester, M. Wagner, D.Y.W. Ng*, and T. Weil*

Max Planck Institute for Polymer Research, 55128 Mainz, Germany

Emails: david.ng@mpip-mainz.mpg.de; weil@mpip-mainz.mpg.de

Table of content

1. General Information 3
1.1. Materials 3
1.2. Instruments 3
1.2.1. Microwave Peptide Synthesizer 3
1.2.2. Liquid Chromatography-Mass Spectrometry (LC-MS) 3
1.2.3. Atmospheric-Pressure Chemical Ionization Mass Spectrometry (APCI-MS) 3
1.2.4. Nuclear Magnetic Resonance Spectroscopy (NMR) 3
1.2.5. Transmission Electron Microscopy (TEM) 3
1.2.6. 2D Cell Culture 4
1.2.7. Tumor Spheroid Culture 4
1.2.8. Fluorescence Microscopy 4
1.2.9. Confocal Laser Scanning Microscopy (CLSM) 4
2. Synthesis 6
2.1. Synthesis of glutathione-responsive isopeptides 6
2.1.1. Synthesis of isopeptides with activated disulfide bond 6
2.1.2. Solid phase supported synthesis of TAT-modified glutathione-responsive isopeptides 15
2.2. Synthesis of control compounds 18
2.2.1. Synthesis of triethylene glycol-modified control isopeptides 18
2.2.2. Analytical data of linear peptides 23
3. Analysis of secondary structure and assembly behavior 25
3.1. TEM analysis 25
3.2. Proteostat assay for the determination of the critical aggregation concentration 27
3.3. Stability analysis of isopeptide 1b in cell culture medium 27
4. Cell Experiments in 2D cell culture 29
4.1. Cell uptake analysis 29
4.2. Live-cell imaging 30
4.3 Correlative light- and electron microscopy (CLEM) of cells 32
4.4. Co-staining of actin filaments 34
4.5. Analysis of G-/F-actin ratio 37
4.6. Cell viability assay 40
4.7. Analysis of apoptosis 41
5. Analysis of Metabolic Disruption 43
5.1. Mito Stress test 43
6. Tumor Spheroid Experiments 49
6.1. Evaluation of uptake and toxicity on MDA-MB-231 spheroids 49
6.2. Slicing of spheroids 57

1. General Information

1.1. Materials

Reagents and solvents were purchased from commercial sources and were used without further purification. Peptide synthesis grade reagents were used for synthesizing the peptides. HPLC was performed using acetonitrile (CH_3CN) in HPLC grade (containing 0.1% trifluoroacetic acid (TFA)) and water for HPLC (containing 0.1% TFA) and reactions was obtained from a Millipore purification system. Flash column chromatography was carried out using Macherey-Nagel silica gel 0.04–0.063 mm.

1.2. Instruments

1.2.1. Microwave Peptide Synthesizer

Linear peptides were synthesized in a Liberty Blue Automated Microwave Peptide Synthesizer by CEM Corporation.

1.2.2. Liquid Chromatography-Mass Spectrometry (LC-MS)

Compounds were analyzed by HPLC-ESI-MS on a LC-MS 2020 by Shimadzu using a Kinetex 2.6 µm EVO C18 100 Å LC 50 × 2.1 mm column. MilliQ water acidified with 0.1% formic acid and CH_3CN were used as solvents for all measurements. The solvent gradient started with 5% CH_3CN and 95% water. This solvent ratio was kept constant for 2 min, then the CH_3CN content was linearly increased to 95% in 14 min. Data were processed in LabSolutions and OriginPro 9.

1.2.3. Atmospheric-Pressure Chemical Ionization Mass Spectrometry (APCI-MS)

All APCI-MS spectra were recorded on a Advion Expression-L Compact mass spectrometer. Data processing was performed in Advion Data Express.

1.2.4. Nuclear Magnetic Resonance Spectroscopy (NMR)

NMR spectra of small molecules and peptides were recorded on a Bruker Avance II 300 MHz spectrometer and a Bruker Avance 400 MHz spectrometer. The solvent signal was used as a reference (deuterated chloroform $CDCl_3$ δ =7.26 ppm for ^1H, 77.16 ppm for ^{13}C and for DMSO-d_6 2.50 ppm and 39.52 ppm respectively). The data was processed in MestReNova.

1.2.5. Transmission Electron Microscopy (TEM)

TEM images of the peptide samples were recorded on a JEOL 1400 transmission electron microscope at a voltage of 120 kV. Formvar/carbon-film coated copper grids (300 mesh) by Plano GmbH were used to prepare the samples. The images were processed in Fiji ImageJ or Icy.

1.2.6. 2D Cell Culture

MDA-MB-231 cells were cultured at 37 °C and 5% CO_2 in Dulbecco's Modified Eagle's Medium (DMEM, high glucose), supplemented with 10% fetal bovine serum (FBS). The cells were cultured in T75 culture flask and subcultivated two to three times per week.

1.2.7. Tumor Spheroid Culture

MDA-MB-231 cells were seeded in Ultra-Low-Attachment (ULA) 96-well plates (Corning, Ref. 4520) at a cell density of 10,000 cells/well. The plate was centrifuged (1,200 rpm, RT, 5 min) to allow formation of pre-clusters facilitating sphere development over time. Spheroids were cultured for 7 days in complete medium (DMEM high glucose with glutamine supplemented with 10 % FBS) at controlled humidified atmosphere (37 °C, 5% CO_2).

1.2.8. Fluorescence Microscopy

Fluorescence images of the tumor spheroids were taken using a Keyence BZ-X810 fluorescence microscope using a 20x LD PH lens. Propidium iodide staining for dead imaging of tumor spheroids was analyzed using Cy5 filter with the following wavelength settings: Ex. 470/40 nm, Em. 525/50 nm. For Coumarin 343 imaging of the peptide co-assemblies a GFP filter was used: Ex.: 470/40 nm, Em.: 525/50 nm.

1.2.9. Confocal Laser Scanning Microscopy (CLSM)

Cells were imaged live using an incubator-equipped Leica Stellaris® 8 microscope (40x/1.25 glycerol immersion objective) with fast lifetime contrast (FALCON) module (Leica Microsystems GmbH). The incubator (okolab) is set and held constant at 37 °C, 5% CO_2 and a relative humidity of 90% throughout all measurements. For the detection of Coumarin 343 fluorescence, samples were excited using a 40 MHz pulsed white light laser tuned to 440 nm. Emitted photons were detected using HyD® S2 (GaAsP hybrid photocathode) detector with a filter window at *475 nm – 550 nm*. The IRF standard used is fluorescein sodium salt.

Fluorescence lifetime imaging (FLIM) was performed on living cells at *2.5*-minute intervals, with a scanning resolution of 1024 x 1024 pixels at 200 Hz, accumulating *15* frames per acquisition. Adaptive focusing was applied to each frame and well position. A total of 100 frames were captured. Photon counting was conducted using a FALCON-modified time-correlated single photon counting (TCSPC) method.

Each pixel was converted into a phasor plot using the following equation:

$$g_{i,j}(\omega) = \int_0^T I(t) \cdot \cos(n\omega t)\mathrm{d}t / \int_0^T I(t)\mathrm{d}t$$

$$s_{i,j}(\omega) = \int_0^T I(t) \cdot \sin(n\omega t)\mathrm{d}t / \int_0^T I(t)\mathrm{d}t$$

In this equation, $g_{i,j}(\omega)$ and $s_{i,j}(\omega)$ represent the x and y coordinates of the phasor plot, n and ω are the harmonic and angular frequencies of excitation, respectively, and T is the acquisition's repeat frequency. The frequency domain data acquired from each pixel can be transformed into phasor points using the following transformations:

$$g_{i,j}(\omega) = m_{i,j} \cdot \cos\left(\phi_{i,j}\right)$$

$$s_{i,j}(\omega) = m_{i,j} \cdot \sin\left(\phi_{i,j}\right)$$

In these transformations, $m_{i,j}(\omega)$ and $\phi_{i,j}(\omega)$ represent the modulation and phase shift of the frequency domain measurement at pixel i,j. The decay at each pixel is thus mapped to a point in the phasor plot. Phasor components were identified and separated using LAS X software, and photons outside the defined phasor region were excluded from the images.

2. Synthesis

2.1. Synthesis of glutathione-responsive isopeptides

The synthesis of the glutathione-responsive isopeptides described in the following subsections have been reported previously by our group.[1]

2.1.1. Synthesis of isopeptides with activated disulfide bond

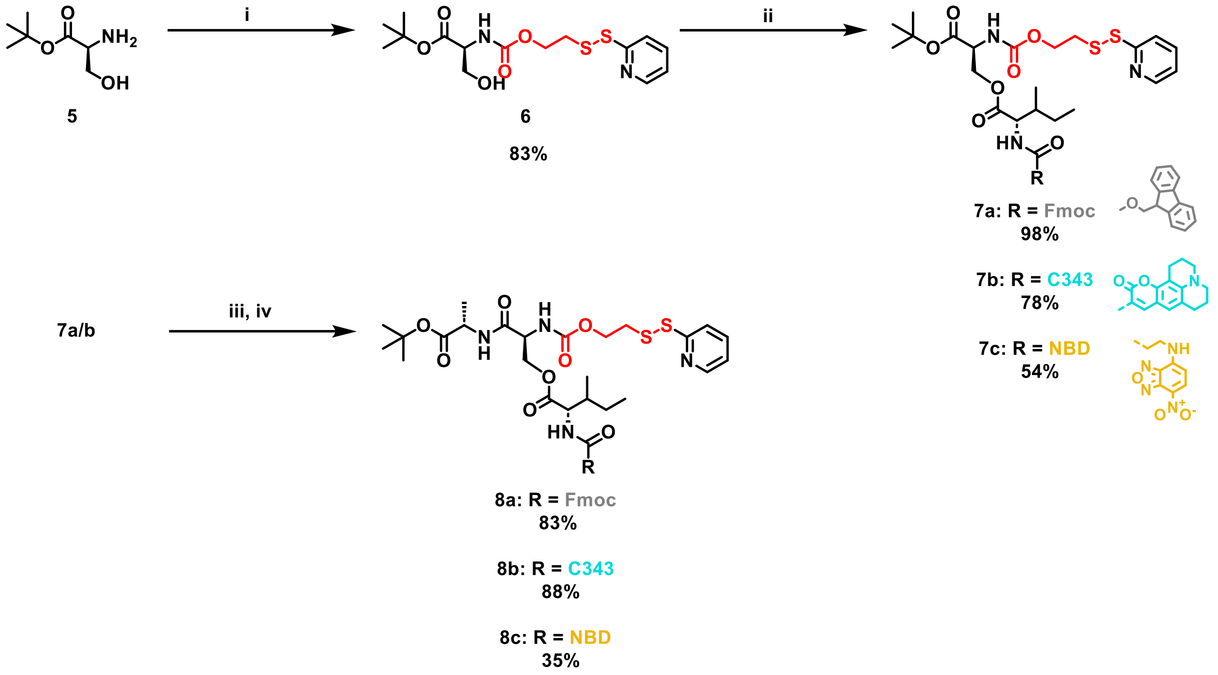

Scheme S1 Synthesis of isopeptides with activated disulfide-bonds. i) 4-nitrophenyl 2-(pyridin-2-yldisulfanyl)ethyl carbonate, DIPEA, DCM, overnight, RT; ii) Fmoc/C343/NBD-Ile, DIC, DMAP, DCM, overnight, RT; iii) TFA, DCM, 4 h, RT; iv) Ala-tBuO, PyBOP, DIPEA, DCM, 3 h, RT.

2.1.1.1. Synthesis of compound 6

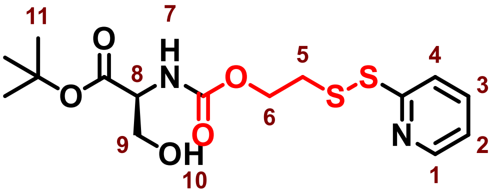

6

In a round-bottom flask, *tert*-butyl protected serine hydrochloride (200 mg, 1.01 mmol, 1 eq.) was dissolved in dry DCM (10 ml), and dry DIPEA (654 mg, 881 µL, 5.06 mmol, 5 eq.) was added to the solution. Separately, 4-nitrophenyl 2-(pyridin-2-yldisulfanyl)ethyl carbonate was dissolved in dry DCM (5 ml) and slowly added to the reaction mixture. The solution was stirred at room temperature overnight, then washed twice with water and brine. The aqueous layer was extracted with ethyl acetate (EA), and the combined organic layers were dried over sodium sulfate. After solvent removal under reduced pressure, flash column chromatography (cHex:EA, 1:1) afforded compound **6** as a colorless amorphous solid (316 mg, 0.84 mmol, 83% yield).

^{1}H NMR (400 MHz, $CDCl_3$) δ 8.43 (d, *J* = 4.9 Hz, 1H, H-1), 7.74 – 7.64 (m, 3H, H-3, H-4), 7.10 (ddd, *J* = 6.8, 4.9, 1.4 Hz, 1H, H-2), 5.73 (d, J = 7.6 Hz, 1H, H-7), 4.40 – 4.21 (m, 3H, H-6, H-6', H-8), 3.90 (dd, J = 3.6, 2.3 Hz, 1H, H-9), 3.32 (s, 1H, H-10), 3.10 – 2.94 (m, 2H, H-5), 1.46 (s, 9H, H-11).

^{13}C NMR (101 MHz, $CDCl_3$) δ 169.51, 163.21, 159.90, 155.89, 149.41, 137.59, 126.22, 121.08, 120.21, 115.82, 82.80, 63.41, 63.38, 56.74, 37.70, 28.09.

ESI-MS: *m/z* 375.1 (calculated for $[M+H]^+$: 375.10)

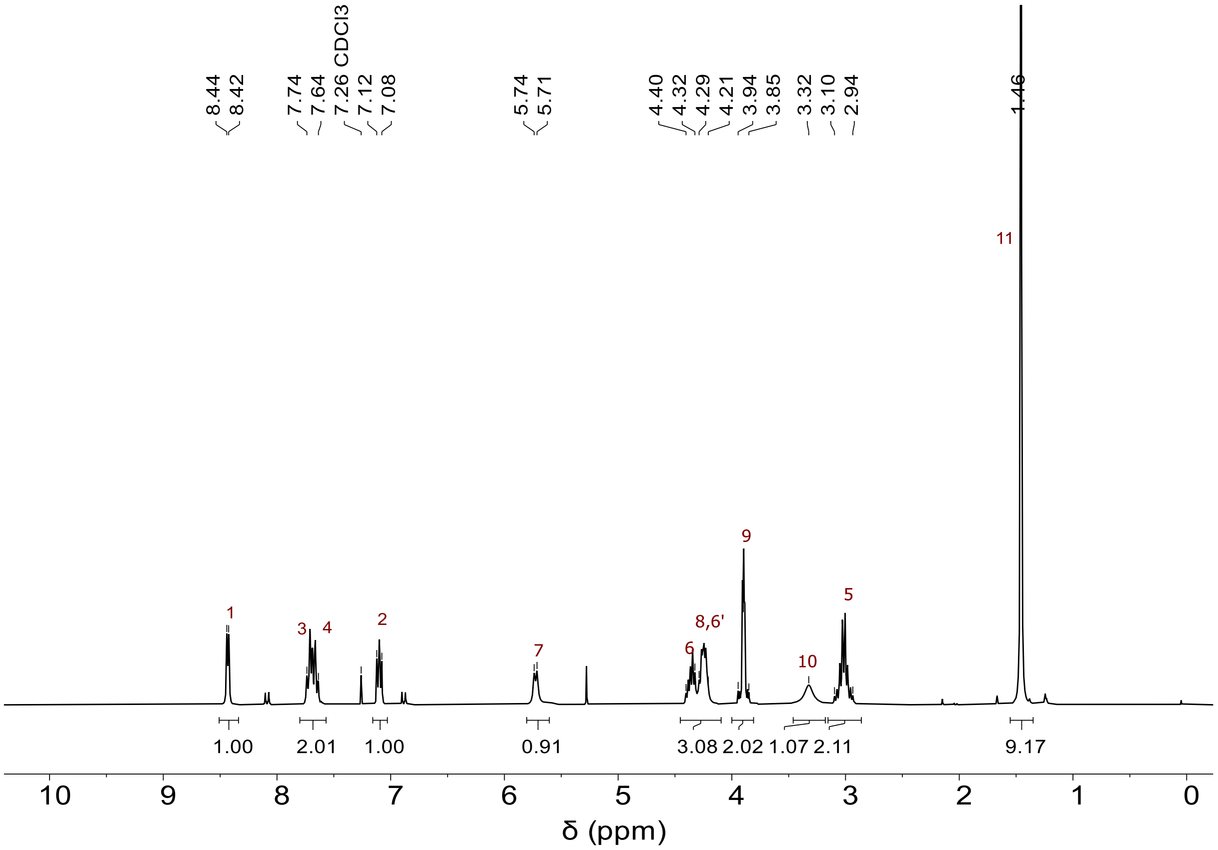

Figure S1 ^{1}H NMR spectrum (300 MHz, $CDCl_3$, 298 K) of compound **6**.

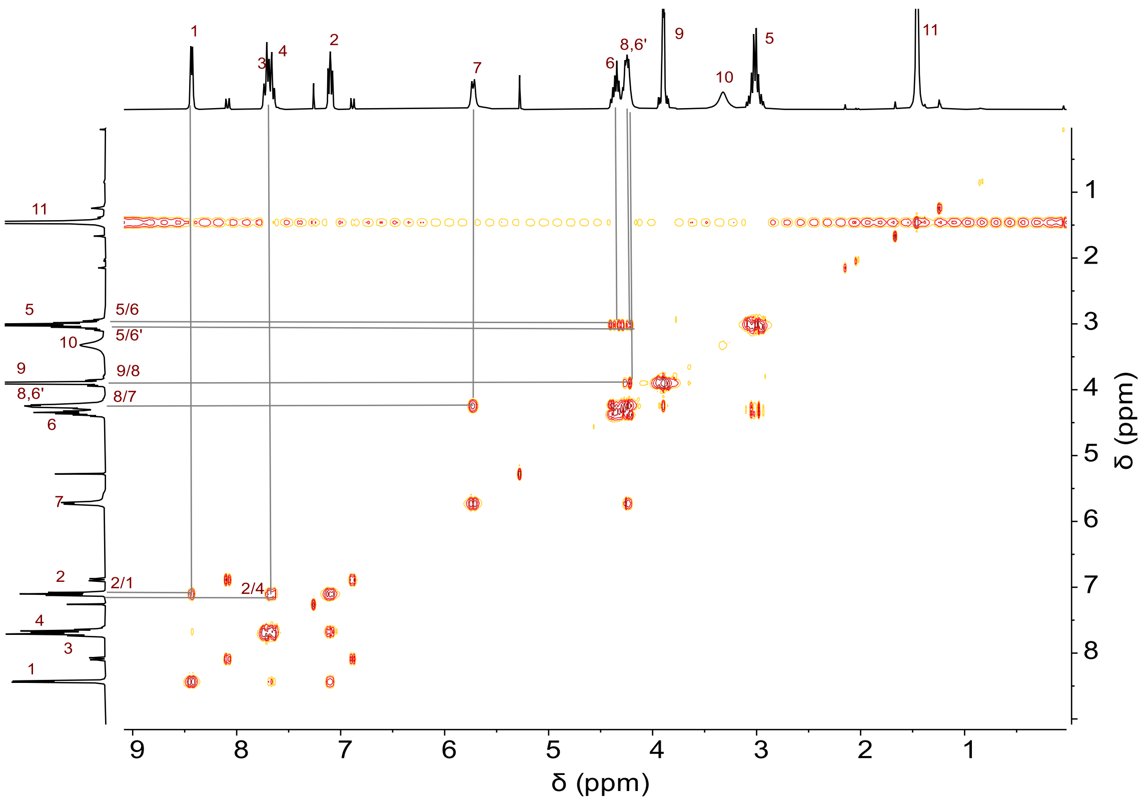

Figure S2 $^{1}H,^{1}H$ COSY NMR spectrum (300 MHz, $CDCl_3$, 298 K) of compound **6**.

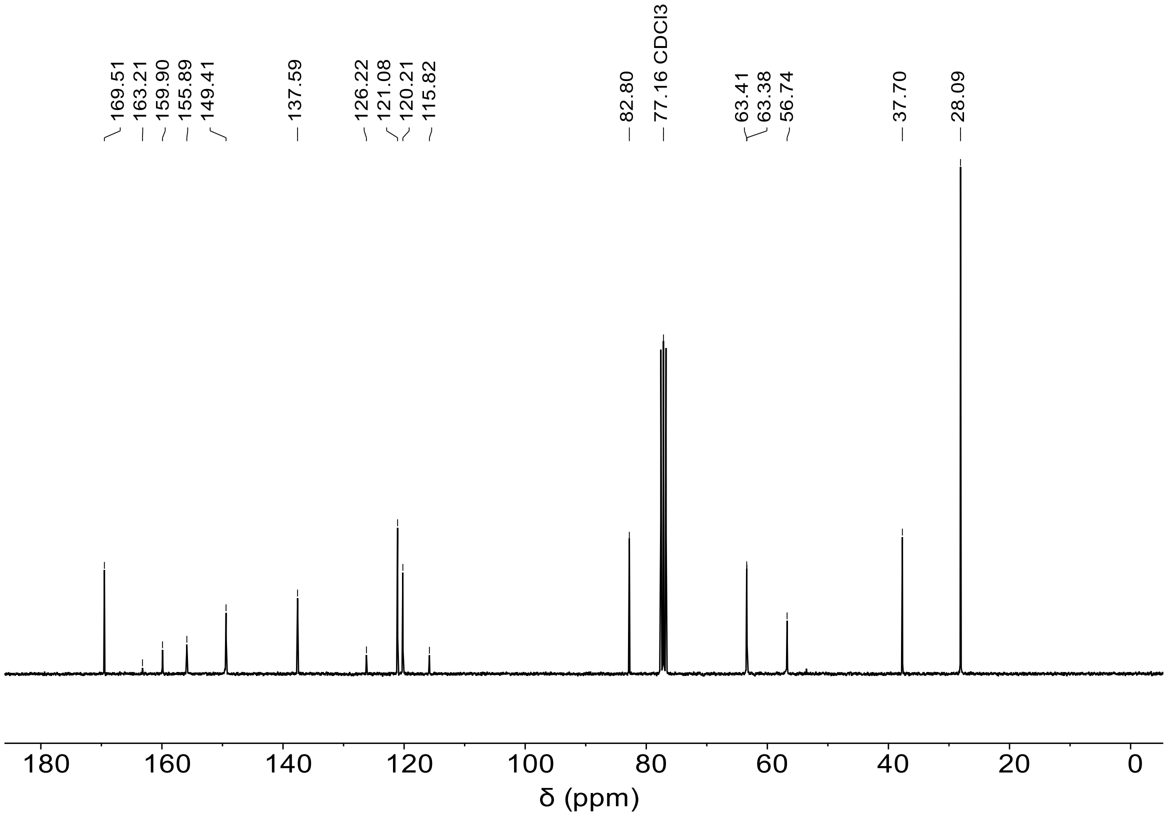

Figure S3 ^{13}C NMR spectrum (101 MHz, $CDCl_3$, 298 K) of compound **6**.

2.1.1.2. Synthesis of compounds **7a**

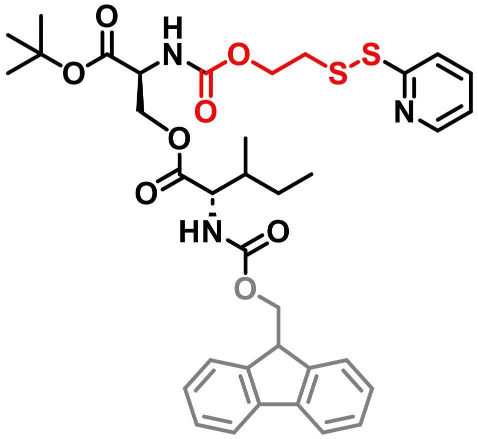

7a

In a round-bottom flask, compound **6** (157 mg, 419 µmol, 1 eq.) was dissolved in dry DCM (5 ml) under a nitrogen atmosphere. Fmoc-Ile (274 mg, 774 µmol, 1.8 eq.), DMAP (23.6 mg, 193 µmol, 0.5 eq.), and DIC (98 mg, 774 µmol, 1.8 eq.) were dissolved in dry DCM (10 ml) and added dropwise to the solution of compound **6**. The reaction mixture was stirred at room temperature overnight. After solvent removal under reduced pressure, flash column chromatography (cHex:EA, 3:1) afforded compound **7a** as a colorless oil (291 mg, 410 µmol, 98% yield).

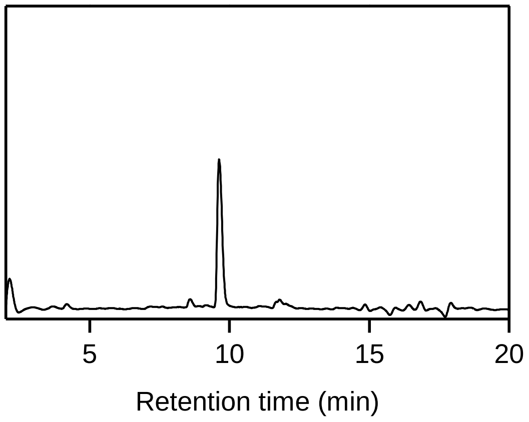

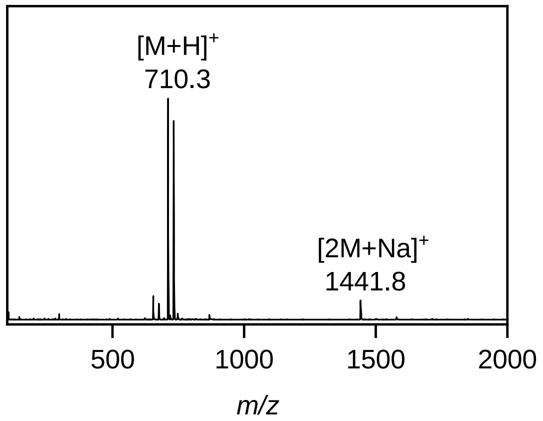

Figure S4 LC-MS data of compound **7a**. Left: LC trace (214 nm) with t_R (**7a**) = 9.63 min. Right: Convoluted ESI-MS spectrum showing peaks of $[M+H]^+$ (calculated: 710.26) and $[2M+Na]^+$ (calculated: 1441.49).

2.1.1.3. Synthesis of compounds **7b**

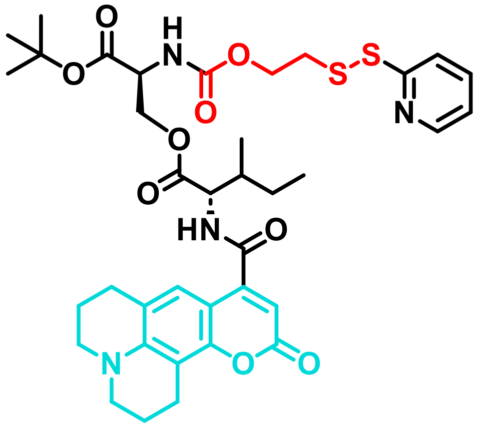

7b

In a round-bottom flask, compound **6** (80 mg, 214 µmol, 1 eq.) was dissolved in dry DCM (4 ml) under a nitrogen atmosphere. Coumarin 343-Ile (126 mg, 316 µmol, 1.5 eq.), DMAP (26.1 mg, 214 µmol, 1 eq.), and DIC (40 mg, 320 µmol, 1.5 eq.) were dissolved in dry DCM (5 ml) and added dropwise to the solution of compound 6. The reaction mixture was stirred at room temperature overnight. After solvent removal under reduced pressure, flash column chromatography (cHex:EA, 3:2) afforded compound **7b** as a bright green oil (126 mg, 167 µmol, 78% yield).

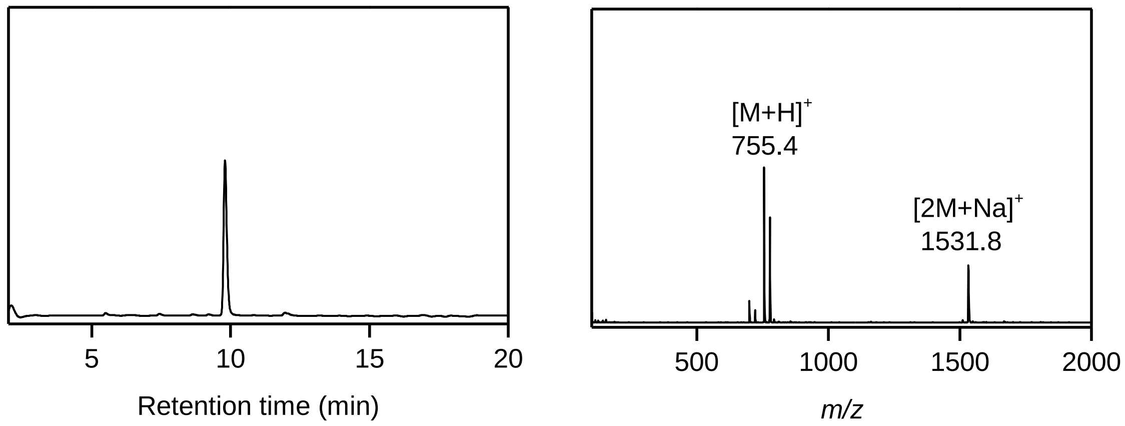

Figure S5 LC-MS data of compound **7b**. Left: LC trace (214 nm) with t_R (**7b**) = 9.80 min. Right: Convoluted ESI-MS spectrum showing peaks of $[M+H]^+$ (calculated: 755.28) and $[2M+Na]^+$ (calculated: 1531.53).

2.1.1.4. Synthesis of compounds **7c**

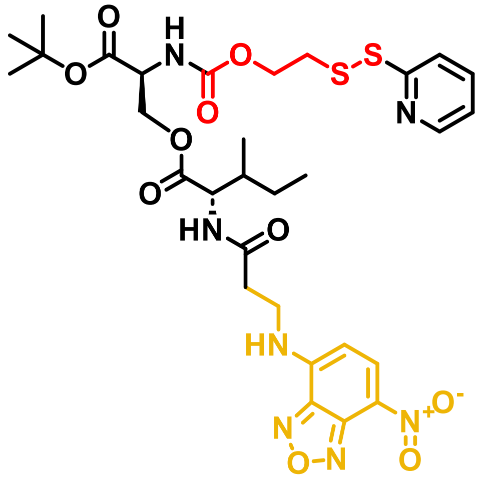

7c

In a round-bottom flask, compound **6** (162 mg, 433 µmol, 1.2 eq.) was dissolved in dry DCM (4 ml) under a nitrogen atmosphere. Nitrobenzoxadiazole (NBD)-β-Ala-Ile (132 mg, 361 µmol, 1 eq.), DMAP (88 mg, 722 µmol, 2 eq.), and DIC (91 mg, 722 µmol, 2 eq.) were dissolved in dry DCM (5 ml) and added dropwise to the solution of compound **6**. The reaction mixture was stirred at room temperature overnight. After solvent removal under reduced pressure, flash column chromatography (EA:cHex, 2:1) afforded compound **7c** as a bright yellow oil (141 mg, 195 µmol, 54% yield).

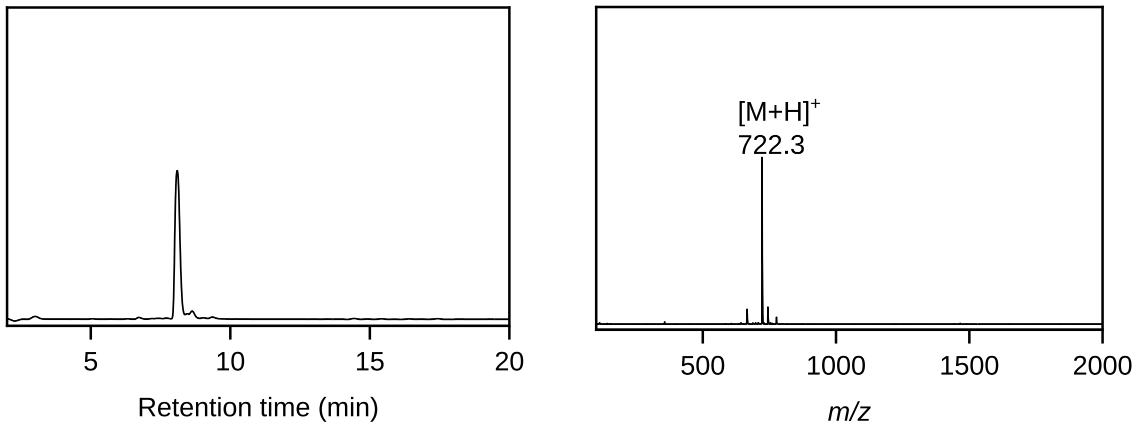

Figure S6 LC-MS data of compound **7c**. Left: LC trace (214 nm) with t_R (**7c**) = 8.63 min. Right: Convoluted ESI-MS spectrum showing the peak of $[M+H]^+$ (calculated: 722.23).

2.1.1.5. Synthesis of compound **8a**

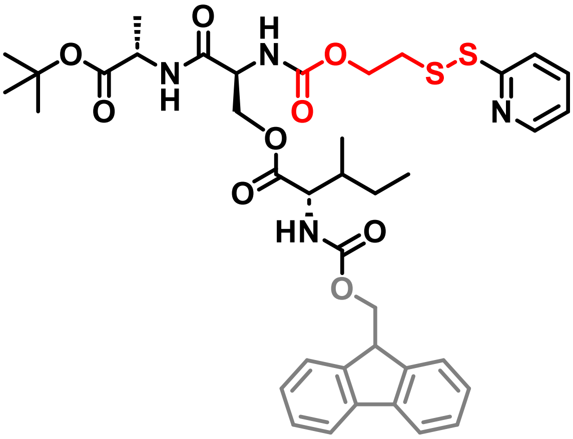

8a

In a round-bottom flask, compound **7a** (280 mg, 394 µmol, 1 eq.) was dissolved in a 1:1 mixture of TFA and DCM (4 ml:4 ml) and stirred for 6 hours. The solvents were removed under reduced pressure. The resulting colorless oil was dissolved in dry DCM (15 ml), and DIPEA (329 mg, 443 µL, 2.54 mmol, 6 eq.) was added under a nitrogen atmosphere. PyBOP (441 mg, 847 µmol, 2.2 eq.) and *tert*-butyl-protected alanine hydrochloride (154 mg, 847 µmol, 2.2 eq.) were then added, and the reaction mixture was stirred for 3 hours. The solution was washed with water, and the organic phase was extracted with ethyl acetate (EA). The combined organic layers were dried over sodium sulfate, and the solvents were evaporated. Flash column chromatography (EA:cHex, 4:3) yielded compound **8a** as a colorless oil (255 mg, 327 µmol, 83% yield).

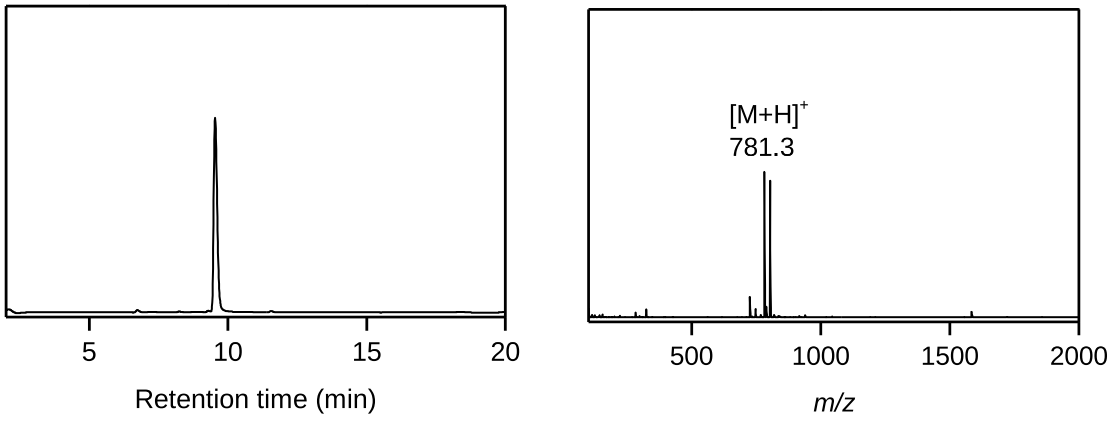

Figure S7 LC-MS data of compound **8a**. Left: LC trace (214 nm) with t_R (**8a**) = 9.53 min. Right: Convoluted ESI-MS spectrum showing peaks of $[M+H]^+$ (calculated: 781.29).

2.1.1.6. Synthesis of compound **8b**

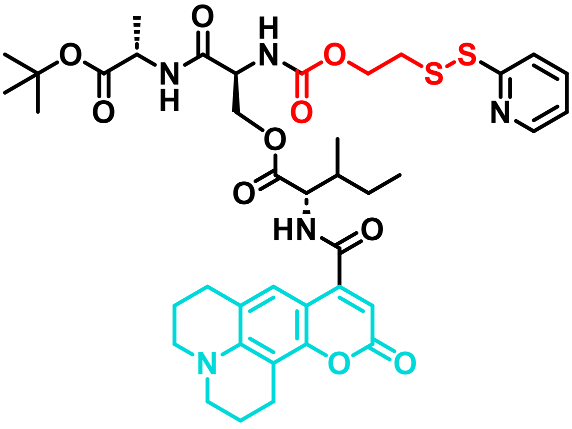

8b

In a round-bottom flask, compound **8b** (126 mg, 167 µmol, 1 eq.) was dissolved in a 1:1 mixture of TFA and DCM (2 ml:2 ml) and stirred for 4 hours. The solvents were removed under reduced pressure. The resulting bright green oil was dissolved in dry DCM (10 ml), and DIPEA (297 mg, 400 µL, 2.30 mmol, 13.7 eq.) was added under a nitrogen atmosphere. PyBOP (718 mg, 1.38 mmol, 8.2 eq.) and tert-butyl-protected alanine hydrochloride (267 mg, 1.47 mmol, 8.8 eq.) were then added, and the reaction mixture was stirred for 3 hours. The solution was washed with water and brine, and the organic phase was extracted with ethyl acetate (EA). The combined organic layers were dried over sodium sulfate, and the solvents were evaporated. Flash column chromatography (EA:cHex, 4:1) afforded compound **8b** as a bright green oil (122 mg, 148 µmol, 88% yield).

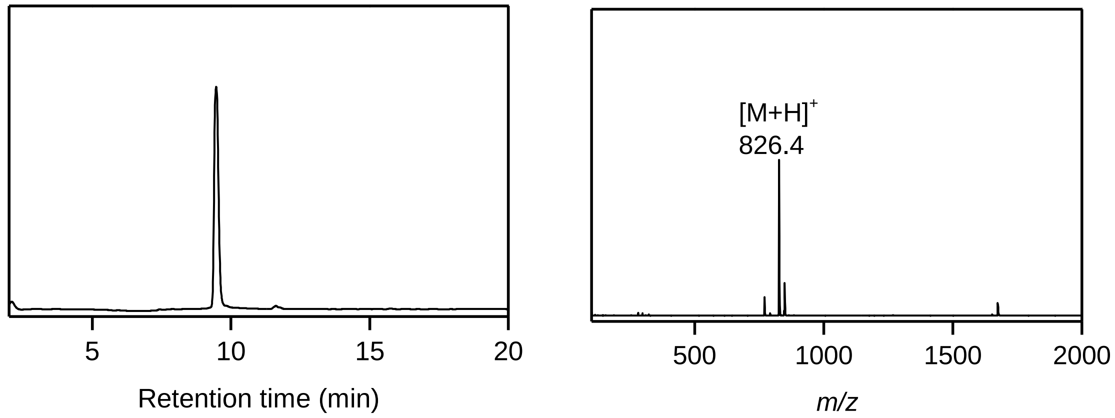

Figure S8 LC-MS data of compound **8b**. Left: LC trace (214 nm) with t_R (**8b**) = 9.47 min. Right: Convoluted ESI-MS spectrum showing peaks of $[M+H]^+$ (calculated: 826.32).

2.1.1.7. Synthesis of compound **8c**

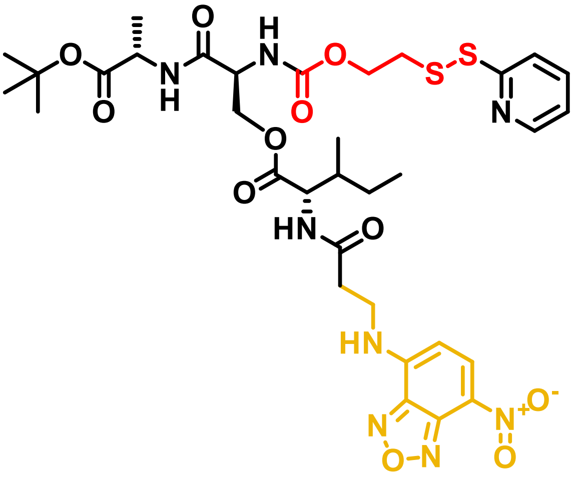

8c

In a round-bottom flask, compound **7c** (141 mg, 195 µmol, 1 eq.) was dissolved in a 1:1 mixture of TFA and DCM (2 ml:2 ml) and stirred for 4 hours. The solvents were removed under reduced pressure. The resulting bright yellow oil was dissolved in dry DCM (10 ml), and DIPEA (153 mg, 206 µL, 1.18 mmol, 6 eq.) was added under a nitrogen atmosphere. PyBOP (205 mg, 393 µmol, 2 eq.) and *tert*-butyl-protected alanine hydrochloride (71 mg, 393 µmol, 2 eq.) were then added, and the reaction mixture was stirred for 3 hours. After solvent evaporation, the product was purified via HPLC using a Phenomenex Gemini column. The gradient began with 10% CH_3CN in H2O (+0.1% TFA), held for 3 minutes, followed by a linear increase of CH_3CN to 100% over 12 minutes. This purification afforded compound **8c** as a bright yellow solid (55 mg, 69 µmol, 35% yield).

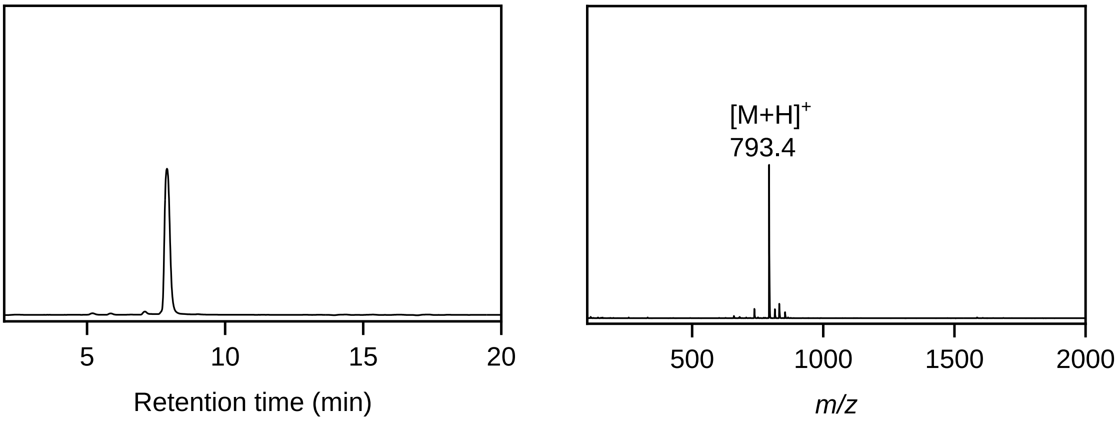

Figure S9 LC-MS data of compound **8c**. Left: LC trace (214 nm) with t_R (**8c**) = 7.89 min. Right: Convoluted ESI-MS spectrum the showing peak of $[M+H]^+$ (calculated: 793.26).

2.1.2. Solid phase supported synthesis of TAT-modified glutathione-responsive isopeptides

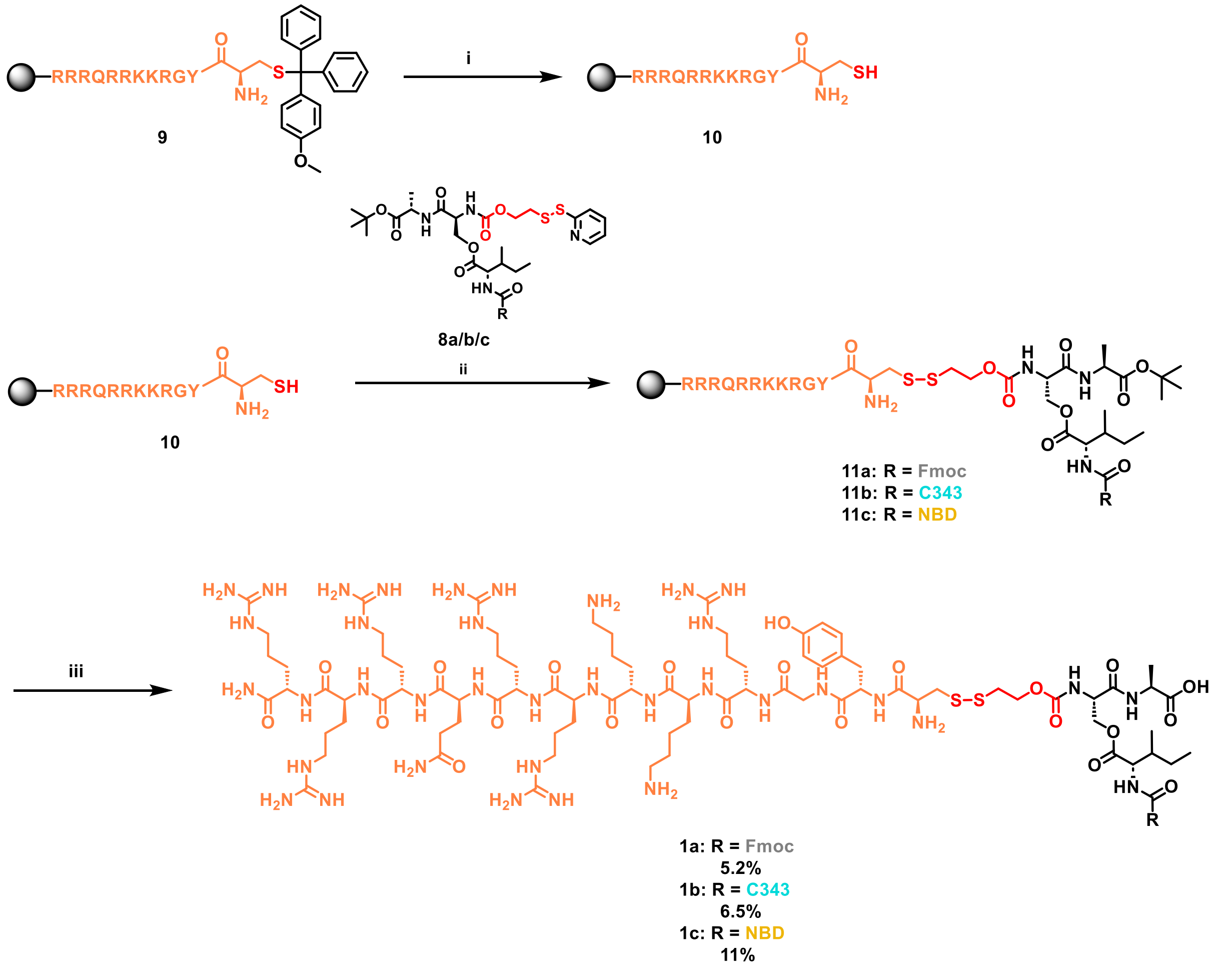

Scheme S2 Synthesis of TAT-modified glutathione-responsive isopeptides. i) TFA, TIPS, DCM (2% : 5% : 93%, 5x8 min, RT); ii) **8a/b/c**, DMF, 2 h, RT; iii) TFA, TIPS, H_2O (95% : 2.5% : 2.5%), 2.5 h, RT.

The peptide TAT, with an additional N-terminal cysteine **9**, was synthesized via solid-phase peptide synthesis (SPPS) on a Rink amide resin at a 0.05 mmol scale. The resin was first swollen in DMF for 1 hour before starting the synthesis. Prior to each coupling step, as well as during the final deprotection, the N-terminal Fmoc group was removed using two deprotection steps: 20% piperidine in DMF (3 ml) was applied for 15 seconds at 75 °C, followed by 50 seconds at 90 °C. Fmoc-protected amino acids (Fmoc-Gln(Trt), Fmoc-Lys(Boc), Fmoc-Gly, Fmoc-Tyr(tBu), and Fmoc-Cys(Mmt)) were coupled in a 5-fold molar excess (5 eq. in 1.25 ml) for 15 seconds and 110 seconds at 75 °C and 90 °C, respectively, using a solution of DIC (0.25 M) and Oxyma (0.5 M) in 2.75 ml DMF. Fmoc-Arg(Pbf) was coupled using double coupling steps for better efficiency.

The acid-labile Mmt-protecting group on the cysteine side chain was removed by treating the resin with a mixture of 2% TFA and 5% TIPS in 93% DCM (5 ml each time). This mixture was added to the resin and shaken for 8 minutes under nitrogen before draining the solution. This process was repeated five times (step i). The resin was then thoroughly washed with DCM, and dry DMF (5 ml) was added

under nitrogen. Compound **8a** (82 mg, 0.11 mmol, 2.1 eq.), **8b** (56 mg, 0.068 mmol, 1.4 eq.), or **8c** (56 mg, 0.068 mmol, 1.4 eq.) was dissolved in dry DMF (5 ml) and added to the resin, with the mixture shaken for 2 hours at room temperature (step ii). The solution was drained, and the resin was washed with DMF and DCM. To cleave the peptide from the resin, a cleavage solution (5 ml of 95% TFA, 2.5% TIPS, 2.5% H_2O) was added, and the mixture was shaken for 2.5 hours at room temperature (step iii). The peptide solution was collected by filtering out the resin, and the resin was washed with 1 ml of TFA. The combined solutions were added dropwise to ice-cold diethyl ether to precipitate the product. After centrifugation at 4,000 rpm for 20 minutes at 0 °C, the supernatant was removed, and the pellet was dried.

The resulting products (**1a**, **1b** and **1c**) were each purified via HPLC using a Phenomenex Gemini column at a flow rate of 25 ml/min. The gradient began with 5% CH_3CN in H_2O (+0.1% TFA), held for 4 minutes, followed by an increase in CH_3CN to 50% over 31 minutes. Compound **1a** was eluted after 23.69 minutes and was obtained as a white powder after lyophilization (5.9 mg, 5.2% overall yield). Compound **1b** eluted after 23.87 minutes and was obtained as a yellow powder (7.6 mg, 6.5% overall yield). Compound **1c** eluted after 20.78 minutes and was obtained as a yellow powder (9.0 mg, 11% overall yield).

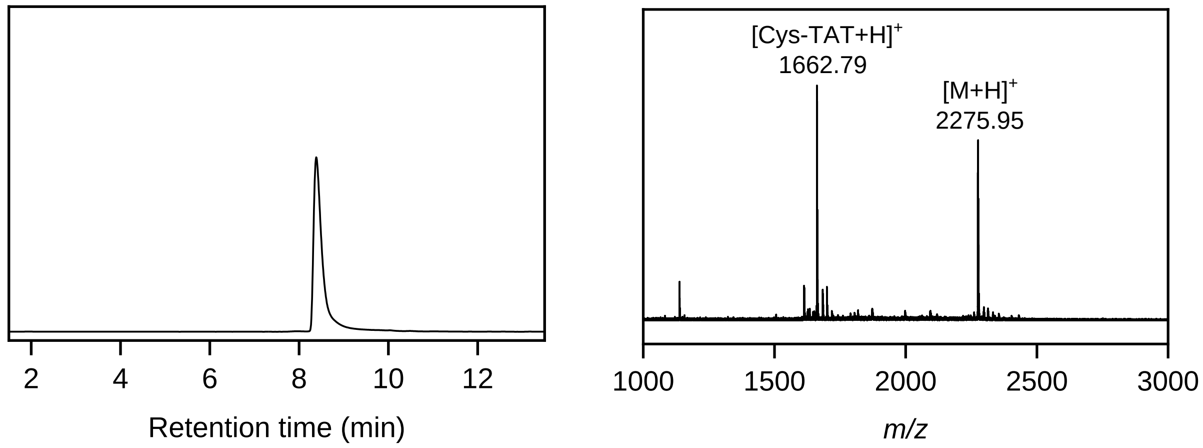

Figure S10 Analytical data of compound **1a**. Left: LC trace (214 nm) with t_R (**1a**) = 8.38 min. Right: MALDI TOF spectrum showing peaks of $[M+H]^+$ (calculated: 2276.70) and the product of disulfide cleavage $[Cys\text{-}TAT+H]^+$ (calculated: 1661.98).

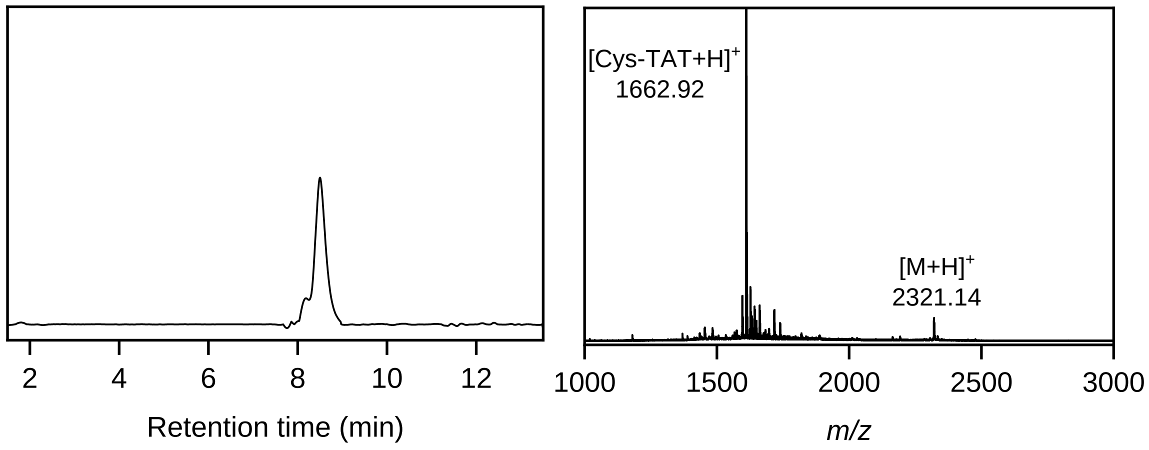

Figure S11 Analytical data of compound **1b**. Left: LC trace (214 nm) with t_R (**1b**) = 8.50 min. Right: MALDI TOF spectrum showing peaks of $[M+H]^+$ (calculated: 2321.74) and the product of disulfide cleavage $[Cys\text{-}TAT+H]^+$ (calculated: 1661.98).

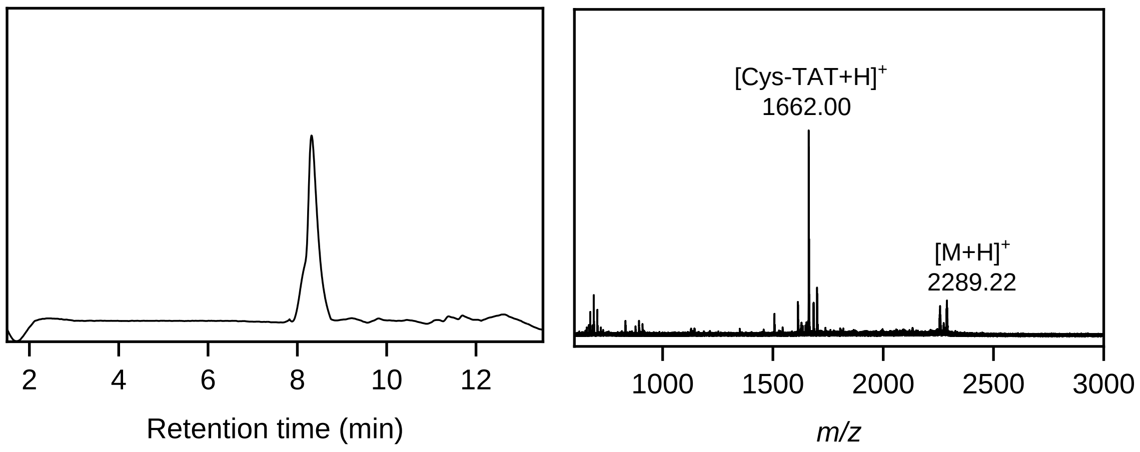

Figure S12 Analytical data of compound **1c**. Left: LC trace (214 nm) with t_R (**1c**) = 8.31 min. Right: MALDI TOF spectrum showing peaks of $[M+H]^+$ (calculated: 2288.63) and the product of disulfide cleavage $[Cys\text{-}TAT+H]^+$ (calculated: 1661.98).

2.2. Synthesis of control compounds

2.2.1. Synthesis of triethylene glycol-modified control isopeptides

2.2.1.1. Synthesis of compound **12**

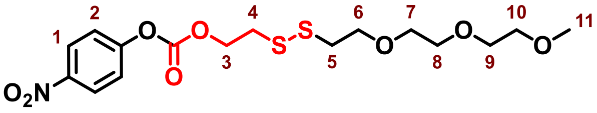

12

In a round-bottom flask 4-nitrophenyl (2-(pyridin-2-yldisulfaneyl)ethyl) carbonate (287 mg, 815 µmol, 1.4 eq.) was dissolved in a mixture of EtOH:MeOH:AcOH (85:15:5 ml) under N_2. 2-(2-(2-methoxyethoxy)ethoxy)ethane-1-thiol (106 mg, 588 µmol, 1.0 eq.) was dissolved in EtOH:AcOH (40:2 ml) under N_2 and the mixture was added dropwise to the solution of 4-nitrophenyl (2-(pyridin-2-yldisulfaneyl)ethyl) carbonate. The resulting reaction mixture was stirred for 24 h at room temperature. The solvents were removed in vacuo and subsequent flash column chromatography (cHex:EA 2:1) yielded compound **12** as a colorless oil (221 mg, 524 µmol, 89%).

^{1}H NMR (300 MHz, $CDCl_3$) δ 8.29 (d, *J* = 9.2 Hz, 2H), 7.39 (d, *J* = 9.2 Hz, 1H), 4.55 (t, *J* = 6.5 Hz, 2H), 3.75 (t, *J* = 6.5 Hz, 2H), 3.66 – 3.53 (m, 9H), 3.38 (s, 3H), 3.03 (t, J = 6.6 Hz, 2H), 2.94 (t, *J* = 6.5 Hz, 2H).

^{13}C NMR (176 MHz, CDCl3) δ 155.56, 152.46, 145.58, 125.49, 121.94, 121.91, 70.72, 70.53, 69.77, 69.58, 67.05, 66.87, 59.20, 38.74, 38.50, 36.87, 36.70.

APCI-MS: *m/z* 439.1 (calculated for $[M+NH_4]^+$: 439.12).

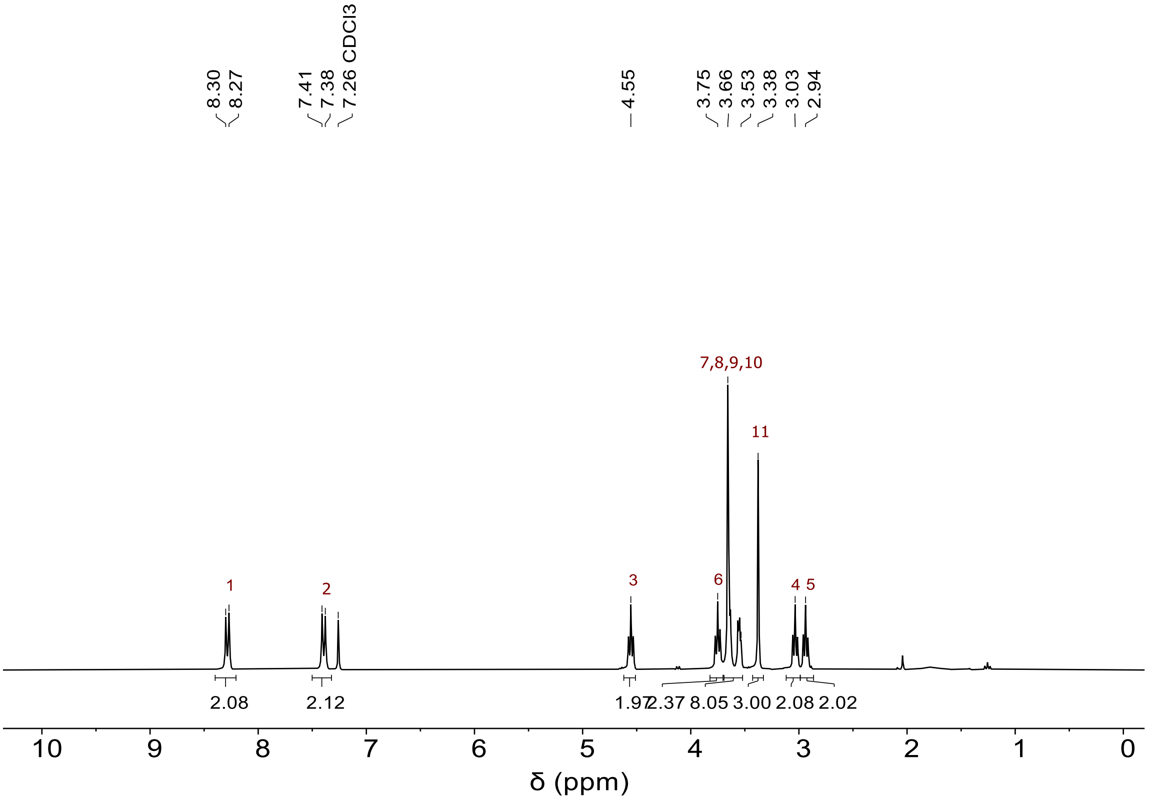

Figure S13 ^{1}H NMR spectrum (300 MHz, $CDCl_3$, 298 K) of compound **12**.

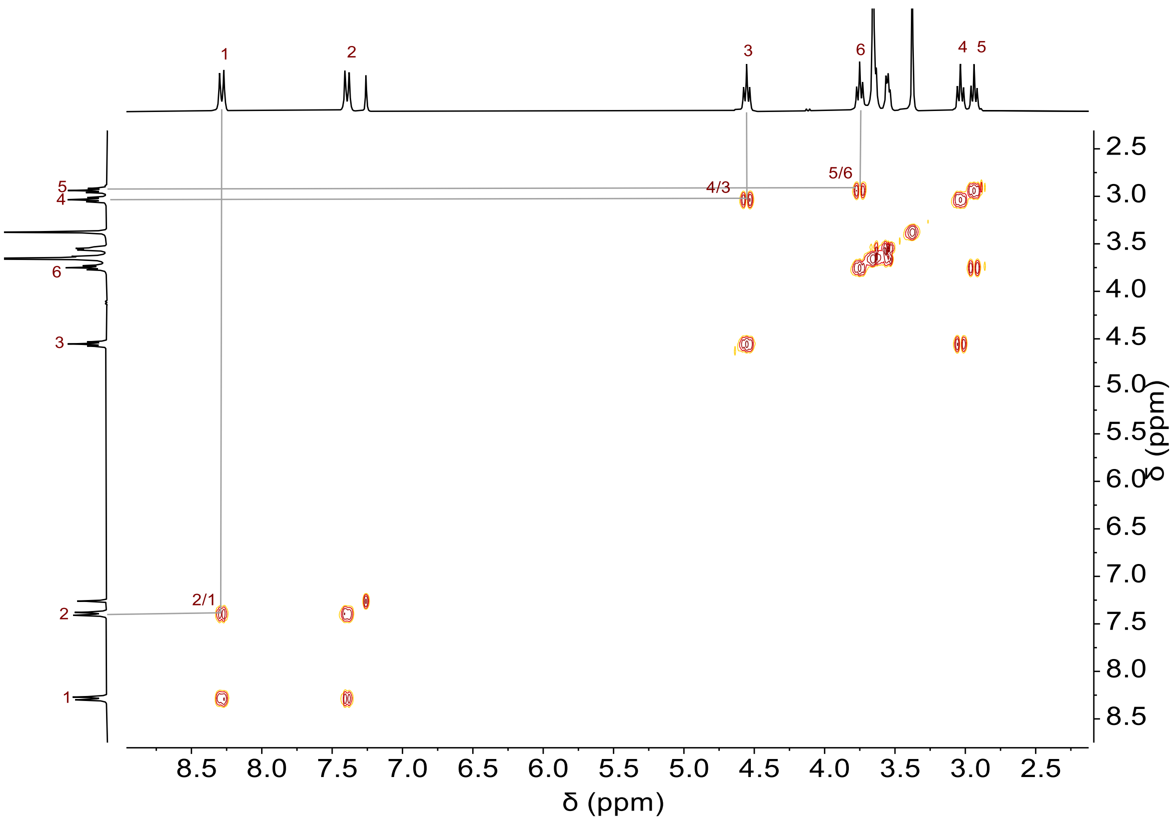

Figure S14 $^{1}H,^{1}H$ COSY NMR spectrum (300 MHz, $CDCl_3$, 298 K) of compound **12**.

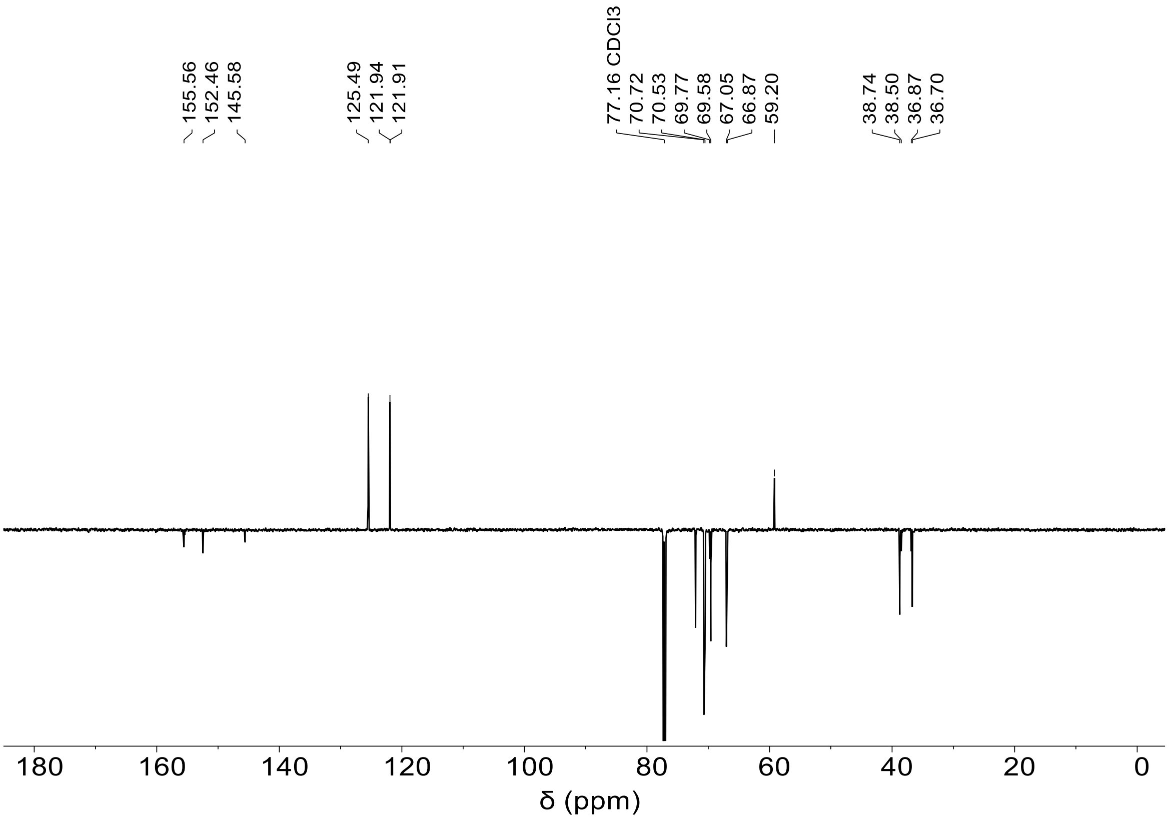

Figure S15 ^{13}C NMR spectrum (176 MHz, $CDCl_3$, 298 K) of compound **12**.

2.2.1.2. Synthesis of compound **4a**

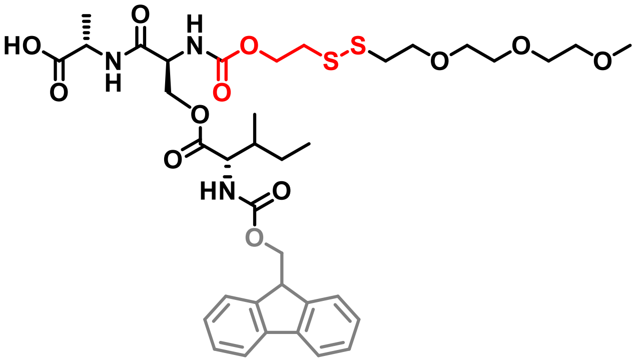

4a

The isopeptide **4a** was synthesized via solid phase supported peptide synthesis using Fmoc-Ala Wang resin at a scale of 0.5 mmol. The resin was swollen in DMF for 1 h before the synthesis. Before the coupling step and as the final deprotection, the *N*-terminal Fmoc group was cleaved by two deprotection steps using 20% piperidine in DMF (10 ml) for 25 s and 65 s at 70 °C and 90 °C respectively. Fmoc-serine without side chain protecting group (4 eq. in 10 ml) was coupled for 30 s and 120 s at 70 °C and 90 °C respectively using DIC (0.5 M) and Oxyma (1 M) in a total of 16 ml DMF. Afterwards, the resin was washed with DCM and DMF and suspended in dry DMF (5 ml) under N_2. To the suspension dry DIPEA (323 mg, 0.44 ml, 2.5 mmol, 5 eq.) was added. A solution of compound **12** (289 mg, 686 µmol, 1.4 eq.) in dry DMF (5 ml) was added dropwise to the suspended resin. The suspension was shaken at room temperature overnight and subsequently washed repeatedly with DMF. In a second step, the resin was suspended again in dry DMF (3 ml) and Fmoc-Ile (530 mg, 1.5 mmol, 3 eq.), DMAP (61 mg, 0.5 mmol, 1 eq.) and DIC (189 mg, 0.22 ml, 1.5 mmol, 3 eq.) in dry DMF (5 ml) was added dropwise. After two hours the solution was drained and the same reactants in dry DMF (5 ml) were added again. After shaking at room temperature overnight, the solution was drained and the resin was washed repeatedly with DMF and DCM. Afterwards, the cleavage solution (5 ml of 95% TFA, 2.5% TIPS, 2.5% H_2O) was added and the mixture was shaken for 2 h at room temperature. The peptide solution was collected by filtering out the resin and the resin was additionally washed with TFA (1 ml). The solvent was removed in vacuo and flash chromatography (EA:cHex 3:1 + 1% formic acid) yielded compound **4a** as a colorless solid (83 mg, 524 µmol, 21%).

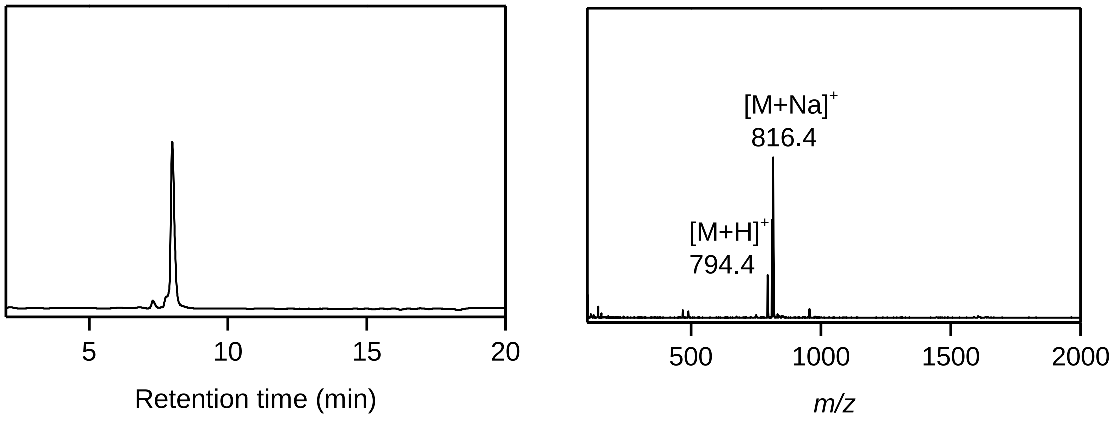

Figure S16 LC-MS data of compound **4a**. Left: LC trace (214 nm) with t_R (**4a**) = 7.99 min. Right: Convoluted ESI-MS spectrum showing peaks of $[M+H]^+$ (calculated: 794.30) and $[M+Na]^+$ (calculated: 816.28).

2.2.1.3. Synthesis of compound **4b**

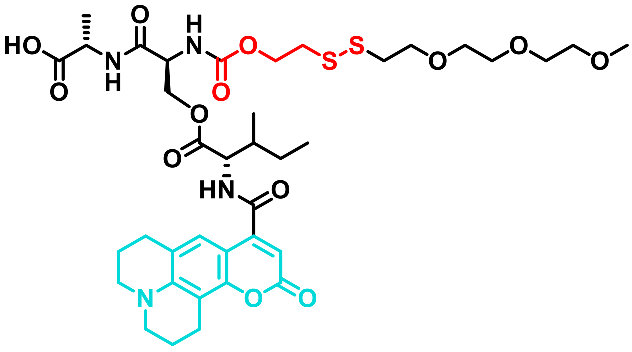

4b

In a round-bottom flask compound **8b** (55 mg, 67 µmol, 1 eq.) was dissolved in a 1:1-mixture of TFA and DCM (4 ml:4ml) and stirred for 4 h. The solvents were removed in vacuo. The remaining bright green oil was dissolved in EtOH:AcOH (1:20, 10 ml) under N_2. Afterwards, 2-(2-(2-methoxyethoxy)ethoxy)ethane-1-thiol (46 mg, 255 µmol, 3.8 eq.) was dissolved in EtOH:AcOH (5:2, 3.5 ml) under N_2 and the mixture was added dropwise to the solution of **8b**. The resulting reaction mixture was stirred for 24 h at room temperature. The solvents were removed in vacuo and subsequent flash column chromatography (DCM: MeOH 10:1) yielded compound **4b** as a bright green solid (27 mg, 524 µmol, 47%).

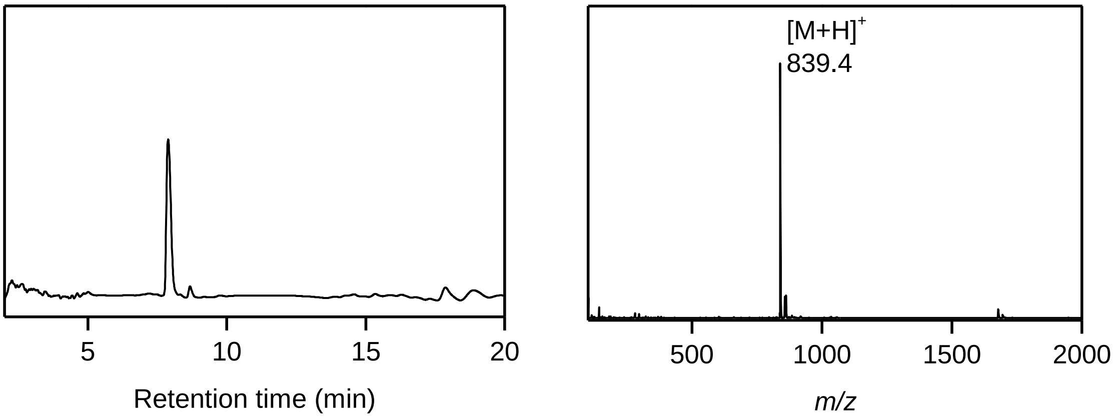

Figure S17 LC-MS data of compound **4b**. Left: LC trace (214 nm) with t_R (**4b**) = 8.68 min. Right: Convoluted ESI-MS spectrum showing peaks of $[M+H]^+$ (calculated: 839.32).

2.2.2. Analytical data of linear peptides

2.2.2.1. Analysis of compound **3a**

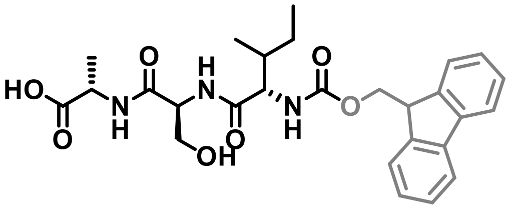

3a

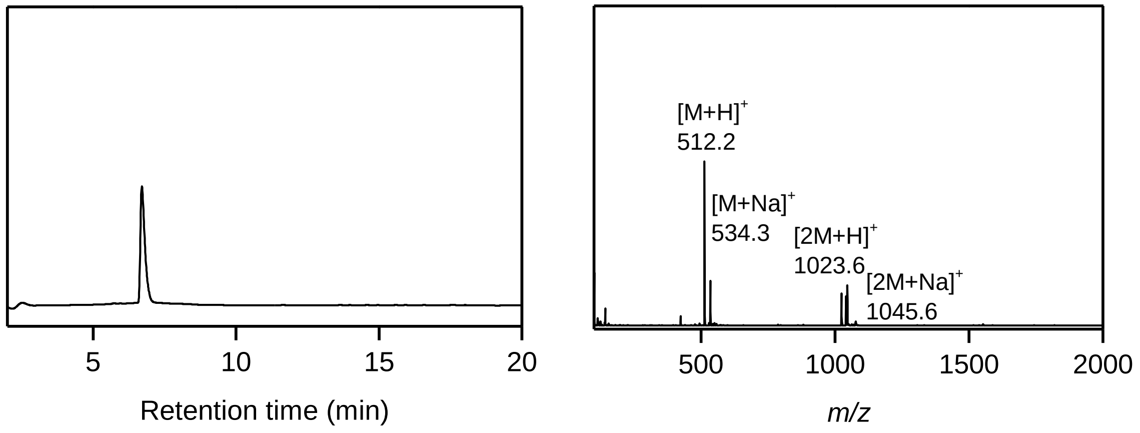

Figure S18 LC-MS data of compound **3a**. Left: LC trace (214 nm) with t_R (**3a**) = 6.71 min. Right: Convoluted ESI-MS spectrum the showing peaks of $[M+H]^+$ (calculated: 512.24), $[M+Na]^+$ (calculated: 534.22), $[2M+H]^+$ (calculated: 1023.47) and $[2M+Na]^+$ (calculated: 1045.45).

2.2.2.2. Analysis of compound **3b**

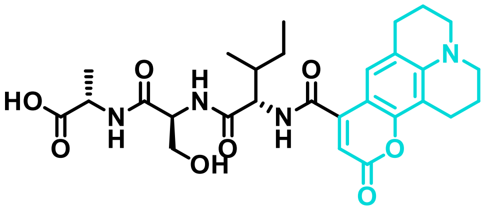

3b

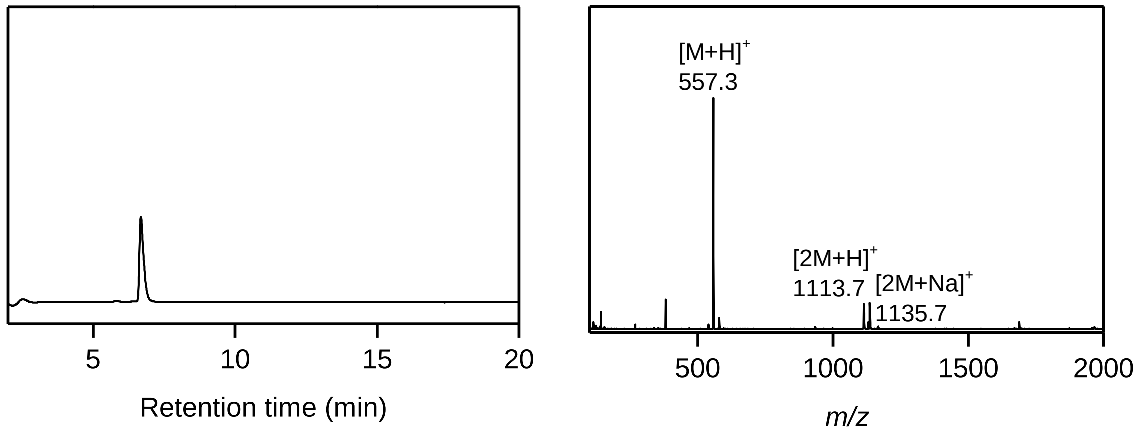

Figure S19 LC-MS data of compound **3b**. Left: LC trace (214 nm) with t_R (**3b**) = 6.68 min. Right: Convoluted ESI-MS spectrum the showing peaks of $[M+H]^+$ (calculated: 557.26), $[2M+H]^+$ (calculated: 1113.51) and $[2M+Na]^+$ (calculated: 1135.50).

2.2.2.2. Analysis of compound **3c**

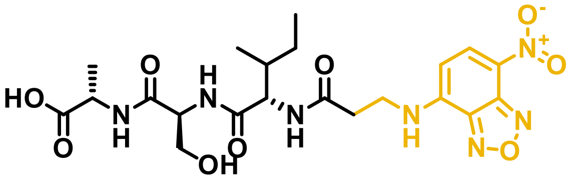

3c

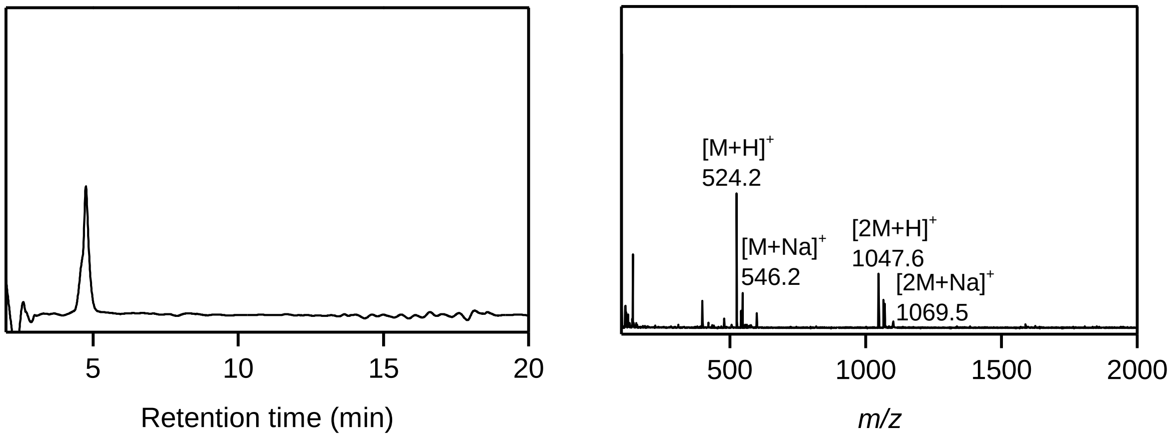

Figure S20 LC-MS data of compound **3c**. Left: LC trace (214 nm) with t_R (**3c**) = 4.75 min. Right: Convoluted ESI-MS spectrum the showing peaks of $[M+H]^+$ (calculated: 524.21), $[M+Na]^+$ (calculated: 546.19). $[2M+H]^+$(calculated: 1047.41) and $[2M+Na]^+$ (calculated: 1069.39).

3. Analysis of secondary structure and assembly behavior

3.1. TEM analysis

The linear peptides **3a**, **3b** and **3c** were each dissolved in DMSO to achieve stock solution with 10 mM or 50 mM concentration. The respective DMSO stock solutions were mixed at a 5:1 ratio of Fmoc-ISA **3a** and Coumarin 343-ISA **3b** yielding the stock solutions of the mixed linear peptides. The same was done for the kinked assembly precursors **1a** and **1b**. The DMSO stock solutions of the mixed linear peptides **3a** and **3b** and the mixed isopeptides **1a** and **1b** were diluted with DPBS at a 1:99 ratio to yield 500 µM or 100 µM peptide solutions. The same was done for the stock solutions of the linear NBD-ISA **3c** and the control isopeptide **1c**. The TEM samples were prepared as described in section 1.2.5.

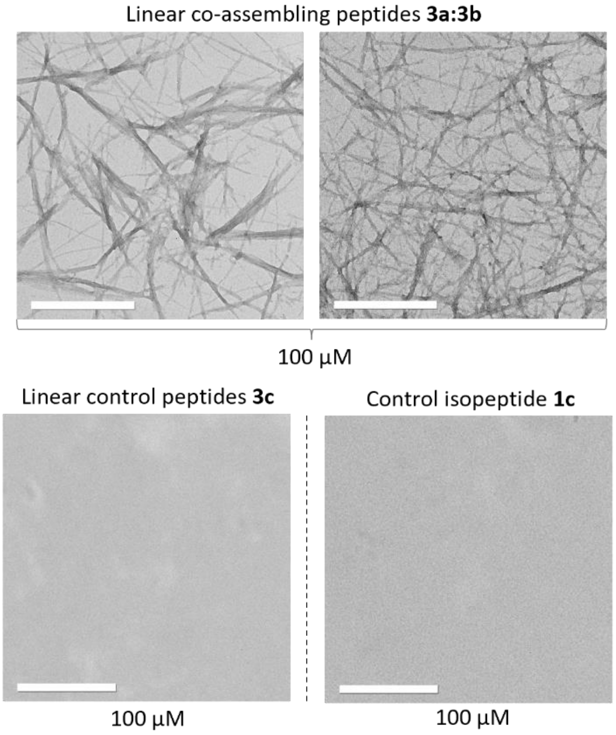

Figure S21 TEM images of nanofibers formed by co-assembling peptides **3a** and **3b** (5:1 ratio at 100 µM) in DPBS (pH 7.4) and DMSO (99:1). TEM images of linear control peptide **3c** (100 µM) and TEM images of control *iso*peptide **1c** (100 µM) in DPBS (pH 7.4) and DMSO (99:1). Scale bar 500 nm.

We conducted additional TEM analyses across a range of **3a:3b** ratios (50:1, 9:1, and 2:1) to assess the impact on assembly propensity and structural morphology. At a total peptide concentration of 500 µM, fibrillar nanostructures were observed for all tested ratios. However, at 100 µM, the 2:1 ratio of **3a:3b** resulted in shorter, less defined, and more irregular structures, whereas the 50:1 and 9:1 ratios maintained well-organized assemblies (Fig. S22). This difference likely stems from the higher proportion of Coumarin343-labeled peptide **3b** in the 2:1 ratio, which is less conducive to ordered structure formation compared to the non-labeled Fmoc-ISA **3a**.

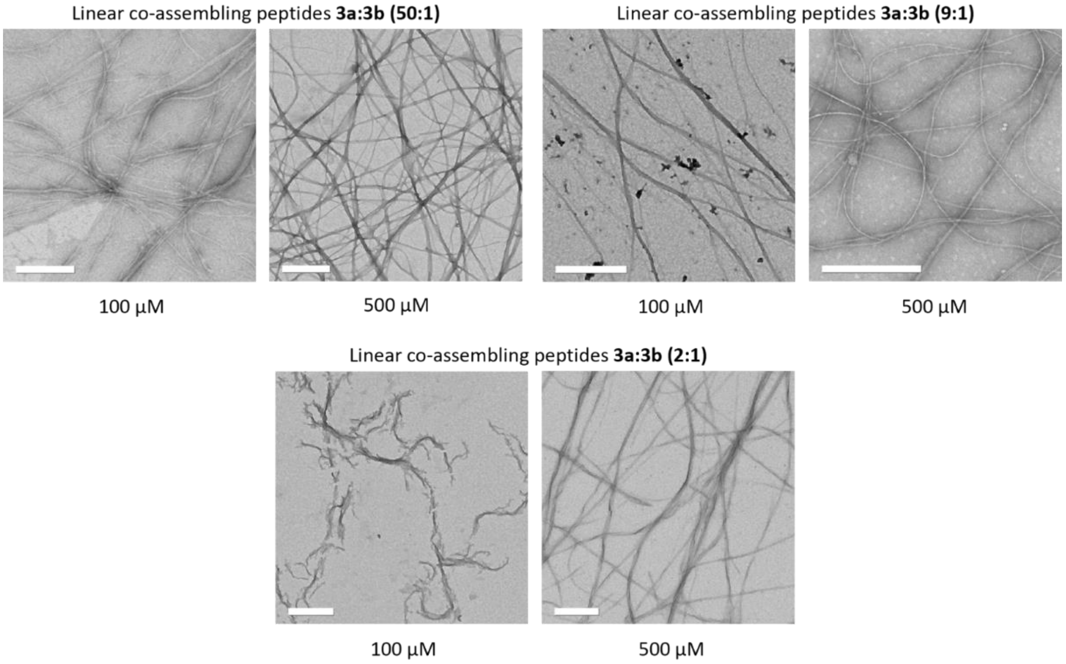

Figure S22 TEM images of nanofibers formed by co-assembling peptides **3a** and **3b** at different ratios (50:1, 9:1, 2:1) at concentrations of 100 and 500 μM in DPBS (pH 7.4) and DMSO (99:1). Scale bar 500 nm.

3.2. Proteostat assay for the determination of the critical aggregation concentration

For the analysis of the critical aggregation concentration of the linear peptides **3a:3b** (5:1) the commercial Proteostat protein aggregation assay kit by *Enzo Life Sciences, Inc.* was used as previously shown in a study by our group.[1] The peptide samples were prepared by diluting DMSO stock solutions of the linear peptides **3a:3b** (5:1) in various concentrations (1 to 100 µM) with DPBS in a 1:99 ratio. The resulting peptide samples were incubated for 24 h at room temperature while shaking. The Proteostat working solution was prepared by mixing 0.52 µl of the Proteostat stock solution with 98.48 µl MilliQ water and 1 µl assay buffer. After the incubation, 27 µl of each peptide solution were mixed with 3 µl of the Proteostat working solution. 9 µl of each solution were pipetted into a well of a Greiner 384 flat black well plate (3 wells per sample of each peptide concentration). The plate was incubated in the dark for 15 min while shaking. The fluorescence intensity of the Proteostat dye was subsequently measured with an excitation and emission bandwidth of 20 nm and an emission wavelength of 600 nm after excitation at 550 nm.

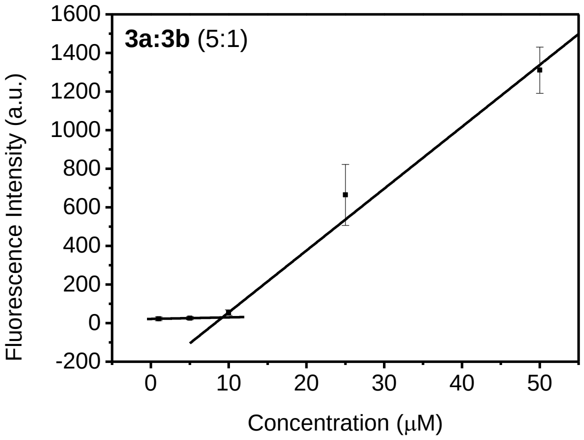

Figure S23 Proteostat aggregation assay of the mixed linear peptides Fmoc-ISA **3a** and C343-ISA **3b** (5:1) in DPBS:DMSO (99:1). The samples were incubated at room temperature for 24 h before the analysis. By calculating the intersection of the linear fits, the critical aggregation concentration of the mixed linear peptides **3a:3b** was determined to be 9.2 µM.

3.3. Stability analysis of isopeptide 1b in cell culture medium

To evaluate the stability of isopeptide **1b** in cell culture medium, **1b** was dissolved in DMEM (1% DMSO) at a concentration of 100 µM, with Fmoc-tryptophan included as an internal standard at 25 µM. The samples were incubated, and at different time points, aliquots of the mixture were collected, centrifuged (13,000 rpm, 10 min) and the supernatant was injected into an analytical HPLC system to monitor the stability of **1b** in DMEM. The results indicate no degradation of **1b** during incubation in the cell culture medium.

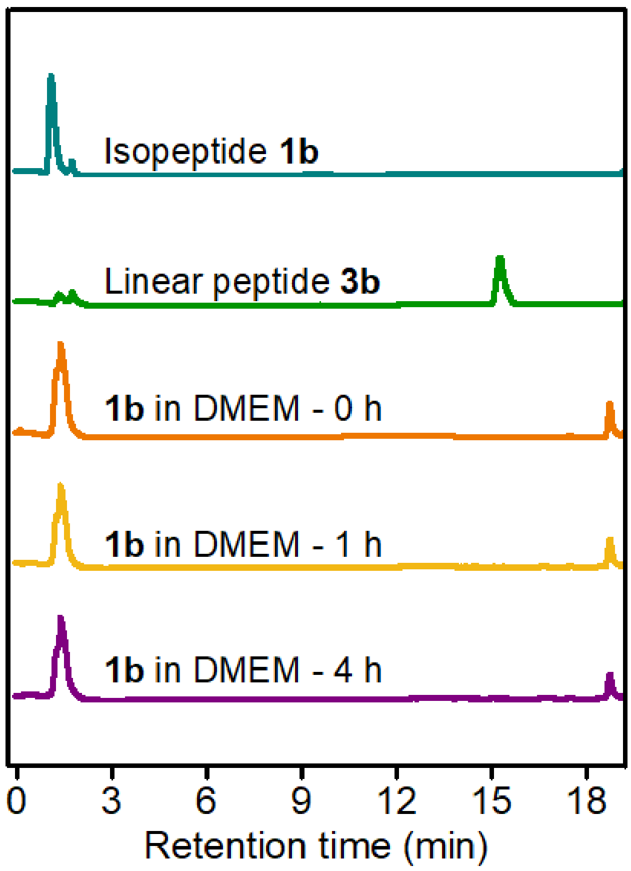

Figure S24 Stability analysis of isopeptide 1b (100 µM) in DMEM cell culture medium (1% DMSO) over time. Representative elugrams of isopeptide **1b** (t_R = 1.43 min) and its degradation product, linear peptide **3b** (t_R = 15.32 min), are shown for comparison. Fmoc-tryptophan (25 µM, t_R = 18.78 min) was used as an internal standard.

4. Cell Experiments in 2D cell culture

4.1. Cell uptake analysis

MDA-MB-231 cells were seeded at a density of 20,000 cells/well in an 8-well confocal plate. After adhering for 24 h, cells were treated with the samples containing either **1a:1b** or the control compounds **1c**, **3a:3b** or **4a:4b** at different concentrations and incubated for 4 h at 37 °C. The sample solutions were prepared by dissolving the respective compounds in DMSO to prepare different stock solutions (100x) containing **1a:1b**, **3a:3b** or **4a:4b** in a 5:1 molar ratio or **1c**. These stocks were each diluted with DPBS in a 9:1 ratio yielding the DPBS:DMSO stocks (10x). Another dilution with DMEM (9:1) gave the final samples (total DMSO content: 1%) which were added to the cells after removing the old medium. After the incubation time of 4 h was over, the cells were fixed for 15 min at room temperature using 4% paraformaldehyde solution. The nucleus was stained with HCS Nuclear Mask Deep Red Stain for 20 min at room temperature. The staining solution was removed, and fresh DPBS was added to the cells before they were imaged by confocal laser scanning microscopy. Confocal images of cells were taken on a Leica Stellaris® 8 microscope.

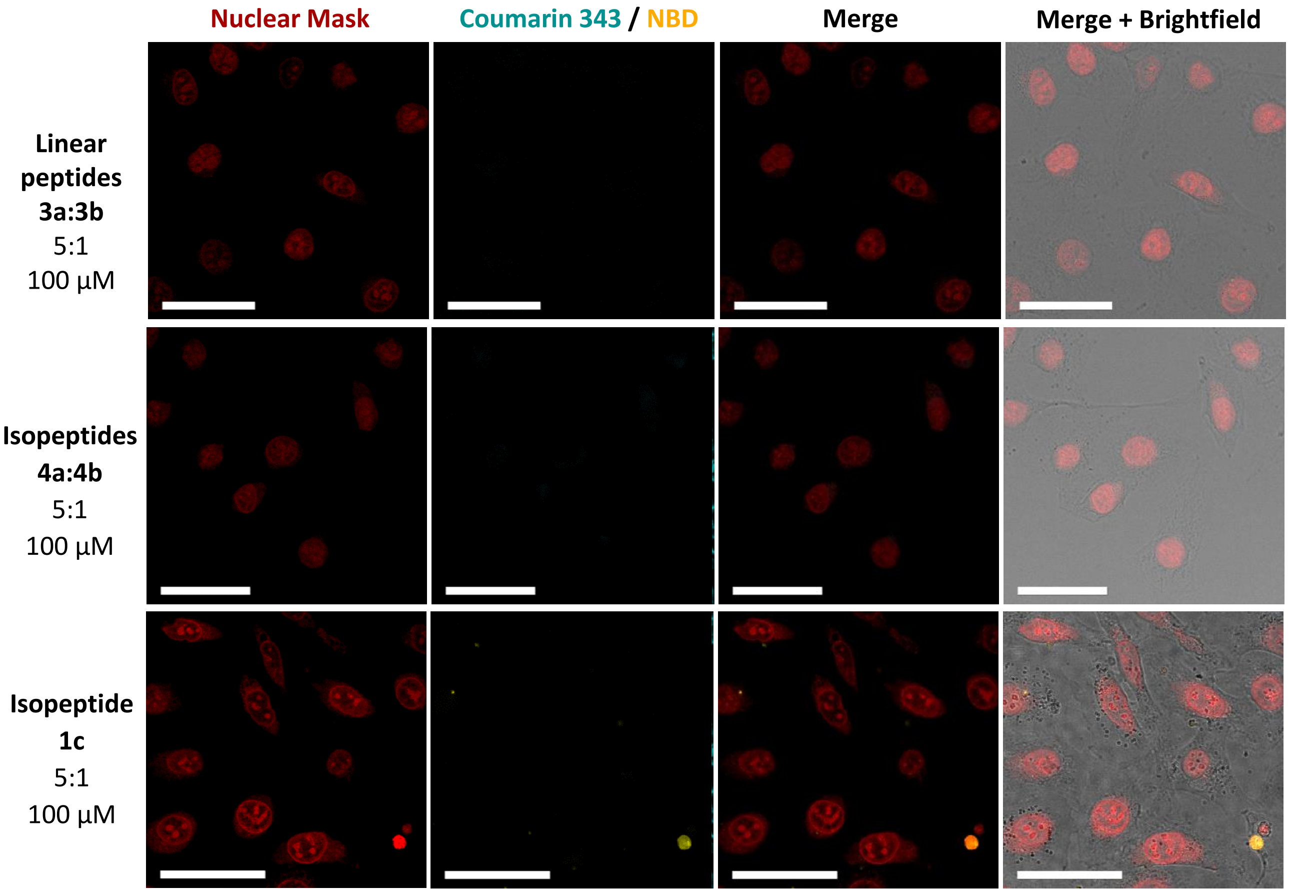

Figure S25 Cell uptake in cancer cells. Confocal laser scanning microscopy (CLSM) images of MDA-MB-231 breast cancer cells after 4 h of incubation with control compounds **3a:3b**, **4a:4b** (5:1) or **1c** at 100 µM. The nucleus was stained with HCS Nuclear Mask Deep Red (red). Scale bars 50 µm.

4.2. Live-cell imaging

MDA-MB-231 cells were cultured in an 8-well confocal plate at a density of 20,000 cells per well in DMEM and allowed to adhere overnight at 37°C, 5% CO_2. The medium was removed and the sample of **1a:1b** in DMEM/DPBS/DMSO (90%/9%/1%) was introduced into a well. The cells were imaged live using an incubator-equipped Leica Stellaris® 8 microscope (40x glycerol immersion objective) with fast lifetime contrast (FALCON) module (Leica Microsystems GmbH). The incubator (okolab) is set and held constant at 37 °C, 5% CO_2 and a relative humidity of 90% throughout all measurements.

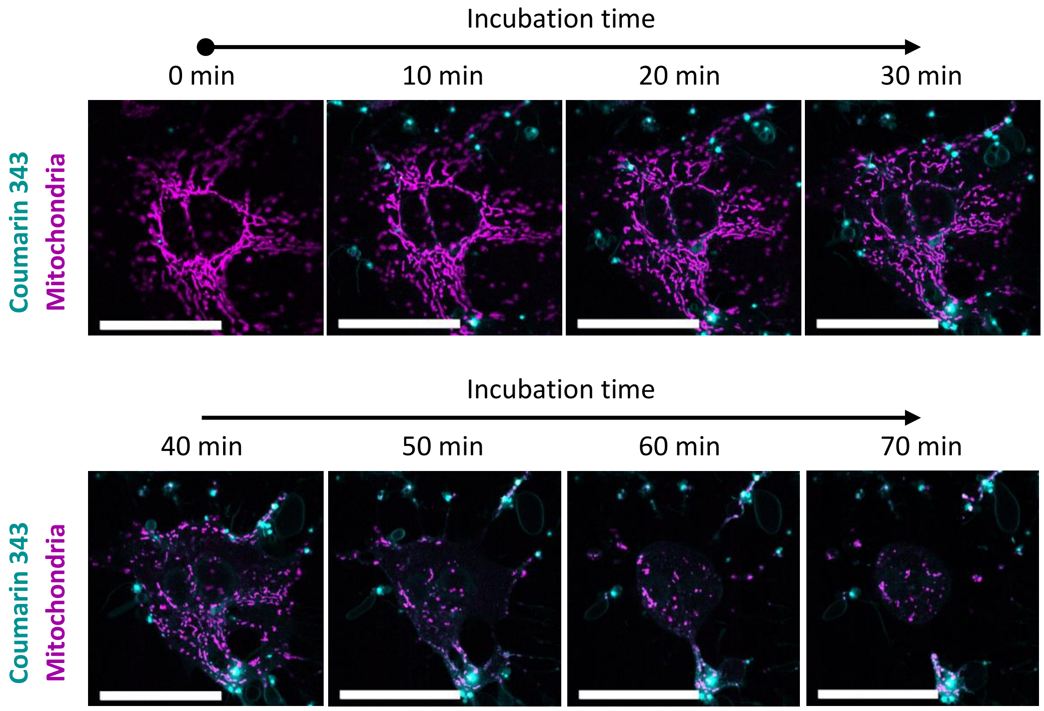

Figure S26 Live cell imaging of mitochondria-stained cancer cells. CLSM images of live-cell imaging of MDA-MB-231 cells treated with 50 µM of **1a:1b** (cyan). Cells were previously transfected with CellLight™ Mitochondria-RFP (pink). Scale bars 50 µm.

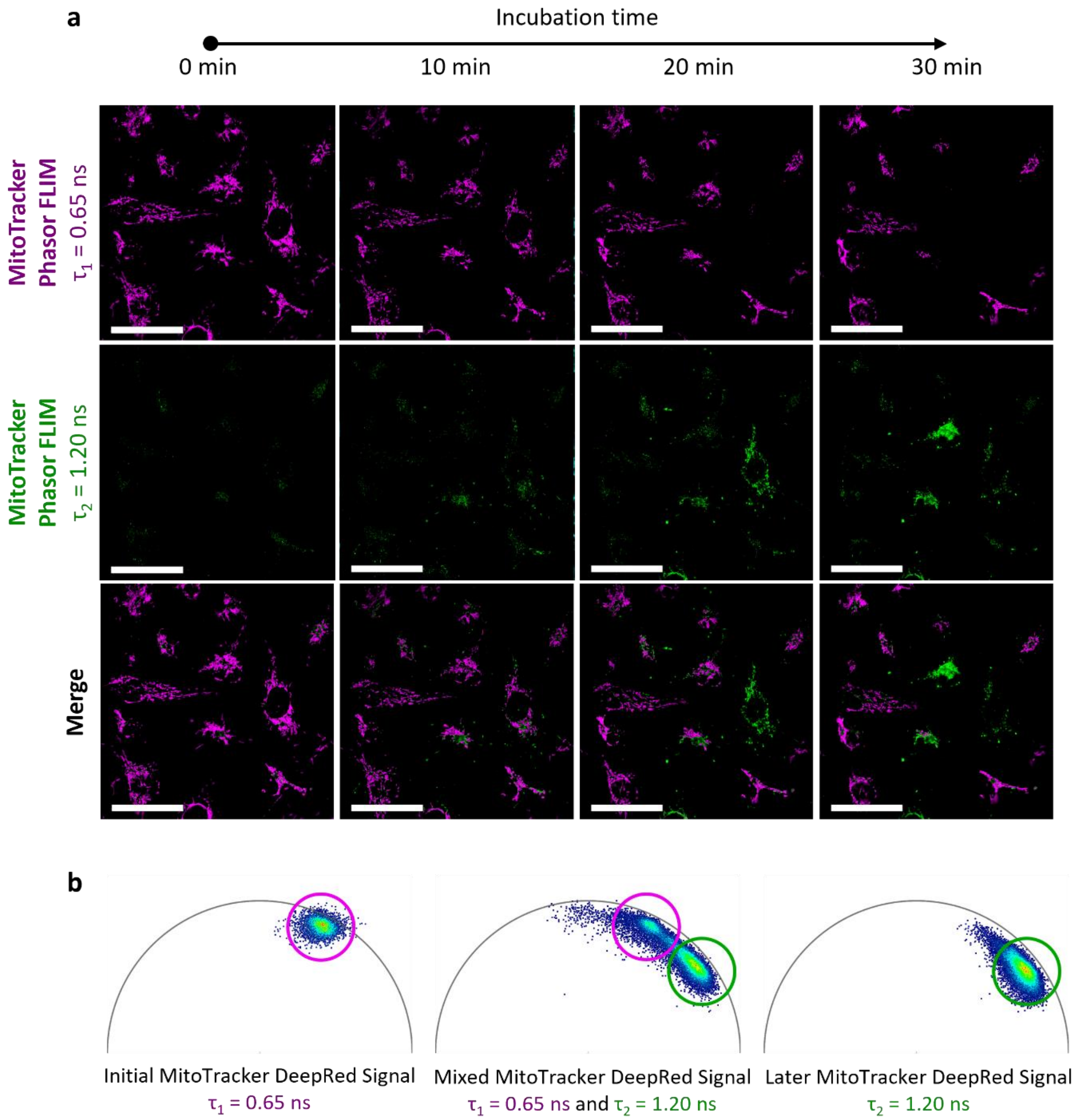

Figure S27 Phasor plots of MitoTracker DeepRed FLIM measurements. Live-cell imaging of MDA-MB-231 cells treated with 50 µM of **1a:1b**. Cells were previously stained with MitoTracker DeepRed. Fluorescence lifetime measurements of the stain were conducted concurrently with fluorescence imaging, revealing two distinct fluorescence lifetime signals for MitoTracker DeepRed over time. Scale bars 50 µm.

4.3 Correlative light- and electron microscopy (CLEM) of cells

For electron microscopy (EM) studies correlated with confocal light microscopy, MDA-MB-231 cells were seeded at a density of 20,000 cells per well on sapphire disks and incubated with 100 µM of **1a:1b** for 4 h. After incubation, the cells on the sapphire disks were frozen using high-pressure freezing (Wohlwendt HPF 01). Freeze substitution followed, with the cells stained at low temperatures using OsO_4 and uranyl acetate for EM examination. The cells were then embedded in epoxy resin (EPON), and ultrathin sections were prepared with an ultramicrotome (Leica FC7) using a diamond knife (Diatome). The section thickness and substrate were adjusted for subsequent EM investigation: for TEM, sections (100 nm thick) were transferred to Cu grids; Fluorescence of the co-assembling peptides was first measured in a confocal laser scanning microscope (Leica SP5) (458nm excitation, 470-500 nm emission detection bandwidth). The same thin section was then examined in TEM (FEI Tecnai F20). To identify the fluorescent peptide assemblies, we correlated fluorescence with EM images by superimposing the images using the "Landmark correspondence" plugin in ImageJ 1.53e.

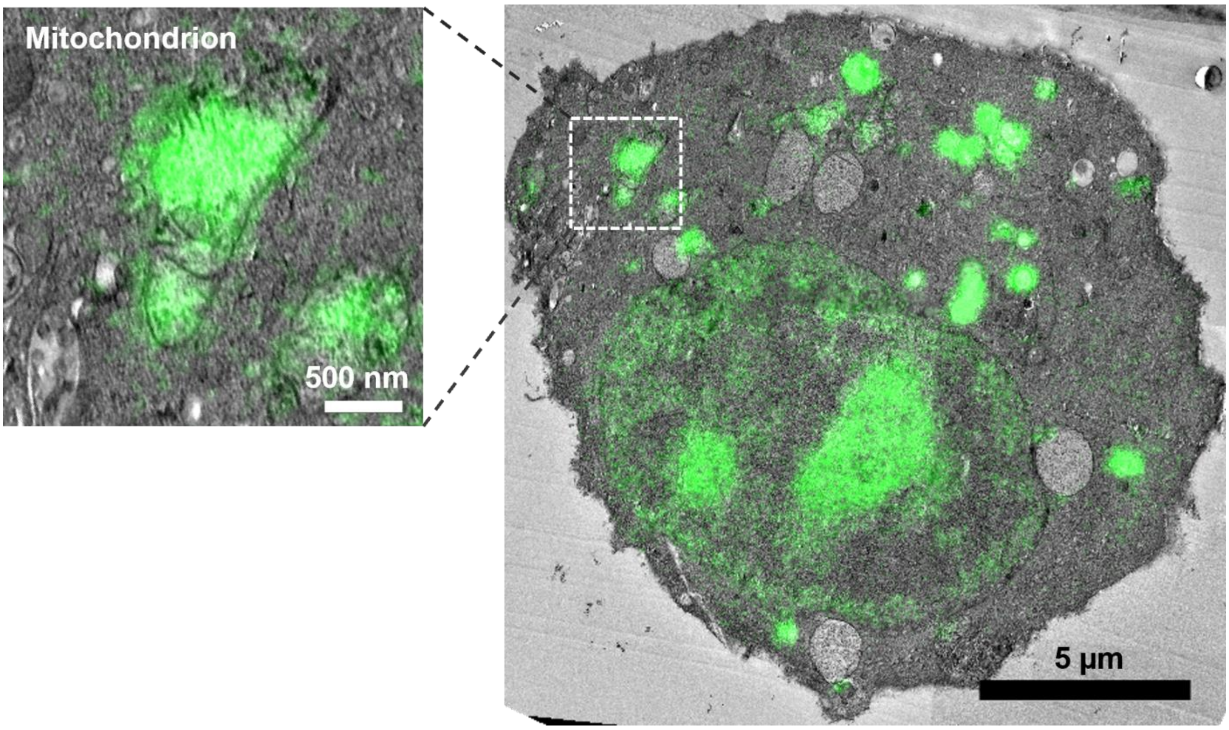

Figure S28 Correlative light- and electron microscopy (CLEM) image of MDA-MB-231 cell incubated with **1a:1b** (100 µM) (green) for 4 h.

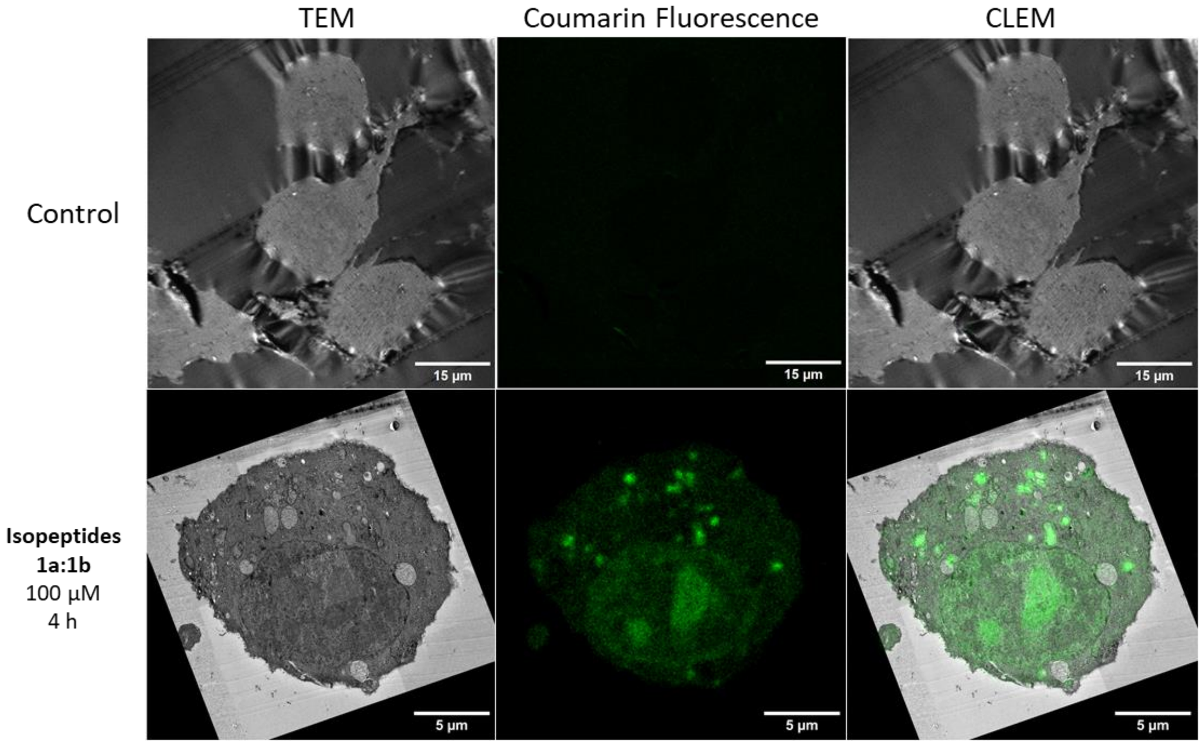

Figure S29 Correlative light- and electron microscopy (CLEM) image of an untreated MDA-MB-231 cell as control (first row) compared to a cell incubated with **1a:1b** (100 µM) for 4 h (second row).

4.4. Co-staining of actin filaments

For the co-staining of actin filaments MDA-MB-231 cells were seeded at a density of 20,000 cells/well in an 8-well confocal plate. After adhering for 24 h, cells were treated with the samples containing **1a:1b** at different concentrations and incubated for 4 h at 37 °C. The sample preparation is described in section 3.1. After the incubation the medium was removed and the cells were washed with DPBS and fixed for 15 min at room temperature using 4% paraformaldehyde solution. After rinsing with DPBS three times, the actin filament stain Alexa Fluor™ 555 Phalloidin was added to the cells (diluted 1:20 in DPBS) and incubated for 15 min at room temperature. Afterwards the cells were washed once with DPBS and fresh DPBS was added before imaging.

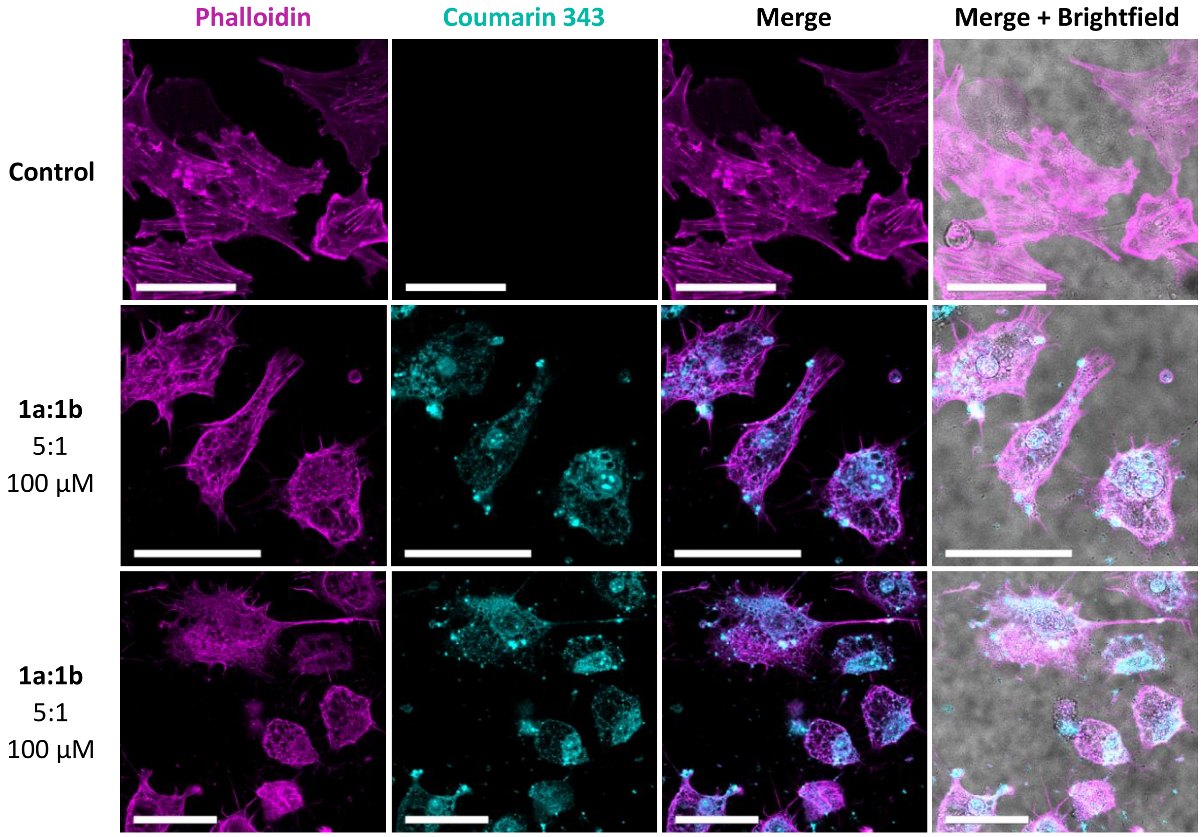

Figure S30 Disruption of actin filaments by in-situ formed nanostructures. Confocal laser scanning microscopy (CLSM) images of MDA-MB-231 cells after 4 h of incubation with glutathione-responsive isopeptides **1a** and **1b** (5:1) (cyan) and additional staining with Alexa Fluor™ 555 Phalloidin (pink). Scale bars 50 µm.

To assess the time-dependent effects on cytoskeletal integrity, we also performed phalloidin staining on cells treated with the assembly-inducing isopeptides **1a:1b** (at 100 and 10 µM) and the linear peptides **3a:3b** (at 100 µM) as a control. Imaging at 1, 2, 4, and 24 hours revealed that structural disruption of the cytoskeleton occurs rapidly, with pronounced effects already evident within 1 hour of incubation (Fig. S29-S31). The extent of cytoskeletal damage remained consistent at later time points, indicating that the effect is an early and sustained response to peptide treatment.

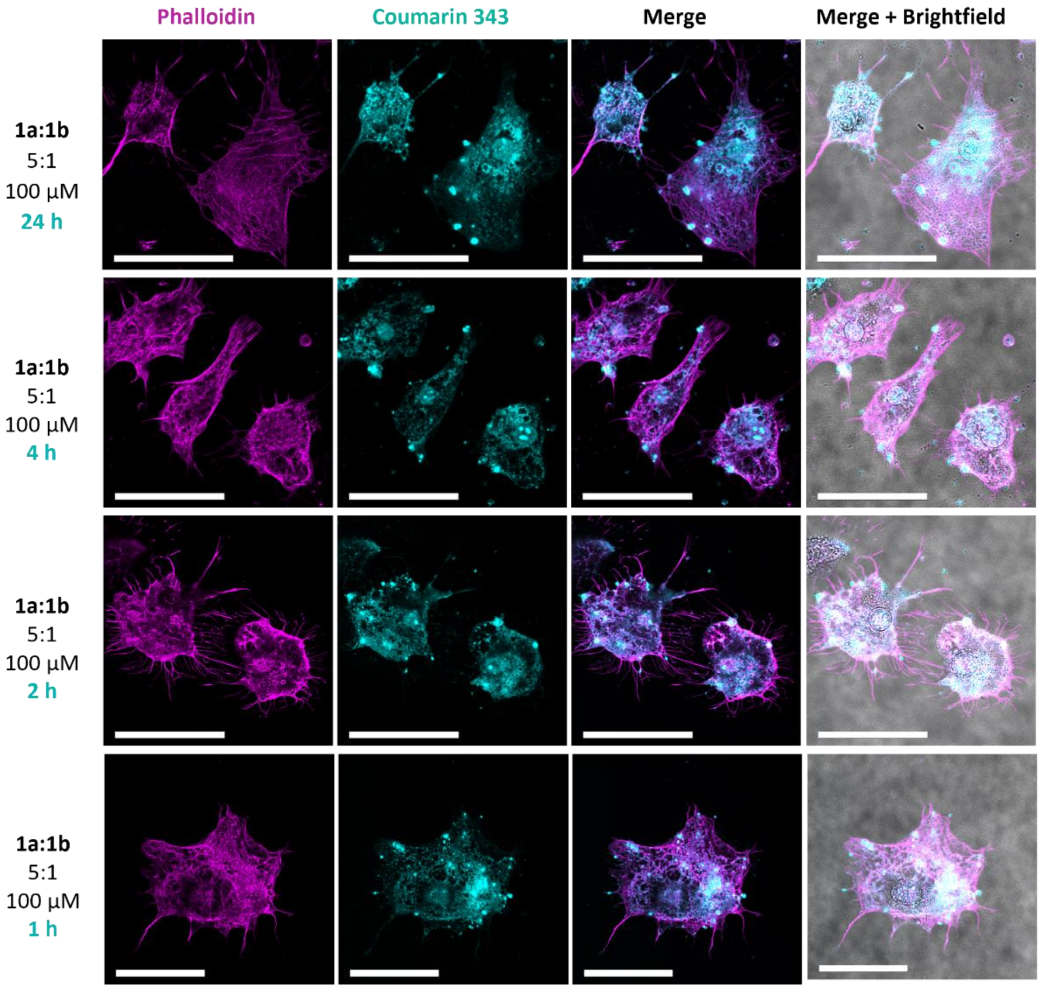

Figure S31 Confocal laser scanning microscopy (CLSM) images of MDA-MB-231 cells after 1, 2, 4 and 24 h of incubation with glutathione-responsive isopeptides **1a** and **1b** (5:1) (cyan) at 100 µM and additional staining with Alexa Fluor™ 555 Phalloidin (pink). Scale bars 50 µm.

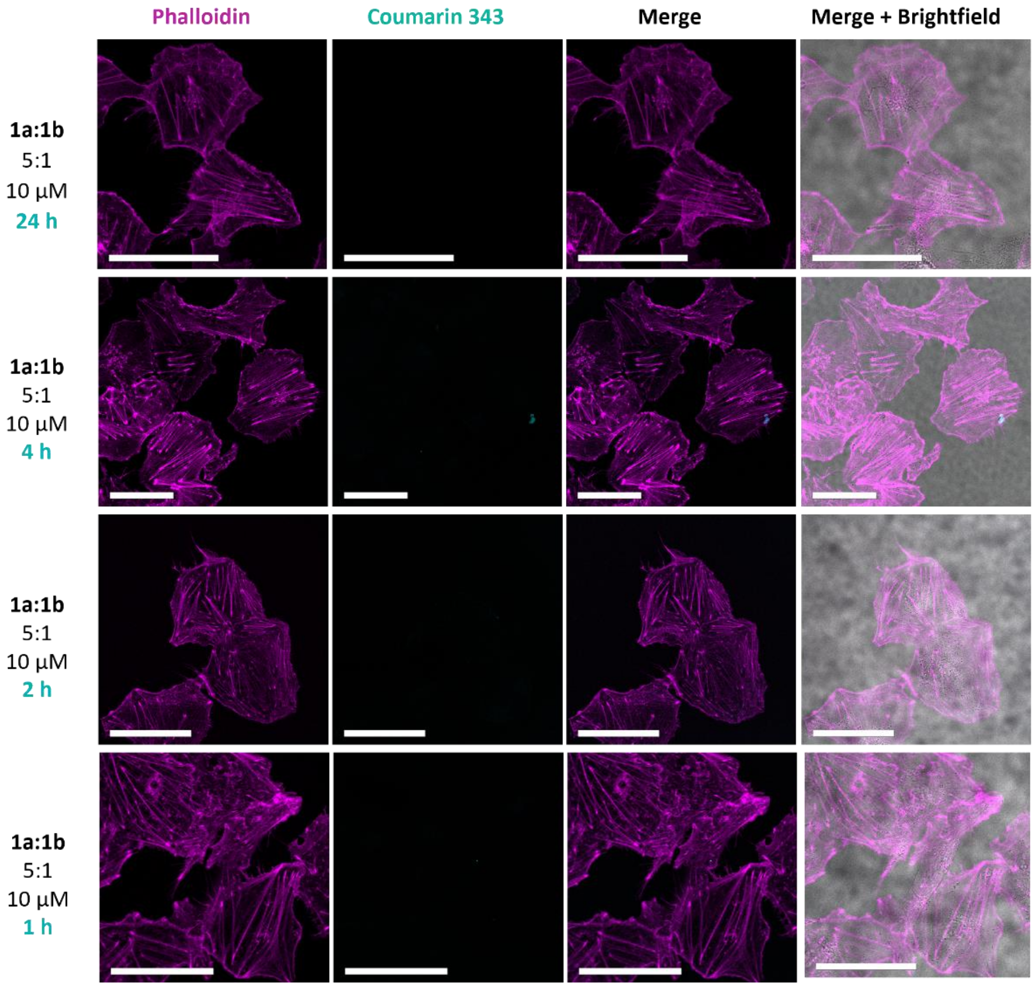

Figure S32 Confocal laser scanning microscopy (CLSM) images of MDA-MB-231 cells after 1, 2, 4 and 24 h of incubation with glutathione-responsive isopeptides **1a** and **1b** (5:1) (cyan) at 10 µM and additional staining with Alexa Fluor™ 555 Phalloidin (pink). Scale bars 50 µm.

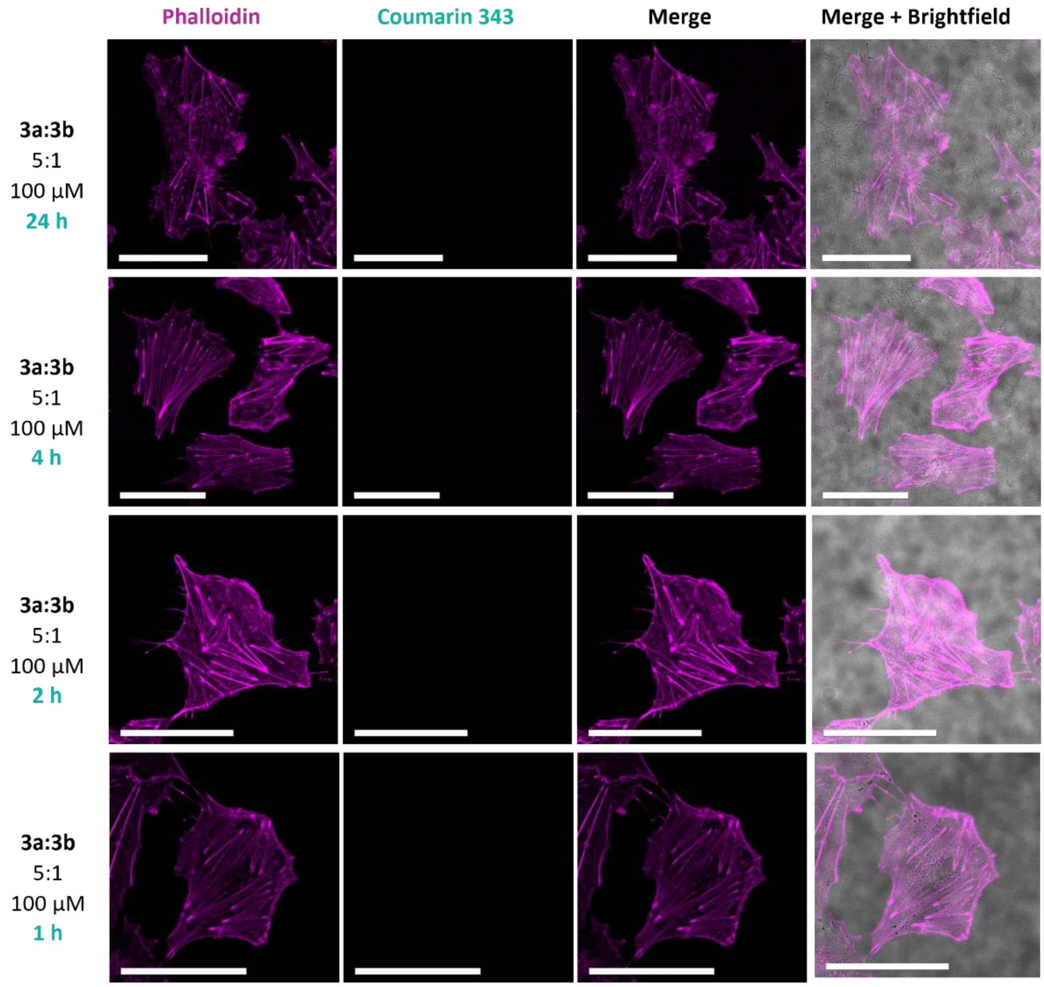

Figure S33 Confocal laser scanning microscopy (CLSM) images of MDA-MB-231 cells after 1, 2, 4 and 24 h of incubation with linear peptides **3a** and **3b** (5:1) (cyan) at 100 µM and additional staining with Alexa Fluor™ 555 Phalloidin (pink). Scale bars 50 µm.

4.5. Analysis of G-/F-actin ratio

MDA-MB-231 cells were seeded at a density of 200,000 cells/well in a 12-well plate and incubated at 37°C overnight to allow them to adhere. On the next day cells were incubated with the *iso*peptide **1a:1b** (5:1) and the linear peptide **3a:3b** (5:1) at 50 µM each for 2 h. Two wells were used as controls, where the old medium was substituted with fresh DMEM/1% DMSO. After the incubation time the G-actin /F-actin assay was performed as described in the manufacturer's protocol. Briefly, cells were lysed by adding 125 µL warm LAS02 buffer to each well (Lysis and F-actin Stabilization Buffer) and harvested using a cell scraper. Samples were homogenized using a Dounce homogenizer before incubating them at 37°C for 10 minutes. Prior to the incubation one of the controls was used for a positive control by adding 1.25 µL of a 100x F-actin Enhancing Solution, which is included in the assay kit, to the cell lysate. After the incubation the samples were centrifuged at 350 x g for 5 minutes to remove cell debris and the supernatant was transferred into ultracentrifugation tubes. To separate F-actin and G-actin the samples were centrifuged at 100,000 x g for 1 h at 37°C. After removing the supernatant and collecting it in fresh tubes, the F-actin in the pellet were depolymerized by adding

100 µL F-actin depolymerization buffer and incubate the samples on ice for 1 h. After incubation 25 µL of a 5x SDS sample buffer were added and samples were stored at -20°C overnight.

On the next day SDS-PAGE was performed using a 4–20% Mini-PROTEAN® TGX™ Precast Protein Gel loading 10 µL sample per well. In addition to the samples, 4 µL Protein Marker VI (10 - 245) as well as G-actin standards corresponding to 10, 20 and 50 ng G-actin, were loaded. The gel was run at constant voltage of 125 V for 90 minutes. After running the gel the proteins were transferred to a PVDF-membrane using methanol free Pierce Western Blot Transfer Buffer at a constant current of 350 mA for 60 minutes. The membrane was washed in TBST buffer (20 mM Tris, 150 mM NaCl, 0.1 % (w/v) Tween 20) before blocking with TBST/ 5% non-fat milk powder at 4°C overnight. The next day, the membrane was washed 3x with TBST before adding the primary antibody anti-actin mouse monoclonal antibody and incubating for 1 h at room temperature. After washing 3x with TBST the secondary antibody Rabbit Anti-Mouse IgG H&L (HRP) was added and the membrane was incubated again for 1 h. After washing 5x with TBST the G-actin on the membrane was visualized using HRP substrate and imaged using BioRad ChemiDoc Imaging System. For calculations of the G-/F-actin ratio the intensities of the G-actin bands were calculated using ImageJ.

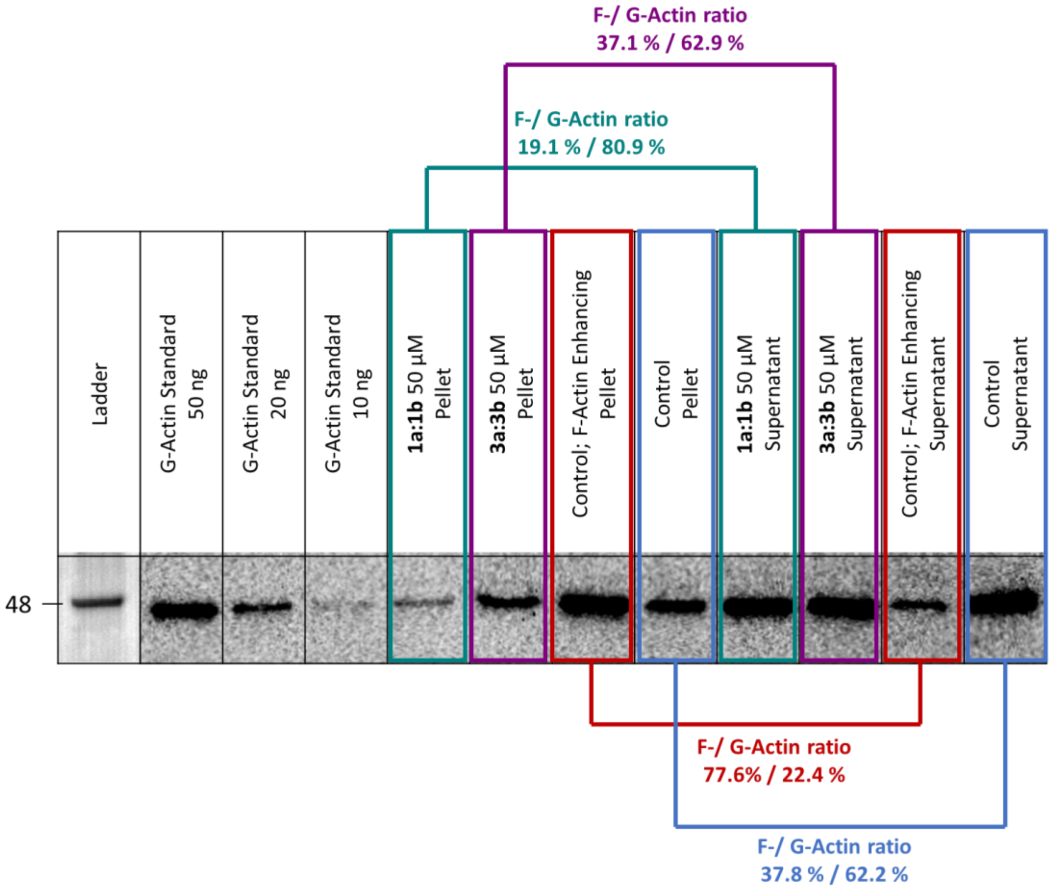

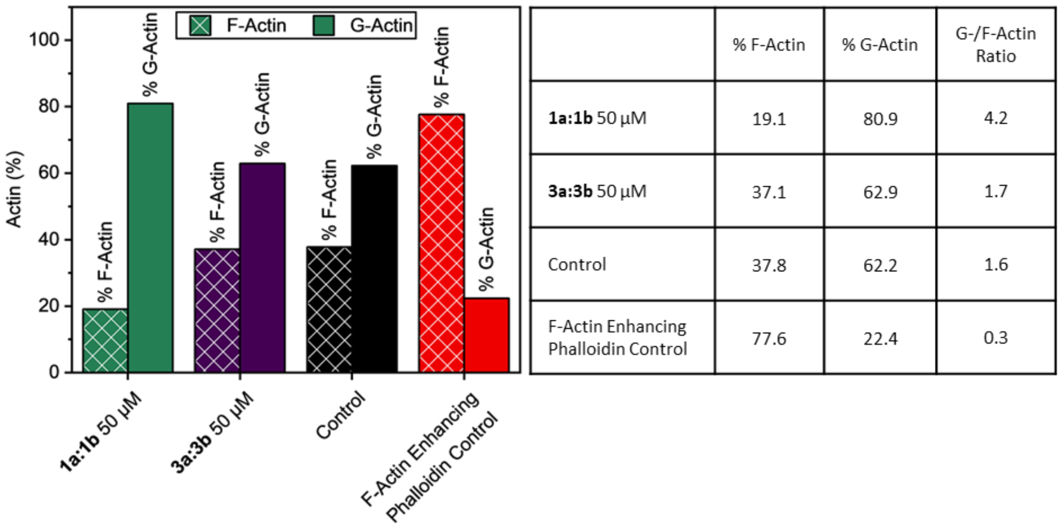

	% F-Actin	% G-Actin	G-/F-Actin Ratio
**1a:1b** 50 µM	19.1	80.9	4.2
**3a:3b** 50 µM	37.1	62.9	1.7
Control	37.8	62.2	1.6
F-Actin Enhancing Phalloidin Control	77.6	22.4	0.3

Figure S34 Western blot analysis of G-/F-actin fractionation of MDA-MB-231 cells. a) Representative western blot showing G-actin and F-actin distribution after fractionation. G-actin standards (10 ng, 20 ng and 50 ng) are included for reference. Samples include an untreated control, F-actin-enhancing condition, and treatments with isopeptides **1a:1b** and linear peptides **3a:3b** (50 µM each). Pelleted fractions correspond to F-actin content, while supernatant fractions represent G-actin content. The G-/F-actin ratio for cells treated with **1a:1b** is 4.2 and for cells treated with the linear control peptides **3a:3b** is 1.7. Untreated control cells have a G-/F-Actin ratio of 1.6. The molecular weight marker (ladder) is shown on the left. Actin (40 kDa) bands were detected slightly below the 48 kDa band. **b)** Ratio of G- and F-actin for each treatment.

4.6. Cell viability assay

MDA-MB-231 or A549 cells were seeded at a density of 2,500 cells/well in a white half area 96-well plate and incubated at 37°C overnight to allow them to adhere. The following day, different concentrations of the glutathione-responsive isopeptides **1a:1b,** the glutathione-responsive non-assembly-inducing isopeptide **1c** or the other control compounds. For each condition, quadruplicates were performed. Cells were incubated with 50 µL of the sample for 4 h. After the treatment, CellTiter-Glo® Assay solution (50 µL) was added to each well, and the plate was placed on an orbital shaker for 2 minutes and subsequently incubated 10 minutes at room temperature. Luminescence was measured using a Promega GloMax®-Multi Detection System using the manufacturer's protocol.

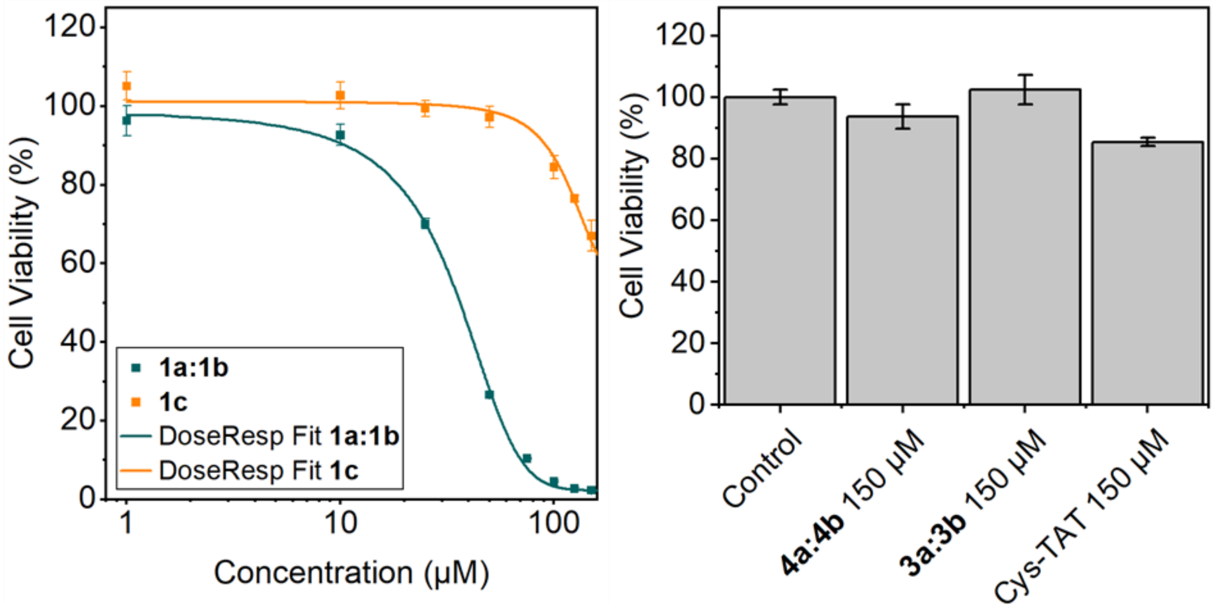

Figure S35 Cell viability of MDA-MB-231 cells incubated with **1a:1b** (green curve) or **1c** (orange curve) for 4 h (left) and with the other control compounds (right). The IC_{50} value of **1a:1b** for MDA-MB-231 cells is 36.4±3.1 µM.

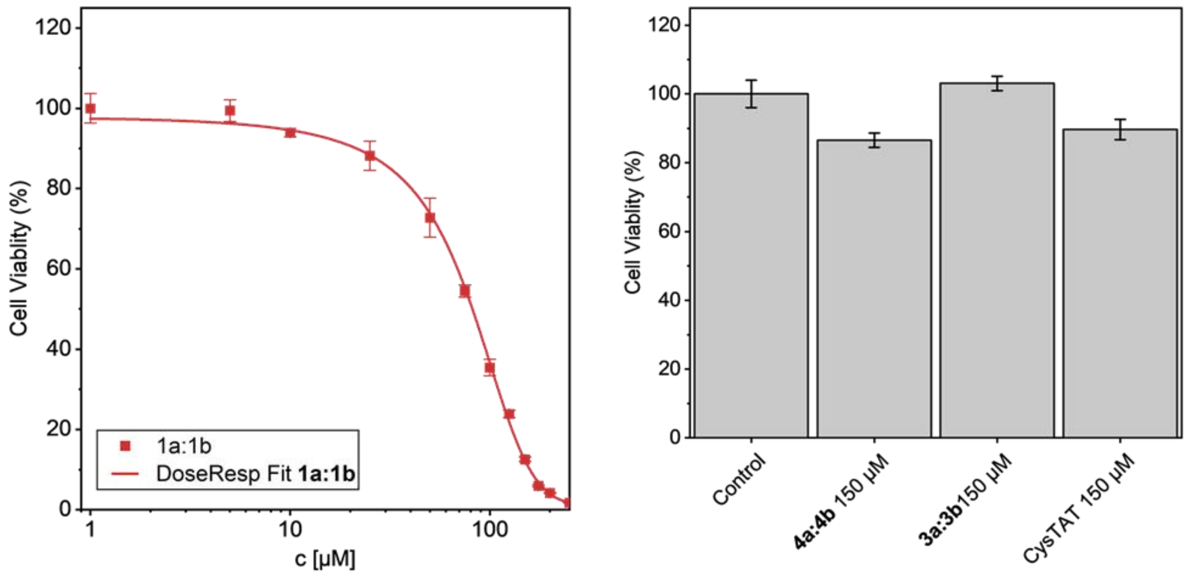

Figure S36 Cell viability of A549 cells incubated with **1a:1b** (red curve) for 4 h (left) and with the control compounds (right). The IC_{50} value of **1a:1b** for A549 cells is 81.7±7.1 µM.

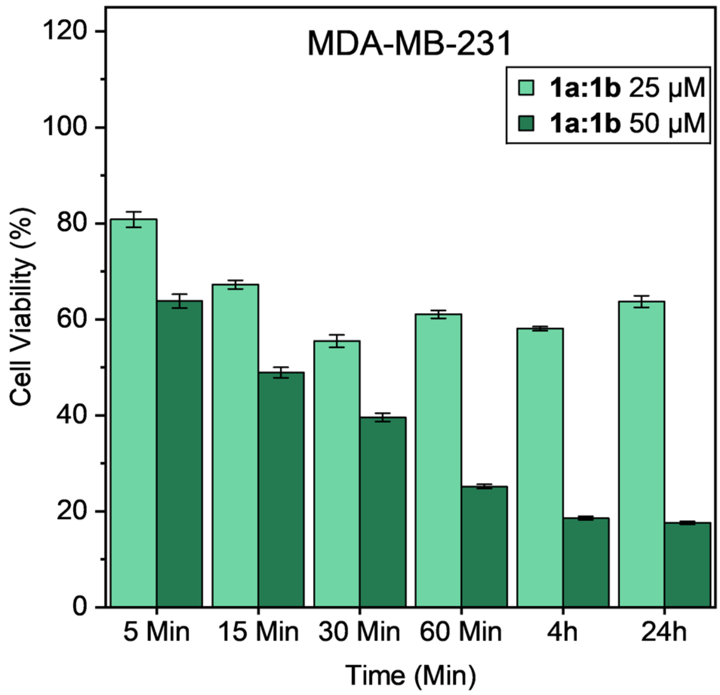

Figure S37 Cell viability of MDA-MB-231 cells incubated with **1a:1b** at 25 or 50 µM for 5 min, 15 min, 30 min, 60 min, 4 and 24 hours.

4.7. Analysis of apoptosis

4.7.1. Caspase-3/7 Glo Assay

Caspase-3/7 Glo Assay is used to observe the apoptotic nature of cell death of MDA-MB-231 cells upon treatment with *iso*peptide **1a:1b**. MDA-MB-231 cells were seeded at a density of 10,000 cells/well in a 96-well half-area white plate and incubated at 37°C, 5% CO_2 overnight to allow them to adhere. The next day cells were incubated with isopeptides **1a:1b** (5:1) in DMEM/DPBS/DMSO (90%/9%/1%) at 25 and 100 µM for different incubation times (5, 10, 15, 30, 45 minutes). As control cells were incubated with **3a:3b** (5:1) in DMEM/DPBS/DMSO (90%/9%/1%) at 100 µM for different incubation times (15 and 45 minutes). After the designated incubation time the 96 well plate was removed from the incubator and equilibrated to room temperature before adding 50 µL Caspase-Glo 3/7 reagent to each well. After shaking the plate gently on a plate shaker for 30 seconds, the samples were incubated at room temperature for 60 minutes before measuring luminescence intensity using the luminescence intensity readout at a Tecan Spark.

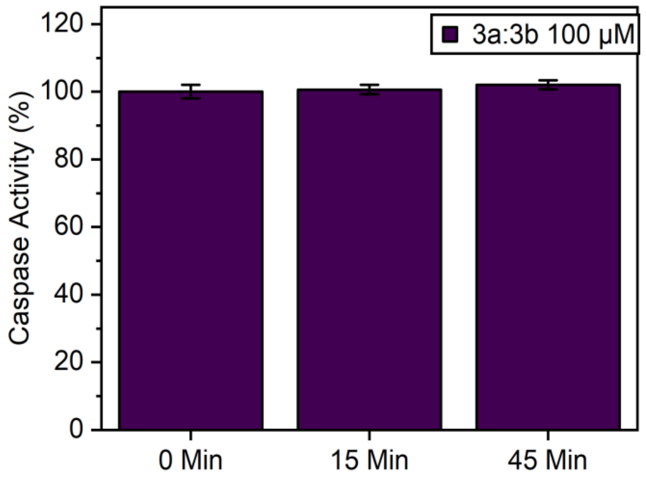

Figure S38 Caspase-Glo Assay results showing caspase activation in MDA-MB-231 cells after incubation with linear peptides **3a:3b** at 100 µM at different time points. No change in caspase-3/7 activity compared to untreated control cells was detected. Data represent mean ± s.e.m. (n = 4).

4.7.2. Annexin V staining

Annexin V staining is used to observe apoptotic cell death upon treatment of MDA-MB-231 cells with *iso*peptide **1a:1b** at 50 µM. MDA-MB-231 cells were seeded at a density of 20,000 cells/well in an 8-well Ibidi plate and incubated at 37°C, 5% CO2 overnight to allow them to adhere. The next day cells were incubated with *iso*peptides **1a:1b** (5:1) in DMEM/DPBS/DMSO (90%/9%/1%) at 50 µM for 1 h. After washing with 1x binding buffer cells were then incubated with Annexin V FITC (25 µL/mL) and propidium iodide (25 µL/mL), both obtained from Apoptosis Detection Kit by Invitrogen. Cells were kept at room temperature for 20 minutes before removing the staining solution and washing them 1x with binding buffer. The washing solution was replaced with fresh 1x binding buffer (200 µL) and cells were imaged using a Leica Stellaris® 8 microscope (40x glycerol immersion objective).

4.7.3. Caspase-3/7 live cell imaging

MDA-MB-231 cells were cultured in an 8-well confocal plate at a density of 20,000 cells per well in DMEM and allowed to adhere overnight at 37°C, 5% CO2. The medium was removed and the cells were stained using BioTracker NucView® 530 Red Caspase-3 Dye according the manufacturer's protocol. Immediately before imaging, the sample of **1a:1b** at 50 µM in DMEM/DPBS/DMSO (90%/9%/1%) was introduced into a well. The cells were imaged live using an incubator-equipped Leica Stellaris® 8 microscope (40x glycerol immersion objective) with fast lifetime contrast (FALCON) module (Leica Microsystems GmbH). The incubator (okolab) is set and held constant at 37 °C, 5% CO2 and a relative humidity of 90% throughout all measurements.

5. Analysis of Metabolic Disruption

5.1. Mito Stress test

The effect of the compounds on the metabolism of MDA-MB-231 cells or A549 cells was investigated using the Seahorse XFe96 Analyzer (Agilent Technologies). The Seahorse XF Cell Mito Stress Test Kit was used following the manufacture's protocol to determine the respiratory activity of the cells. Cells were seeded at a density of 20,000 cell/well one day before the assay and incubated overnight at 37°C, 5% CO_2 in DMEM, high glucose supplemented with 10% FBS. The cartridge for sample loading was loaded with calibration solution (200 µL per well) and incubated at 37°C in a non-CO_2 incubator. On the day of the assay, Seahorse XF DMEM, pH 7.4 was supplemented with pyruvate (1 mM), glutamine (2 mM), and glucose (10 mM). Cells were washed twice with XF DMEM (2x 100 µL) and the medium was substituted with fresh XF DMEM (180 µL) and placed in a 37°C non-CO_2 incubator for 45 to 60 minutes before loading the plate into the instrument. Assay components of the Mito Stress Test Assay were prepared as described in the manufacturer's protocol. Briefly, Oligomycin was resuspended in 630 µL, Carbonyl cyanide-p-trifluoromethoxyphenylhydrazone (FCCP) in 720 µL and Rotenone/ Antimycin A (Rot/AA) in 540 µL supplemented XF DMEM and vortexed vigorously to ensure complete dissolution. Stocks were diluted to 3 ml total; Oligomycin: 450 µL stock in 2,550 µL XF DMEM (final concentration 1.5 µM); FCCP: 300 µL in 2,700 µL XF DMEM (final concentration 1 µM); Rot/AA: 300 µL in 2,700 µL XF DMEM (final concentration 0.5 µM). Samples were prepared at 10x stocks with 10% DMSO in XF DMEM. The cartridge was prepared by loading the samples (20 µL) in Port A of the designated wells and the components of the Mito Stress Test Assay in Ports B-D of each well. The cartridge and 96-well plate with the cells were loaded in the instrument and the content of the ports was injected sequentially according to the specific assay. The oxygen concentration, to monitor the oxygen consumption rate (OCR), was constantly measured throughout the entire time of the assay. The Mito Stress Assay were performed in two independent experiments with n=4. Bar graphs are shown for the mean ± standard error of the mean (s.e.m.) for raw and processed data. For the processed data, all values were set to 100% OCR for the last measurement prior to sample addition before calculating the results. Additionally, the values were normalized to the control for each independent experiment, before calculating the mean value and SEM. Statistical significance was calculated by ANOVA with a Tukey post hoc test and was defined as *$p < 0.05$, **$p < 0.01$, ***$p < 0.001$.

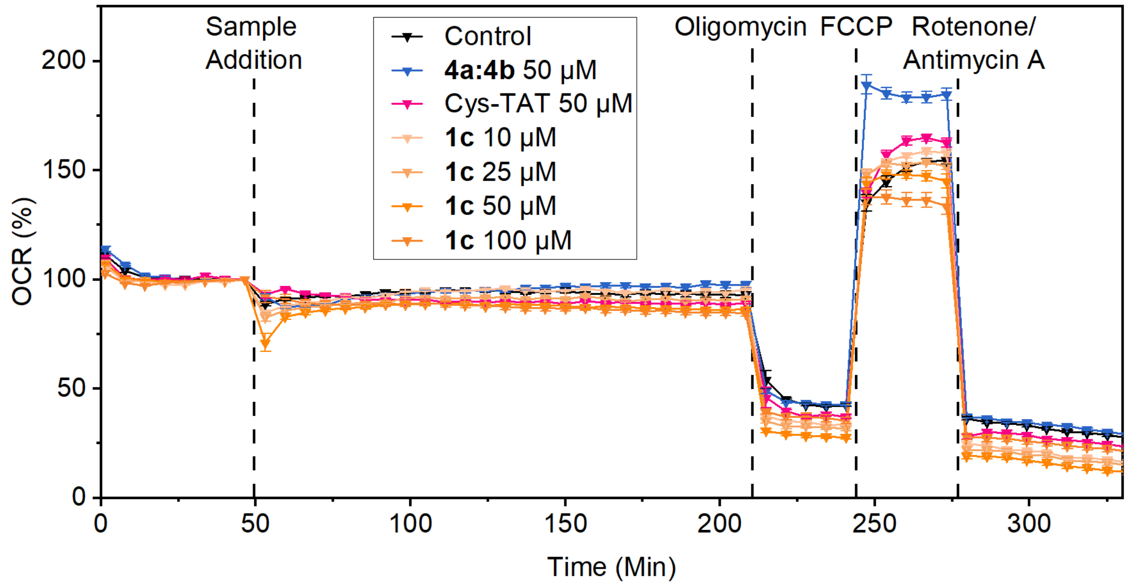

Figure S39 Effect on the oxygen consumption rate (OCR) of MDA-MB-231 cells treated with the control compounds **1c, 4a:4b** and Cys-TAT for 4 hours total. During the last 1.5 hours Mito Stress Test was performed. At different time points specific modulators of the electron transport chain (ETC) were added to investigate the influence of the samples on cellular respiration in more detail: (1) oligomycin inhibits ATP synthase, (2) FCCP (carbonyl cyanide 4-(trifluoromethoxy)phenylhydrazone) disrupts the mitochondrial membrane potential, (3) rotenone inhibits complex I and antimycin A inhibits complex III of the ETC.27 The last measurement before treatment injection of the compound is set as 100%. Data are presented as mean ± s.e.m., n ≥ 8.

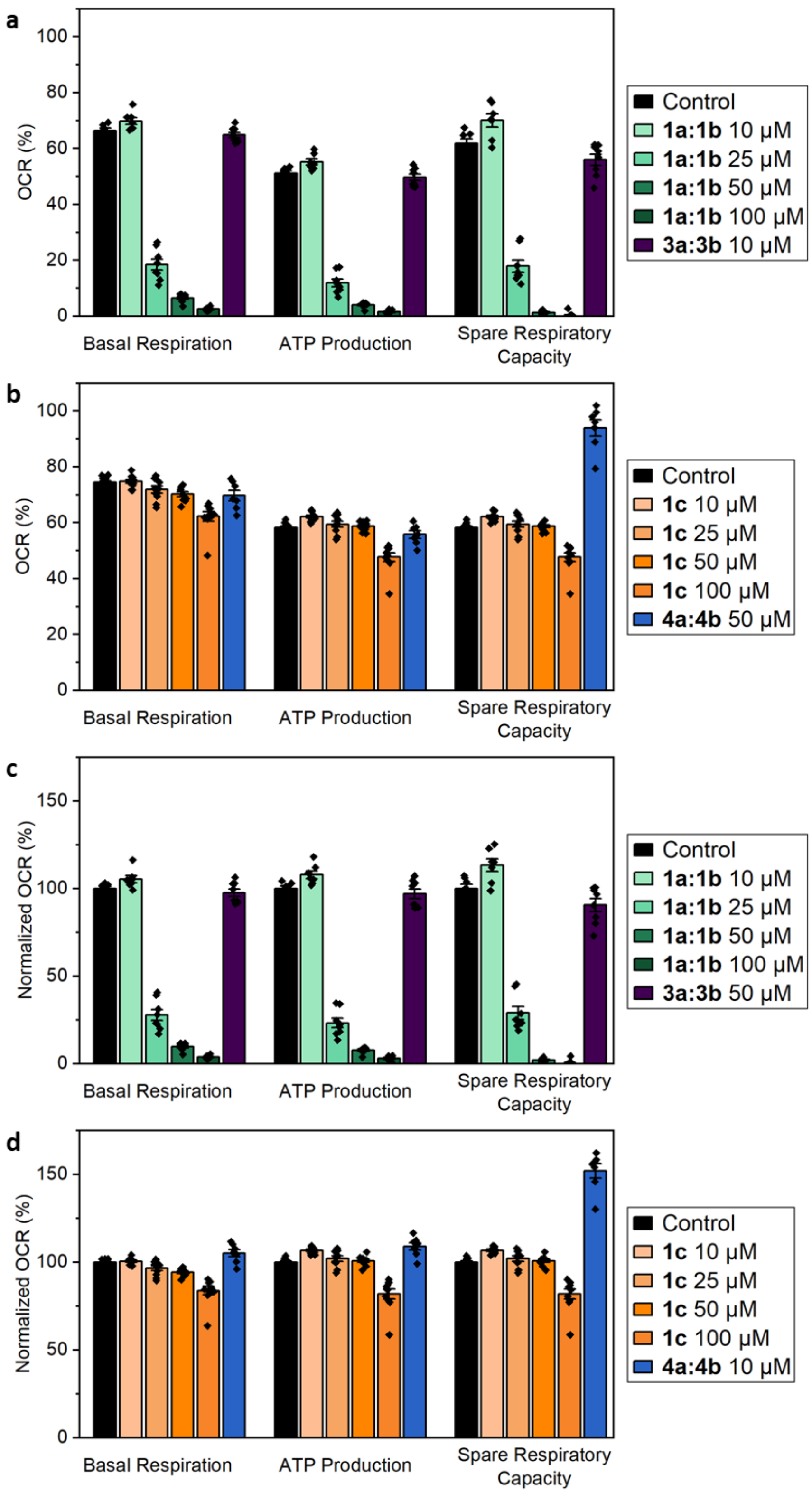

Figure S40 Comparison of the effect of **1a:1b**, **1c, 3a:3b** and **4a:4b** on characteristic values of mitochondrial function after 4 h total incubation. Basal Respiration, ATP Production and Spare Respiratory Capacity are shown. Values were calculated using the data from graphs with the last measurement before sample addition set to 100% OCR and are presented as OCR (%) (**a** and **b**). Additionally, the data was normalized to the control of each experiment and data are shown as Normalized OCR (%) (**c** and **d**). Data are presented as mean ± s.e.m., n ≥ 6.

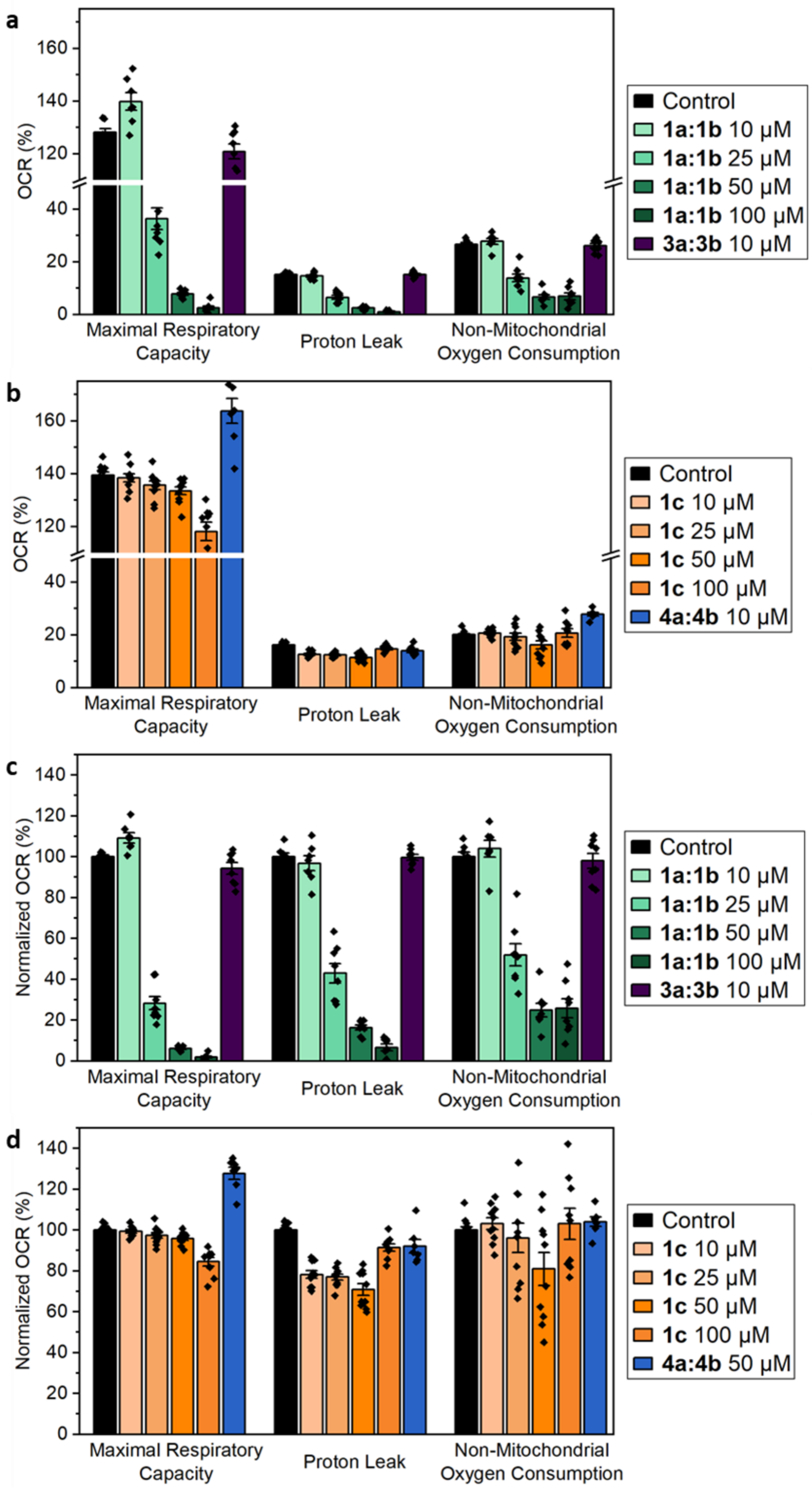

Figure S41 Comparison of the effect of **1a:1b**, **1c, 3a:3b** and **4a:4b** on characteristic values of mitochondrial function after 4 h total incubation. Maximal Respiratory Capacity, Proton Leak and Non-Mitochondrial Oxygen Consumption are shown. Values were calculated using the data from graphs with the last measurement before sample addition set to 100% OCR and are presented as OCR (%) (**a** and **b**). Additionally, the data was normalized to the control of each experiment and data are shown as Normalized OCR (%) (**c** and **d**). Data are presented as mean ± s.e.m., n ≥ 6.

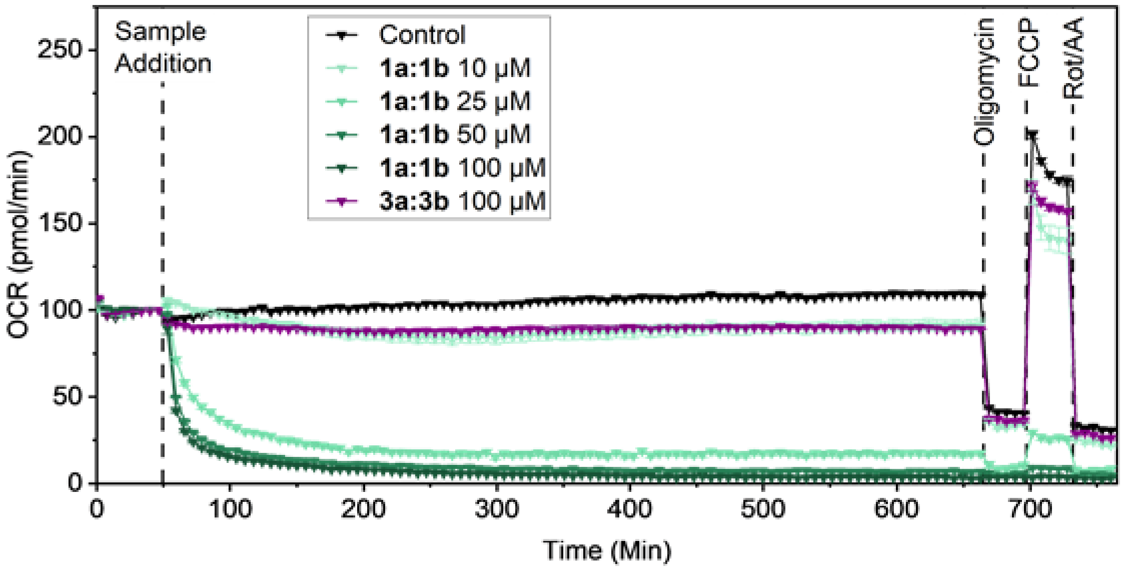

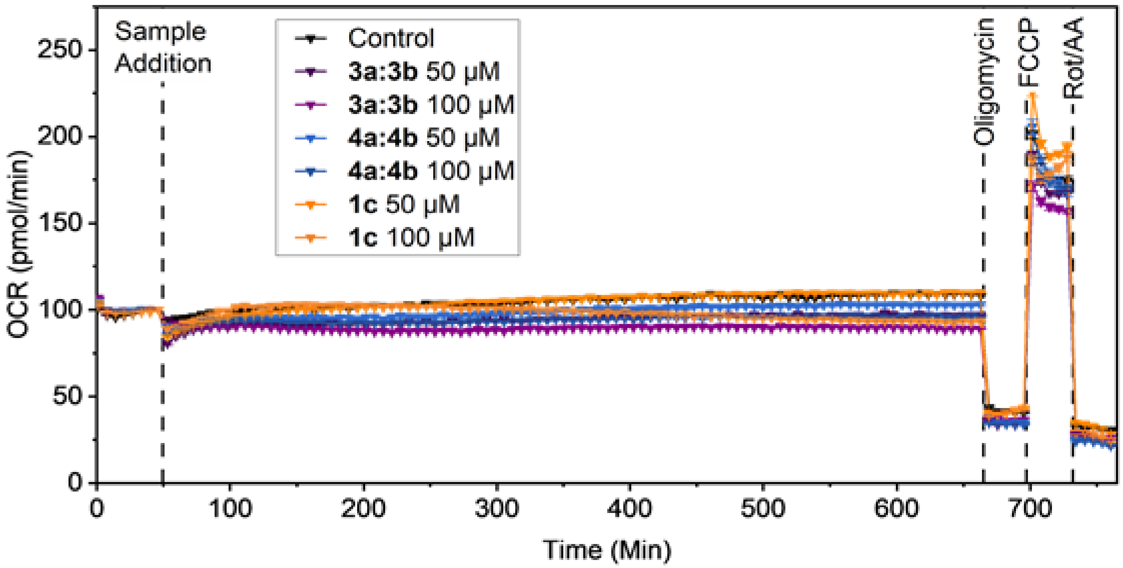

Figure S42 Effect on the oxygen consumption rate (OCR) of MDA-MB-231 cells treated with the isopeptides **1a:1b** or the linear peptides **3a:3b** (upper graph) or the control compounds **1c**, **4a:4b** and additional concentrations of **3a:3b** (lower graph) for 12 hours total. During the last 90 minutes the Mito Stress Test was performed. At different time points specific modulators of the electron transport chain (ETC) were added to investigate the influence of the samples on cellular respiration in more detail: (1) oligomycin inhibits ATP synthase, (2) FCCP (carbonyl cyanide 4-(trifluoromethoxy)phenylhydrazone) disrupts the mitochondrial membrane potential, (3) rotenone inhibits complex I and antimycin A inhibits complex III of the ETC. The last measurement before treatment injection of the compound is set as 100%. Data are presented as mean ± s.e.m., n ≥ 4.

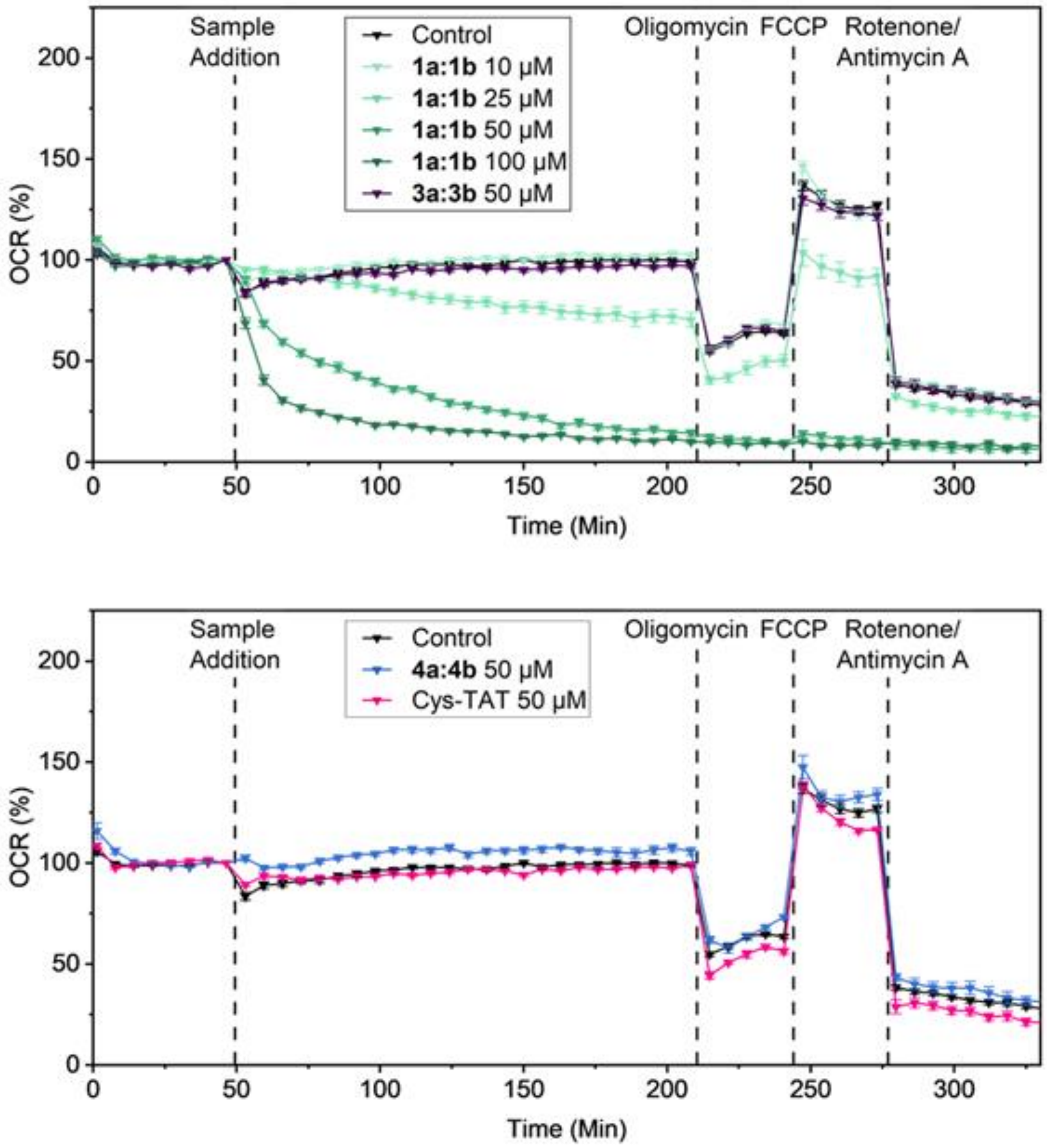

Figure S43 Effect on the oxygen consumption rate (OCR) of A549 cells treated with the isopeptides **1a:1b** or control compounds **1c, 4a:4b** and Cys-TAT for 4 hours total. During the last 1.5 hours Mito Stress Test was performed. At different time points specific modulators of the electron transport chain (ETC) were added to investigate the influence of the samples on cellular respiration in more detail: (1) oligomycin inhibits ATP synthase, (2) FCCP (carbonyl cyanide 4-(trifluoromethoxy)phenylhydrazone) disrupts the mitochondrial membrane potential, (3) rotenone inhibits complex I and antimycin A inhibits complex III of the ETC. The last measurement before treatment injection of the compound is set as 100%. Data are presented as mean ± s.e.m., n ≥ 7.

6. Tumor Spheroid Experiments

6.1. Evaluation of uptake and toxicity on MDA-MB-231 or A549 spheroids

Prior to treatment, fully developed MDA-MB-231 or A549 spheroids were imaged using a Keyence BZ-X810 fluorescence microscope. Subsequently, spheroids were treated with the synthesized and previously characterized compounds (**1a:1a** (100 and 50 µM), **1c** (100 µM), **3a:3b** (100 µM) and **4a:4b** (100 µM) in a final volume of 100 µL/well and incubated at 37°C, 5% CO_2. Untreated spheroids were used as experimental control where the medium was substituted with fresh DMEM containing 1% DMSO. Cellular uptake after 4 h incubation was observed using the GFP filter of a Keyence BZ-X810 microscope. Growth and morphological changes were monitored by brightfield imaging using a Keyence BZ-X810 microscope after 4, 24 or 48 h incubation. At the end of the incubation time spheroids were washed with DPBS to remove treatment residuals and incubated with Propidium Iodide (8 µg/ml; Sigma-Aldrich, P4864). After incubation with the staining solutions spheroids were washed with DMEM FluoroBrite and imaged using either a Keyence BZ-X810 or a Leica Stellaris® 8 microscope. Experiments were carried out on two independent cultures with internal replicates (n≥5).

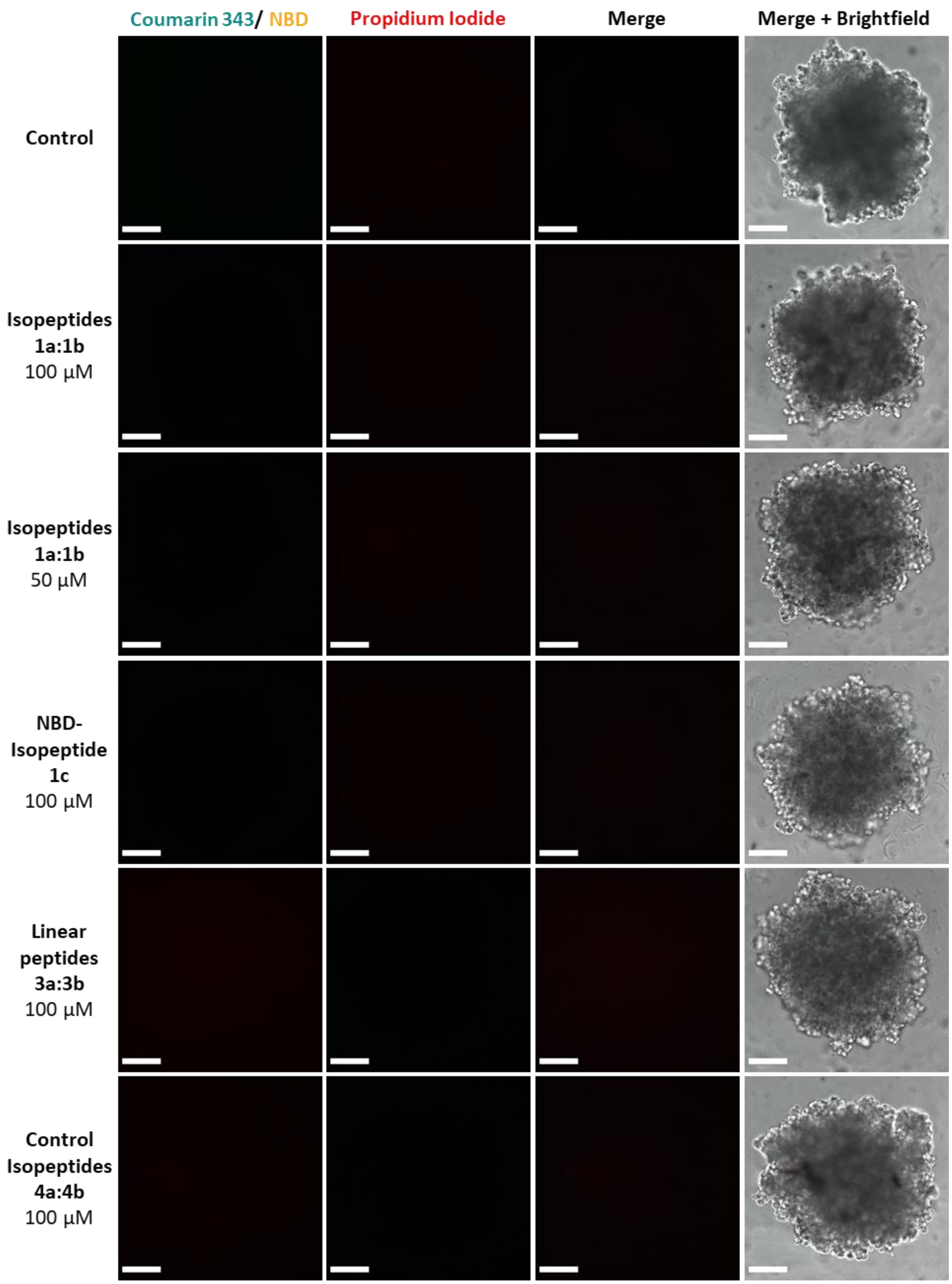

Figure S44 Fluorescence microscopy images of MDA-MB-231 tumor spheroids before treatment. Coumarin 343 (cyan) and propidium iodide (red) fluorescence were imaged using GFP and Cy5 filter settings. Scale bars 100 µm.

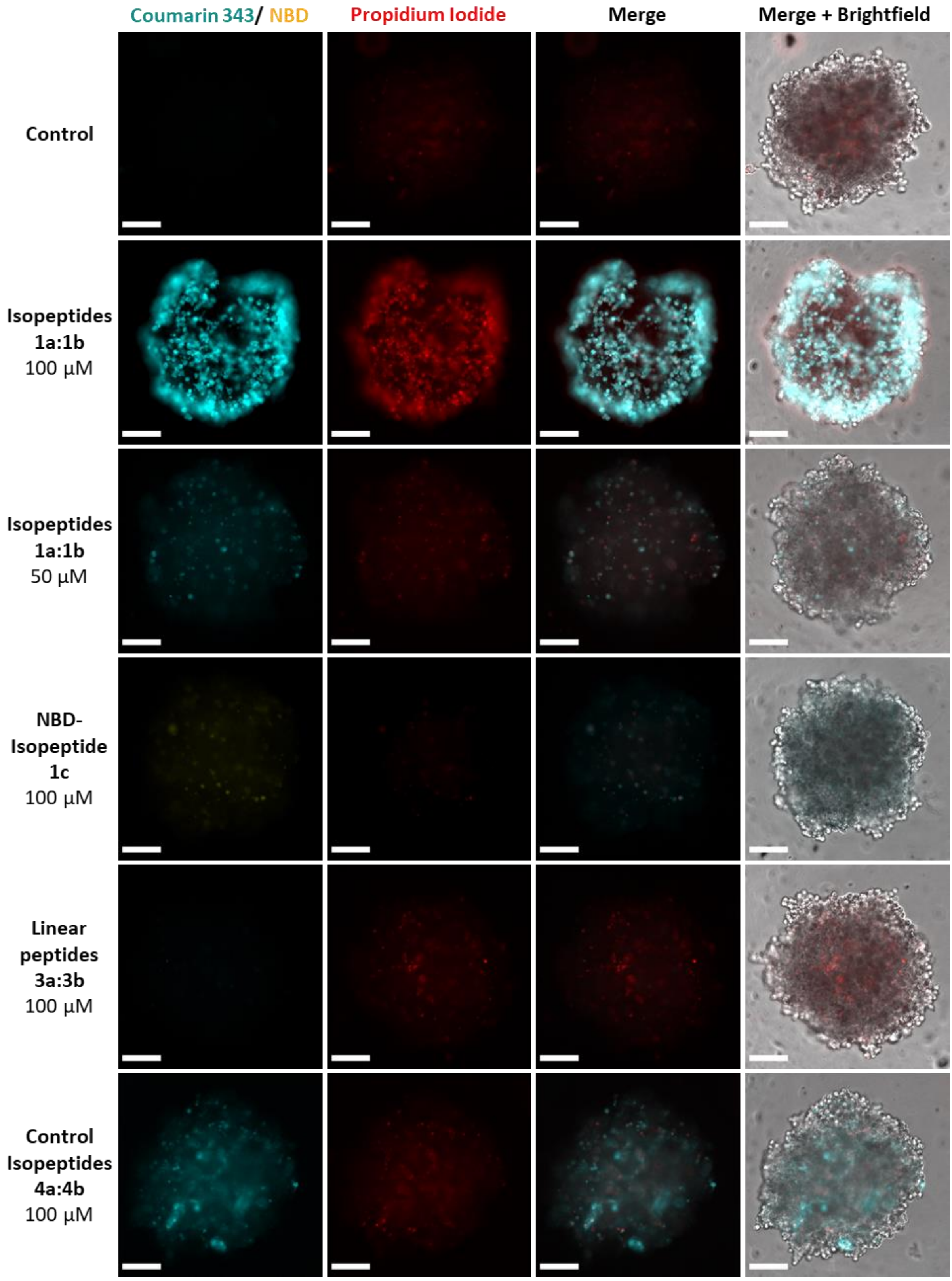

Figure S45 Fluorescence microscopy images of MDA-MB-231 tumor spheroids after 4 h treatment with **1a:1b** at 100 and 50 µM and **1c** , **3a:3b** and **4a:4b** at 100 µM. Coumarin 343 (cyan) or NBD (yellow) and propidium iodide (red) fluorescence were imaged using GFP and Cy5 filter settings. Scale bars 100 µm.

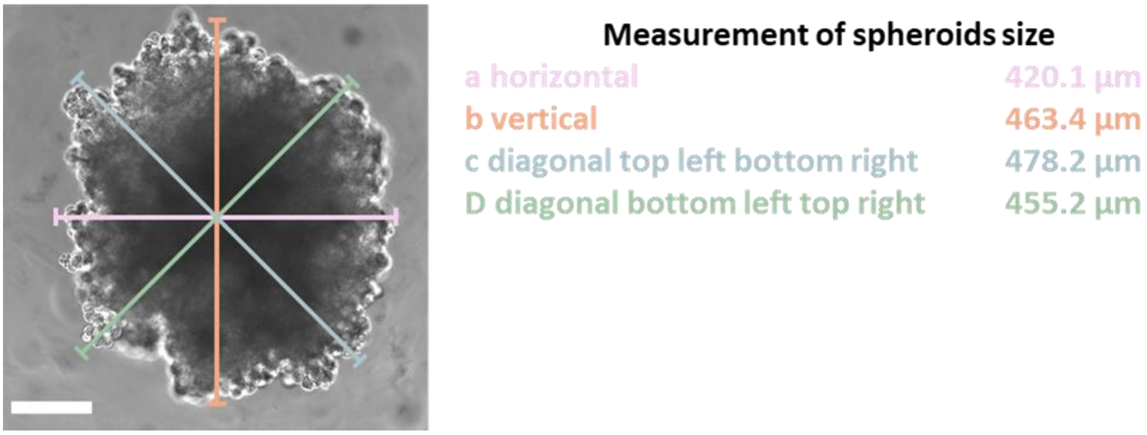

Figure S46 Measurement of spheroid size shown for the example of an untreated control spheroid at t_0. For each spheroid the size was measured at four different positions at t_0 and after 4, 24 or 48 h incubation with compounds. To ensure comparability, the size of each spheroid was measured horizontally, vertically and diagonally. The mean of the four measurements was used to evaluate the change in size within the 4 h incubation time. Images were processed using ImageJ. Scale bar 100 µm.

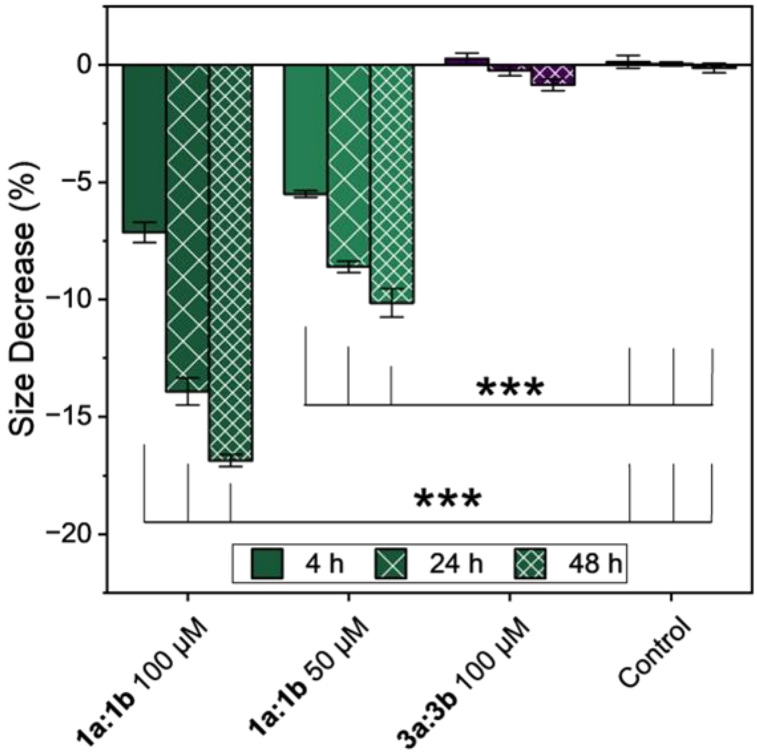

Figure S47 Size decrease of MDA-MB-231 tumor spheroids treated with isopeptides **1a:1b** or linear peptides **3a:3b** for 4, 24 or 48 hours. Spheroid size was measured before and after incubation with glutathione-responsive isopeptides using brightfield images in ImageJ. Diameter of each spheroid was measured at 4 different places (Figure S31). Data are presented as mean ± s.e.m., n=5.

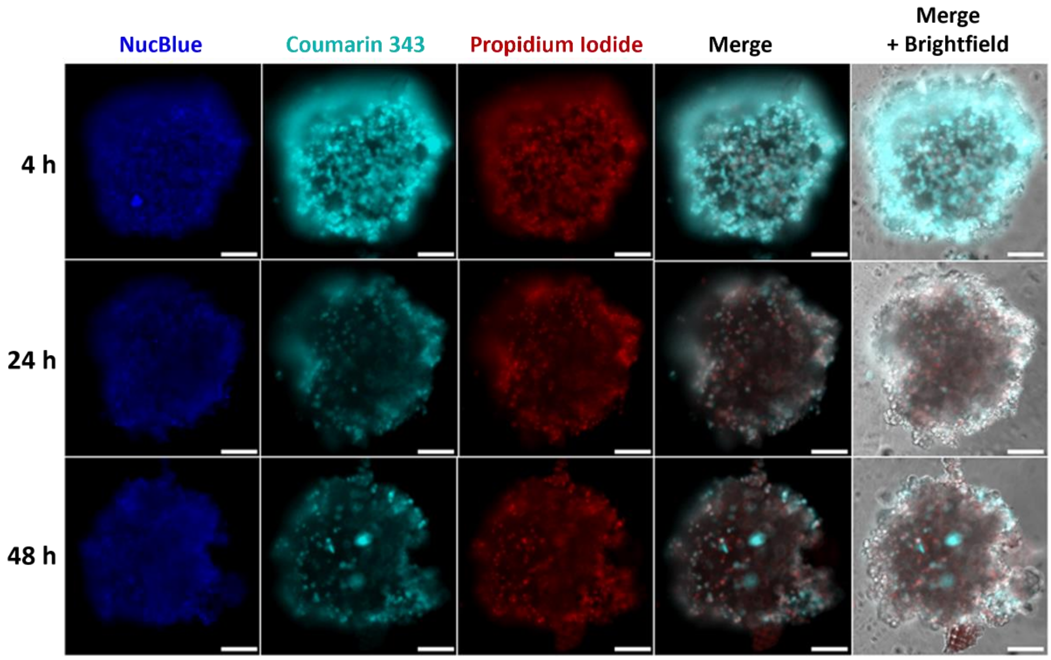

Figure S48 Fluorescence microscopy images of MDA-Mb-231 tumor spheroids after treatment with **1a:1b** at 100 µM for 4, 24 or 48 hours. NucBlue fluorescence (blue), Coumarin 343 (cyan) and propidium iodide (red) fluorescence were imaged using DAPI, GFP and Cy5 filter settings, respectively. Scale bars 100 µm.

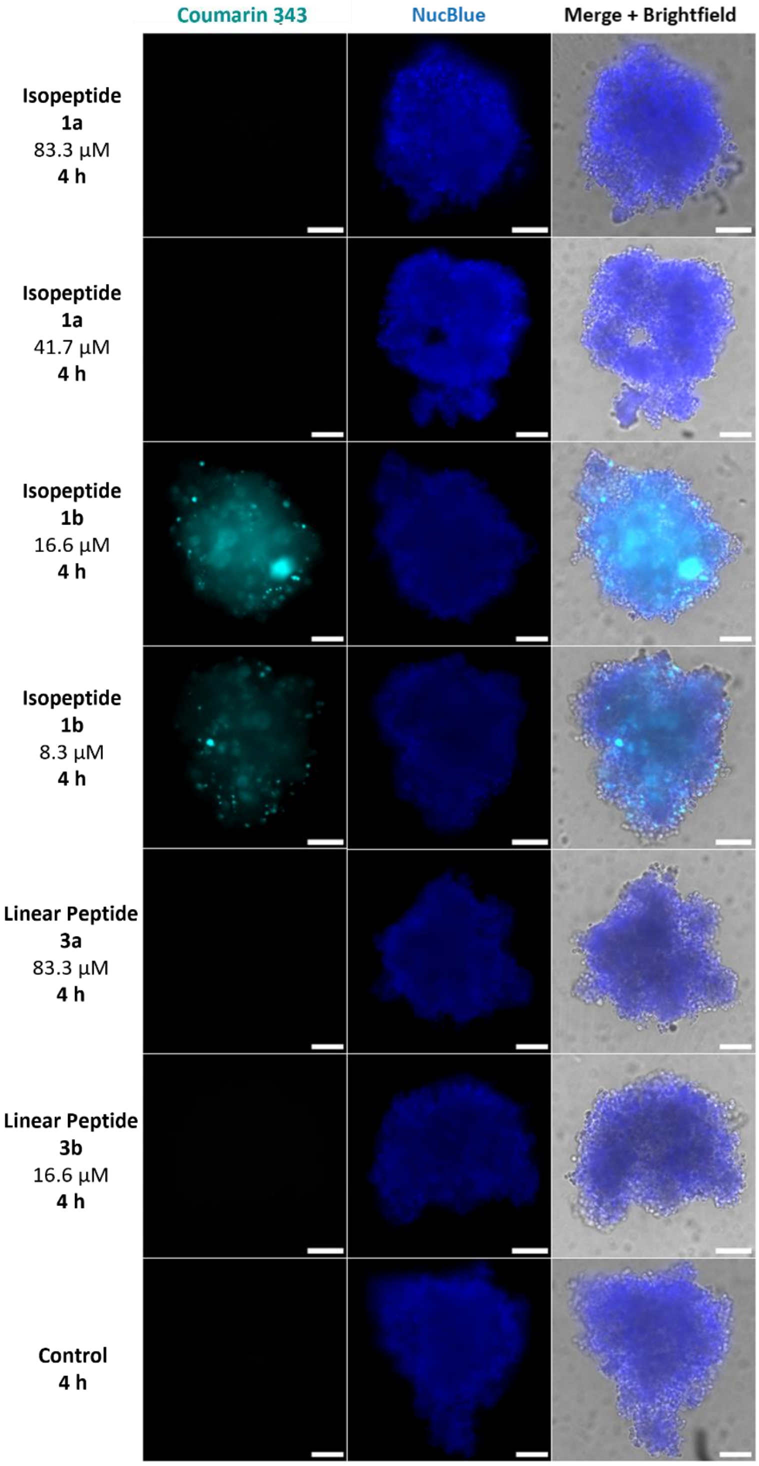

Figure S49 Fluorescence microscopy images of MDA-MB-231 tumor spheroids after treatment with **1a**, **1b**, **3a** or **3b** individually for 4 h. Coumarin 343 (cyan) and NucBlue (blue) fluorescence were imaged using GFP and DAPI filter settings. Scale bars 100 µm.

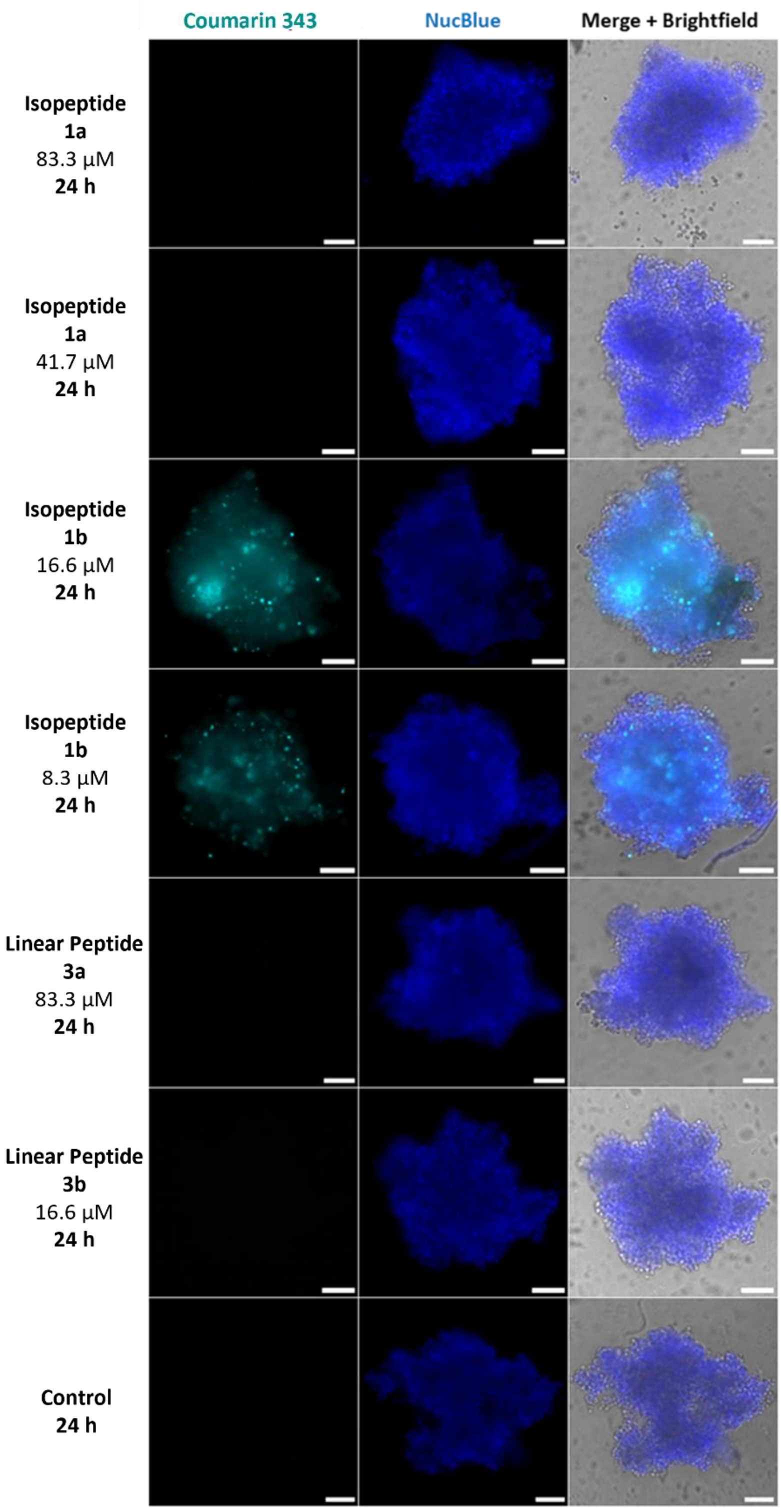

Figure S50 Fluorescence microscopy images of MDA-MB-231 tumor spheroids after treatment with **1a**, **1b**, **3a** or **3b** individually for 24 h. Coumarin 343 (cyan) and NucBlue (blue) fluorescence were imaged using GFP and DAPI filter settings. Scale bars 100 µm.

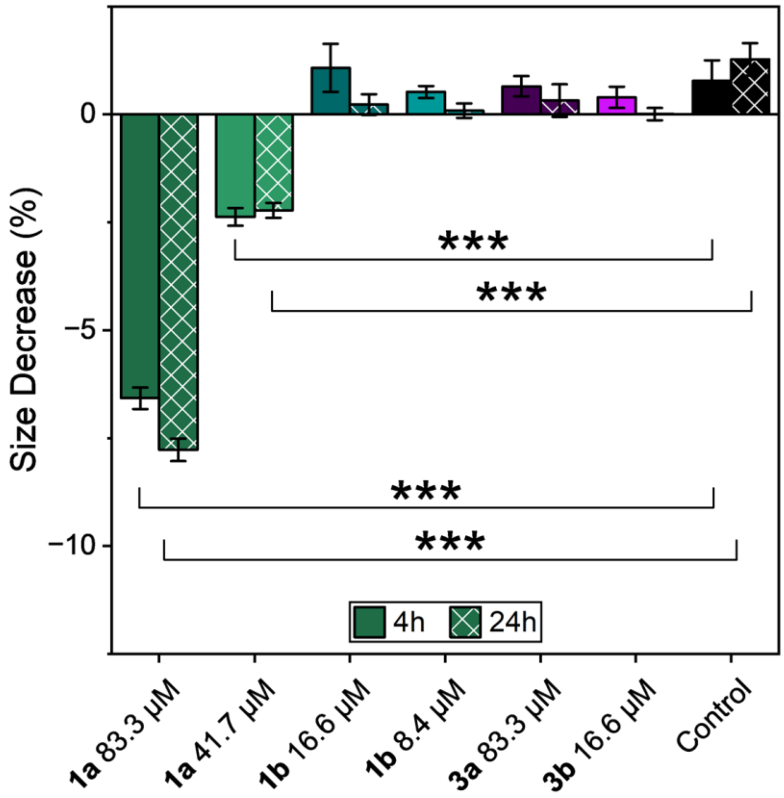

Figure S51 Size decrease of MDA-MB-231 tumor spheroids treated with isopeptide **1a** or **1b** alone, as well as linear peptide **3a** or **3b** alone for 4 or 24 hours. The concentrations of the individual peptides reflect their respective concentration in the previously investigated 5:1 ratios of either isopeptides **1a:1b** or linear peptides **3a:3b**. Spheroid size was measured before and after incubation with glutathione-responsive isopeptides using brightfield images in ImageJ. Diameter of each spheroid was measured at 4 different places (Figure S31). Data are presented as mean ± s.e.m., n=4.

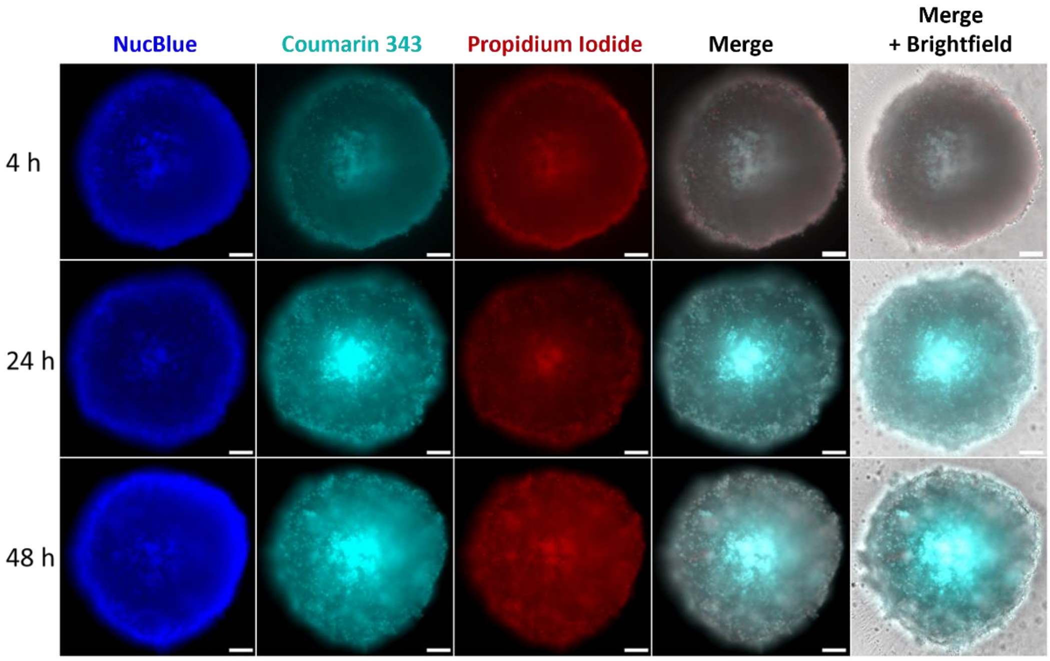

Figure S52 Fluorescence microscopy images of A549 tumor spheroids after treatment with **1a:1b** at 100 µM for 4, 24 or 48 hours. NucBlue fluorescence (blue), Coumarin 343 (cyan) and propidium iodide (red) fluorescence were imaged using DAPI, GFP and Cy5 filter settings, respectively. Scale bars 100 µm.

6.2. Slicing of spheroids

Spheroids were prepared and treated as described in 6.1. Before transferring the spheroids gelatin was dissolved in warm water (175 mg/mL) and transferred into a small plastic container, where it was allowed to solidify. After incubation spheroids were washed with DPBS and transferred on top of solidified gelatin and excess buffer was carefully removed. Spheroids were immobilized by carefully adding a warm drop of gelatin on top and allowing it to solidify. On top of the spheroids a second layer of gelatin was added before the plastic box was stored at -80°C overnight. The next day the gelatin cubes were sliced using a Cryostat Leica CS3050 S and slices of 16 µm were collected. Slices of treated spheroids of MDA-MB-231 and A549 cells were then imaged using a Keyence BZ-X810 fluorescence microscope and a 20x LD PH lens.

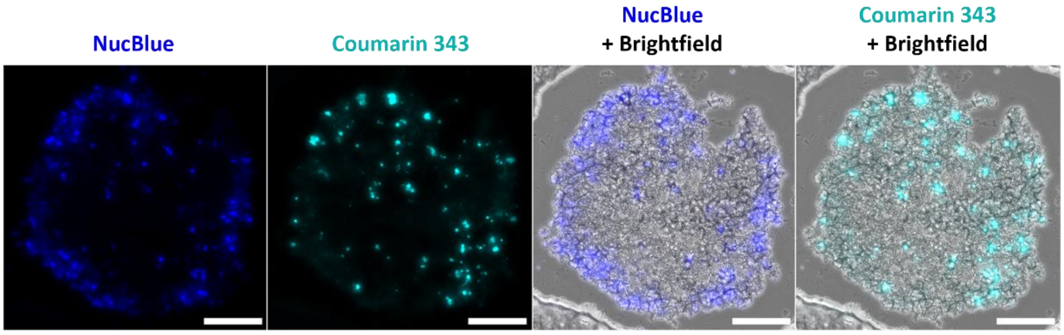

Figure S53 Slices of MDA-MB-231 tumor spheroid. Upon incubation with 1a:1b at 100 µM for 4 h, MDA-MB-231 spheroids were embedded in gelatin. Slices of 16 µm thickness were obtained from the gelatin blocks which were then imaged using Keyence BZ-X810. Scale bar 100 µm.

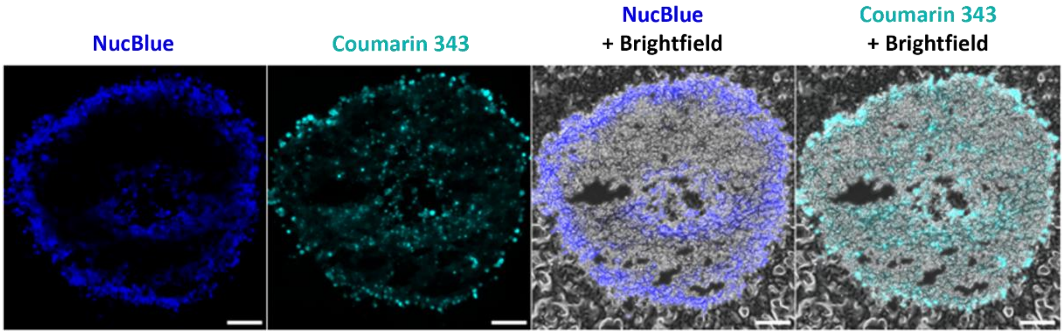

Figure S54 Slices of A549 tumor spheroid. Upon incubation with 1a:1b at 100 µM for 4 h, A549 spheroids were embedded in gelatin. Slices of 16 µm thickness were obtained from the gelatin blocks which were then imaged using Keyence BZ-X810. Scale bar 100 µm.

References

1. Chagri, S.; Burgstaller, A.; Schirra, C.; Link, J.; Zhou, Z.; Roth, P.; Meyer, R.; Fetzer, J.; Ren, Y.; Si, S.; Mazzotta, F.; Wagner, M.; Lieberwirth, I.; Landfester, K.; Ng, D. Y. W.; Staufer, O.; Weil, T., Synthetic intracellular nanostructures enhance cytotoxic T cell function via assembly-driven chemical engineering. *ChemRxiv* **2024**.
